# Changes in maize transcriptome in response to maize Iranian mosaic virus infection

**DOI:** 10.1371/journal.pone.0194592

**Published:** 2018-04-10

**Authors:** Abozar Ghorbani, Keramatollah Izadpanah, Ralf G. Dietzgen

**Affiliations:** 1 Plant Virology Research Center, College of Agriculture, Shiraz University, Shiraz, Iran; 2 Queensland Alliance for Agriculture and Food Innovation, The University of Queensland, St. Lucia, Queensland, Australia; National Botanical Research Institute CSIR, INDIA

## Abstract

**Background:**

Maize Iranian mosaic virus (MIMV, genus *Nucleorhabdovirus*, family *Rhabdoviridae*) causes an economically important disease in maize and other gramineous crops in Iran. MIMV negative-sense RNA genome sequence of 12,426 nucleotides has recently been completed. Maize Genetics and Genomics database shows that 39,498 coding genes and 4,976 non-coding genes of maize have been determined, but still some transcripts could not be annotated. The molecular host cell responses of maize to MIMV infection including differential gene expression have so far not been elucidated.

**Methodology/Principal findings:**

Complementary DNA libraries were prepared from total RNA of MIMV-infected and mock-inoculated maize leaves and sequenced using Illumina HiSeq 2500. Cleaned raw transcript reads from MIMV-infected maize were mapped to reads from uninfected maize and to a maize reference genome. Differentially expressed transcripts were characterized by gene ontology and biochemical pathway analyses. Transcriptome data for selected genes were validated by real-time quantitative PCR.

**Conclusion/Significance:**

Approximately 42 million clean reads for each treatment were obtained. In MIMV-infected maize compared to uninfected plants, 1689 transcripts were up-regulated and 213 transcripts were down-regulated. In response to MIMV infection, several pathways were activated in maize including immune receptor signaling, metabolic pathways, RNA silencing, hormone-mediated pathways, protein degradation, protein kinase and ATP binding activity, and fatty acid metabolism. Also, several transcripts including those encoding hydrophobic protein RCI2B, adenosylmethionine decarboxylase NAC transcription factor and nucleic acid binding, leucine-rich repeat, heat shock protein, 26S proteasome, oxidoreductases and endonuclease activity protein were up-regulated. These data will contribute to the identification of genes and pathways involved in plant-virus interactions that may serve as future targets for improved disease control.

## Introduction

Rhabdoviruses infect plants, invertebrates and vertebrates and are taxonomically classified in the family *Rhabdoviridae*, order *Mononegavirales* [1]. Cyto- and nucleorhabdoviruses infect a wide range of plant species including many agriculturally important crops [2, 3]. Maize Iranian mosaic virus (MIMV) is an economically important virus that infects maize (*Zea mays* L.), wheat (*Triticum aestivum* L.), barley (*Hordeum vulgare* L.), rice (*Oryza sativa* L.) and several other poaceous species in Iran [4]. MIMV is transmitted by the small brown planthopper (*Laodelphax striatellus*) in a persistent-propagative manner [5, 6]. MIMV is classified in the genus *Nucleorhabdovirus*, members of which replicate in plant cell nuclei and accumulate in perinuclear spaces [3]. MIMV is closely related to maize mosaic virus (MMV) based on nucleotide sequence identity [5, 7]. In recent years, an increasing number of studies have focused on molecular host–virus interactions including the plant and vector transcriptome responses [8–10].

Genome-wide gene expression studies may elucidate the effects of viruses on their host and may identify changes in the expression of host genes in response to virus infection [11]. Next-generation high-throughput sequencing and study of transcriptomes can clarify virus infection responses in plants and increase understanding about host responses [12–14]. For example, changes in the maize transcriptome due to biotic and abiotic stresses have been reported including responses to fungi *Bipolaris zeicola* and *Fusarium graminearum*, insects *Rhopalosiphum maidis*, *Graminella nigrifrons* and *Ostrinia furnacalis*, and abiotic factors salinity and jasmonic acid [15–20].

Plant responses to infection have been studied for a range of RNA and DNA viruses including tobacco mosaic virus in tobacco, African cassava mosaic virus in cassava, tomato spotted wilt virus, cucumber mosaic virus and potato virus X in chrysanthemum and capsicum chlorosis virus in capsicum. These studies have provided novel information regarding the up- and down- regulation of host genes during plant-pathogen interactions and determined the pathways involved in plant responses to virus infection [8, 21–23]. The present study contributes to existing sequence resources for maize including National Center for Biotechnology Information (NCBI) and Plants Ensemble (http://plants.ensembl.org/Zea_mays/Info/Index). Besides its economic importance as a food and biofuel crop [24], *Zea mays* is important for studying the mechanisms of pathogen-host interactions as it is a facile model including a large collection of mutant germplasm, large heterochromatic chromosomes, presence of annotated genes, extensive nucleotide diversity, good gene network and pathways database and genic co-linearity within related grasses. These characteristics have positioned this species as a centerpiece for genetic, cytogenetic, and genomic research [24]. Current maize sequence resources show that 136,472 transcripts of maize have been identified that potentially encode 39,498 proteins and 4,976 non- coding genes, some of which of unknown function (www.plants.ensembl.org). Although the functions of many genes and pathways during virus infection in maize remains unknown, our study provides valuable new insights into the potential roles of differentially expressed genes in response to MIMV infection. This information will help us to better understand the distinctive virus defense mechanisms in plants, identify essential host gene products that may be recruited to assist virus replication and movement, and identify candidate genes that might be targeted for control of MIMV infection.

## Materials and methods

### Plant growth, virus inoculation and RNA extraction

Seeds of MIMV-susceptible maize cultivar 704 [25] were sources from the Seed and Plant Improvement Research Division, Fars Research Center for Agriculture and Natural Resources, Shiraz, Iran, germinated in soil, and plants were grown in a greenhouse on a cycle of 16 h light at 30°C and 8 h dark at 25°C. Three days after seed germination, at the two-leaf stage, ten plants were exposed for 3 days to 20 viruliferous planthopper nymphs infected with MIMV isolate ‘Fars’ and had been maintained on MIMV-infected barley plants in an insect-proof cage. Mock-inoculated plants were exposed to 20 planthoppers from a virus-free colony. Total RNA was extracted from 500 mg of leaf tissue from each of the three uninfected and three infected plants 15 days after inoculation when symptoms had appeared, using TRIzol Reagent (Thermo Fisher Scientific) following the manufacturer’s protocol. RNA was dissolved in diethyl pyrocarbonate (DEPC)-treated water, and its quality was assessed by measuring absorption ratio 260/280 nm using a Nanodrop-1000 spectrophotometer (Thermo Scientific, Wilmington, DE, USA) and by 1% agarose gel electrophoresis in Tris/borate/EDTA buffer (TBE).

### Virus detection

MIMV was detected in maize plants by DAS-ELISA [26] using antiserum developed in Plant Virology Research Center, Iran [7] and by RT-PCR using primers MIMV-F (5′-TGCAGGGAAATCTCTGGAGG-3′) and MIMV-R (5′-CCTCATACATTGGCTGGGGA-3′). Complementary DNA was synthesized using M-MLV reverse transcriptase (Thermo Fisher Scientific) and primer MIMV-R. PCR was carried out in 25 μL reactions containing 2 μL of cDNA, 1 μL of each 10μM primer, 0.5 μL of 10μM dNTPs, and 2.5 μL of 10 × PCR buffer; 0.75 μL of 50 μM MgCl2, 0.25 μL (1 U) of Taq DNA polymerase (Cinnagen, Iran) and 17 μL H_2_O. PCR conditions were 94 °C for 5 min, 35 cycles of 94°C for 1 min, 56 °C for 30 s, and 72 for 1 min, and a final extension at 72°C for 7 min. PCR products were electrophoresed in a 1.5% agarose gel in TBE buffer and visualized under UV light after staining with ethidium bromide.

### Library construction, sequencing and RNA-Seq data analysis

Following quality control on an Agilent Technologies 2100 Bioanalyzer using a DNA 1000 chip and Roche’s Rapid library standard Quantification solution and calculator, passed samples were used for library construction (TruSeq RNA Sample Prep Kit v2) and sequenced with 100 bp paired end reads using Illumina HiSeq 2500 (Macrogen, Seoul, South Korea). Contaminating genomic DNA was removed during mRNA purification. Whole transcriptome RNA was enriched by depleting ribosomal RNA and six cDNA libraries were prepared using poly(A) enrichment from the 3 replicates of 2 treatments consisting of MIMV-infected and mock-inoculated maize. Raw sequencing reads were trimmed for quality and adaptors were removed. Clean reads were mapped to the maize reference genome (www.plants.ensembl.org). Analyses of differentially expressed (DE) transcripts were based on the genome-mapped data. Expression profiling and differential gene expression analyses were done using CLC Genomic Workbench version 9 (CLC Bio, Qiagen). DE transcripts were identified using the criteria of fold change ≥2 and false discovery rate (FDR) <0.05. To ensure true representation of fragment gene expression levels, read numbers and gene lengths were normalized using fragments per kilobase of transcript per million fragments mapped (FPKM). Blast2Go software was used for gene ontology analysis. Gene regulatory network and pathway analysis were done using STRINK database (www.string-db.org).

### Validation of DE transcripts by real-time quantitative PCR

RNA-Seq gene expression profiles for five selected genes were validated by RT-qPCR. Ubiquitin-conjugating enzyme and cullin were selected as internal reference genes from the non-DE genes in the RNA-Seq dataset. PCR primers were designed using Primer 3 software in Geneious version R10 and synthesized by GeneWorks (Adelaide, South Australia) (Table 1). First-strand cDNA synthesis was done using oligo dT primers and Superscript III First-strand cDNA synthesis kit (Life Technologies) from DNase-treated (Ambion, TURBO DNA-free) independent total RNA extracts isolated from the same tissues as the three biological replicates used for RNA-Seq. SensiFAST SYBR No-ROX Kit (Bioline) was used in a Rotor-Gene Q real-time PCR cycler (Qiagen) following manufacturer’s protocol with 20 μL reactions containing 20 ng cDNA and 10 μM of each primer. QPCR cycling conditions were: 95°C for 2 min, 40 cycles of 94°C for 5 s, 55°C for 10s, and 72°C for 20 s. Three biological replicates and two technical replicates were used for each sample. Threshold cycle (Ct) number was calculated from log scale amplification curves by Rotor-Gene Q Series software. Relative expression levels of target genes were calculated using 2− (Ct of target−Ct of reference) [27]. For validation, qPCR result was compared with RNA-Seq data and Pearson correlation coefficient R and associated p values were calculated (www.socscistatistics.com).

**Table 1:** List of qPCR primers used in this study.

Primer	Sequence 5' to 3'	RefSeq ID	Predicted maize gene product
HSPFF	CGCCGTTCCTCAGCAAGACG	NP_001149902	Heat shock protein factor 7
HSPFR	CGTAGGTGTTGAGCTGGCGC		
PPF	AACATCGAGGCCAGGCTCCA	NP_001150720	Pathogenesis protein*
PPR	GGCCTCTCCGAACGTTCACC		
TIFF	CAGAGGGAGCAGCAGGAGGA	NP_001152593	Transcription initiation factor TFIID subunit 11
TIFR	AGCTTGTCAGGGTCGCCAGT		
TBIF	GCCTTCGTCGTCAAGGTGCC	NP-001149807	Transmembrane BAX inhibitor motif-containing protein
TBIR	GGCACAGCACGAGGAAAGGC		
PTF	AGGAGATGAAGCAGCCCAGG	NP_001148639	Protein transporter*
PTR	TCCACCTGACGTTGCTCTCG		
UBCPF	CAGGTGGGGTATTCTTGGTG	NP_001148222	Ubiquitin-conjugating enzyme E2-17
UBCPR	ATGTTCGGGTGGAAAACCTT		
CULF	GAAGAGCCGCAAAGTTATGG	XP_008662971	Cullin-1 isoform X1
CULR	ATGGTAGAAGTGGACGCACC		

* Function predicted using BLAST2GO software

## Results and discussion

### Virus infection of maize

**Fig 1:**
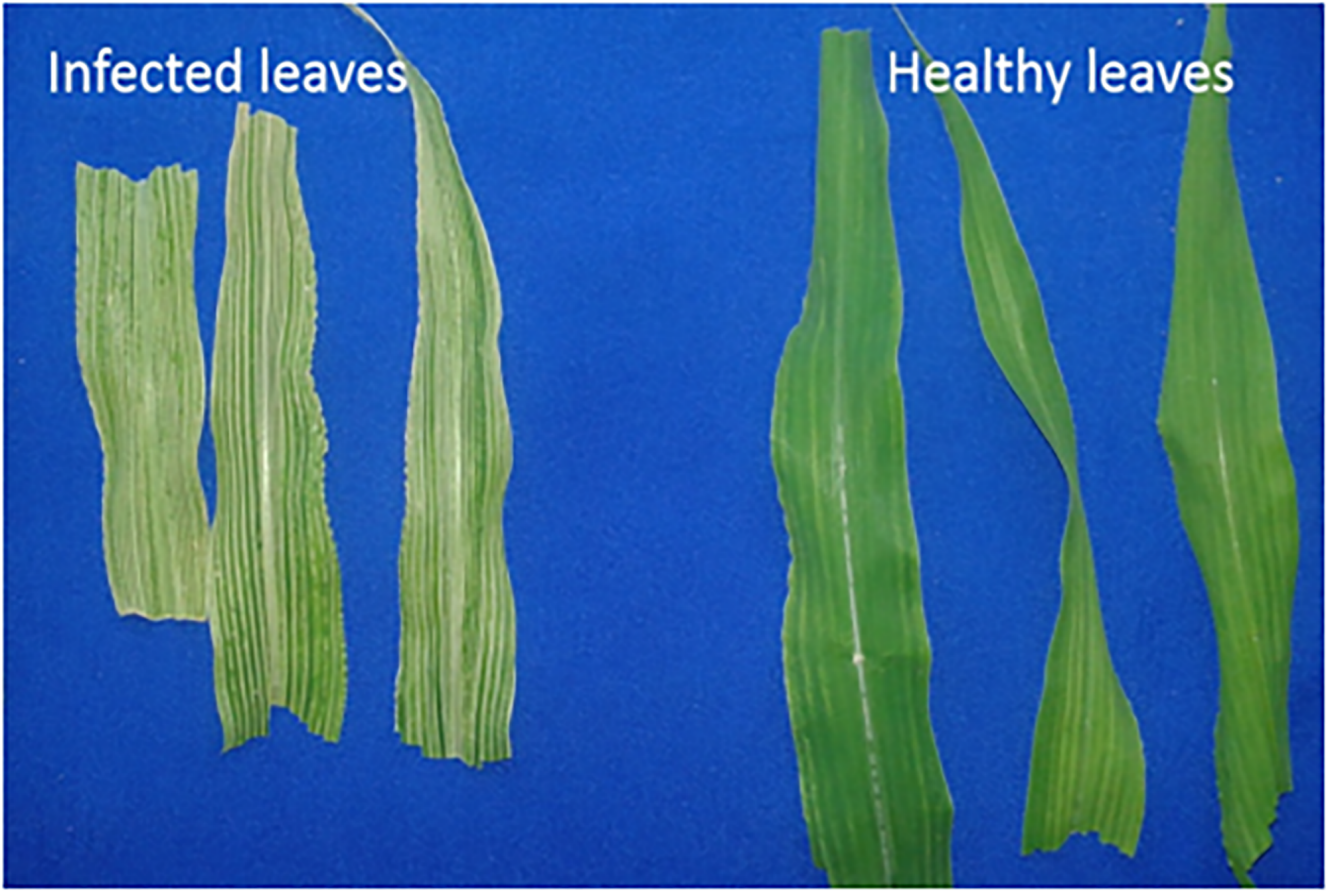
Symptoms on leaves of MIMV-inoculated maize (A) compared with leaves of mock-inoculated plants (B), 15 days after exposure viruliferous (A) or non-viruliferous plant hoppers (B).

Maize plants grown under greenhouse conditions displayed typical MIMV symptoms 15 days after planthopper-mediated infection. MIMV-infected leaves had yellow stripe, chlorosis and green mosaic symptoms (Fig 1) and plants were stunted. The presence of MIMV in infected plants and its absence in mock-inoculated plants was confirmed by DAS-ELISA and RT-PCR.

### Transcriptome sequencing, data processing, and differential gene expression analysis

**Fig 2:**
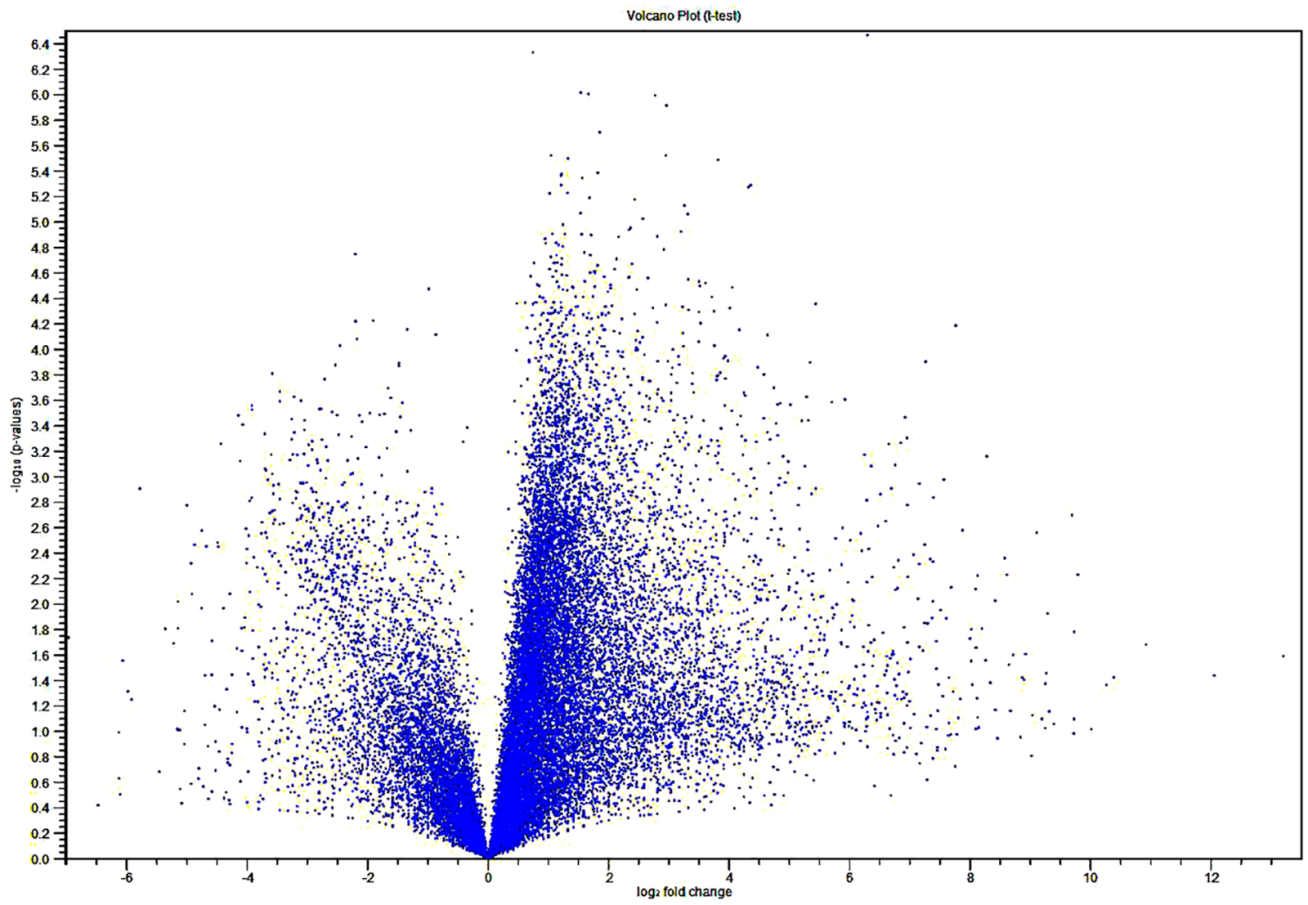
Volcano plot (t-test) showing MIMV-maize RNA-Seq data. X-axis shows fold-change of gene expression and y-axis shows statistical significance (–log10 of the p-value). Down-regulated transcripts are plotted on the left, up-regulated transcripts on the right.

In this study, we have used comparative transcriptome analysis of uninfected and virus-infected maize leaves to investigate maize-MIMV molecular interactions. To obtain a maize gene expression profile, and identify genes and processes responsive to MIMV infection, six cDNA libraries were constructed for deep sequencing using the Illumina High-Seq 2500 platform. After removing adaptors and reads of low-quality nucleotides, we obtained about 40 million clean reads for each library (Table 2) that were mapped to the maize reference genome. The Illumina sequence read data are accessible through the NCBI BioProject database with accessions numbers SAMN08237478 –SAMN08237483. Combination of the sequence data from the six libraries revealed 39,480 maize transcripts larger than 100 bp. Furthermore, comparative transcriptome analysis showed that 1902 transcripts were DE in MIMV-infected compared to uninfected leaves, 1689 transcripts (88.8%) were up-regulated (fold change > 2, p-value <0.05) (S2 Table) and 213 transcripts (11.2%) were down-regulated. This is illustrated in the volcano plot (Fig 2) that shows the distribution of DE reads.

**Table 2:** Raw data statistics of MIMV-infected maize and uninfected maize libraries.

Library	Total Bases^1^	Read Count^2^	GC (%)^3^	AT (%)^4^	Q20 (%)^5^	Q30 (%)^6^
MIMV-infected 1	4,147,751,244	41,066,844	52.22	47.78	97.06	94.99
MIMV-infected 2	4,502,940,772	44,583,572	54.46	45.54	97.90	96.27
MIMV-infected 3	4,501,519,500	44,569,500	52.01	47.99	98.0	96.45
Healthy 1	3,874,450,294	38,360,894	54.84	45.16	97.52	95.68
Healthy 2	4,103,898,054	40,632,654	46.210	53.79	97.243	95.278
Healthy 3	4,500,617,328	43,989,735	52.02	47.98	97.80	96.50

^1^ Total number of bases sequenced.

^2^ Total number of reads in Illumina paired-end sequencing.

^3^ GC content.

^4^ AT content.

^5^ Ratio of reads with phred quality score > 20.

^6^ Ratio of reads with phred quality score > 30.

### RT-qPCR validation of RNA-Seq data

**Fig 3:**
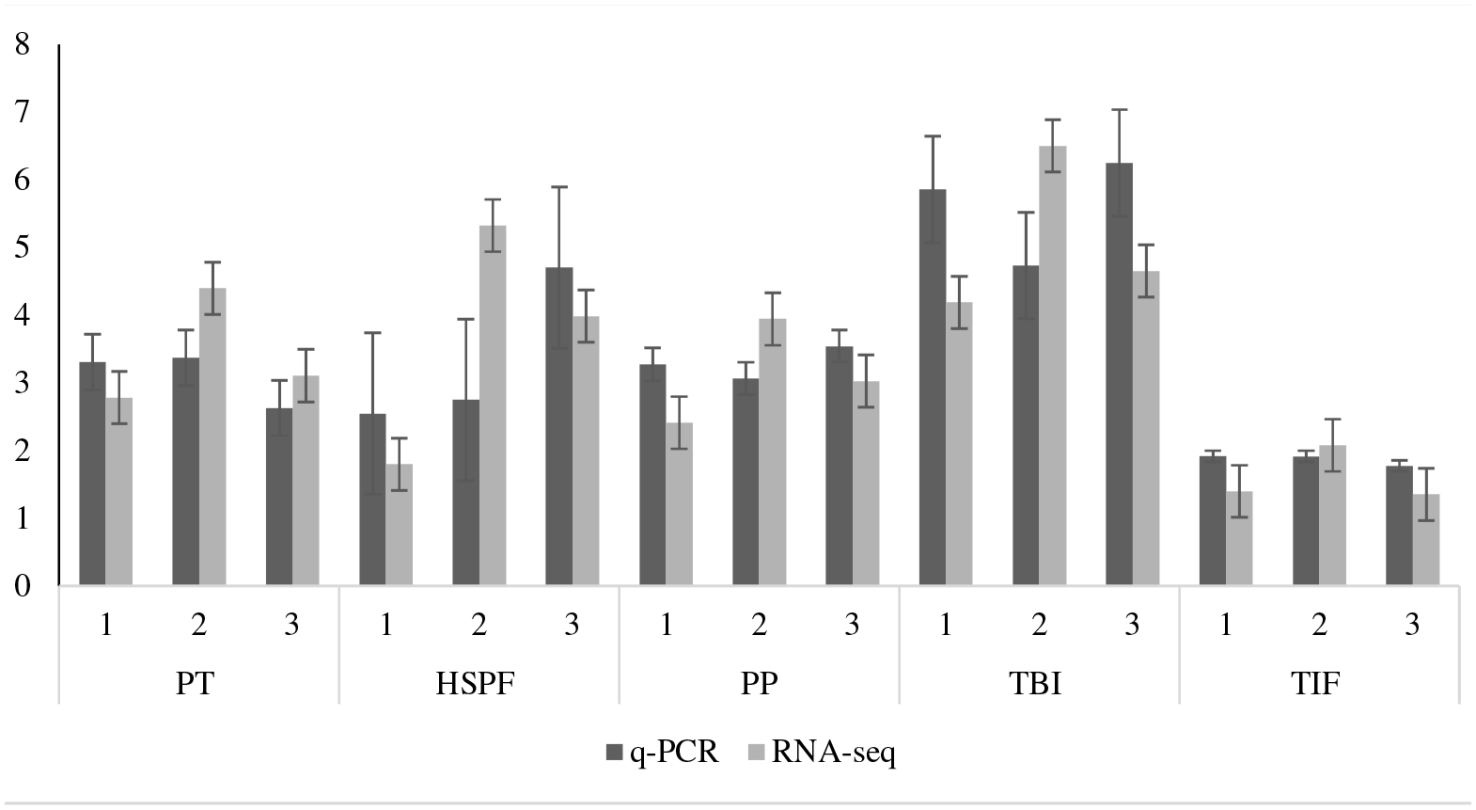
Validation of MIMV responsive up-regulated maize genes. RT-qPCR (Black bars) vs RNA-Seq (Grey bars) fold-change expression of selected genes in three biological replicates (1–3). PT: Protein transporter, HSPF: Heat shock protein factor 7, PP: Pathogenesis protein, TIF: Transcription initiation factor TFIID subunit 11, TBI: Transmembrane BAX inhibitor motif containing protein.

To test the reliability of RNA-Seq DE data, we compared the expression levels of five selected transcripts, heat shock protein factor 7, transcription initiation factor TFIID subunit 11, transmembrane BAX inhibitor motif-containing protein (TMBIM), and transcripts tentatively annotated using Blast2Go or Ensemble database as a ‘pathogenesis protein’ and a ‘protein transporter’ in MIMV-infected and non-infected maize using qPCR (Fig 3). RNA-Seq and qPCR data for all biological replicates correlated well (R = 0.85, p<0.01) suggesting that the DE data are reliable and reflect the actual maize transcriptome differences in response to MIMV infection. Heat shock protein transcript was up-regulated in this study (Fig 3) similar to the findings for other plant RNA and DNA viruses [28–30]. TFIID plays a key role in the regulation of gene expression by RNA polymerase II through transcriptional activation and promoter recognition [31] that may be involved in MIMV gene expression. TMBIM is an anti-apoptotic protein that controls cell death during stress such as virus infection [32] and that was up-regulated in MIMV-infected maize (Fig 3).

### Strongly down- or up-regulated transcripts in response to MIMV infection

**Fig 4:**
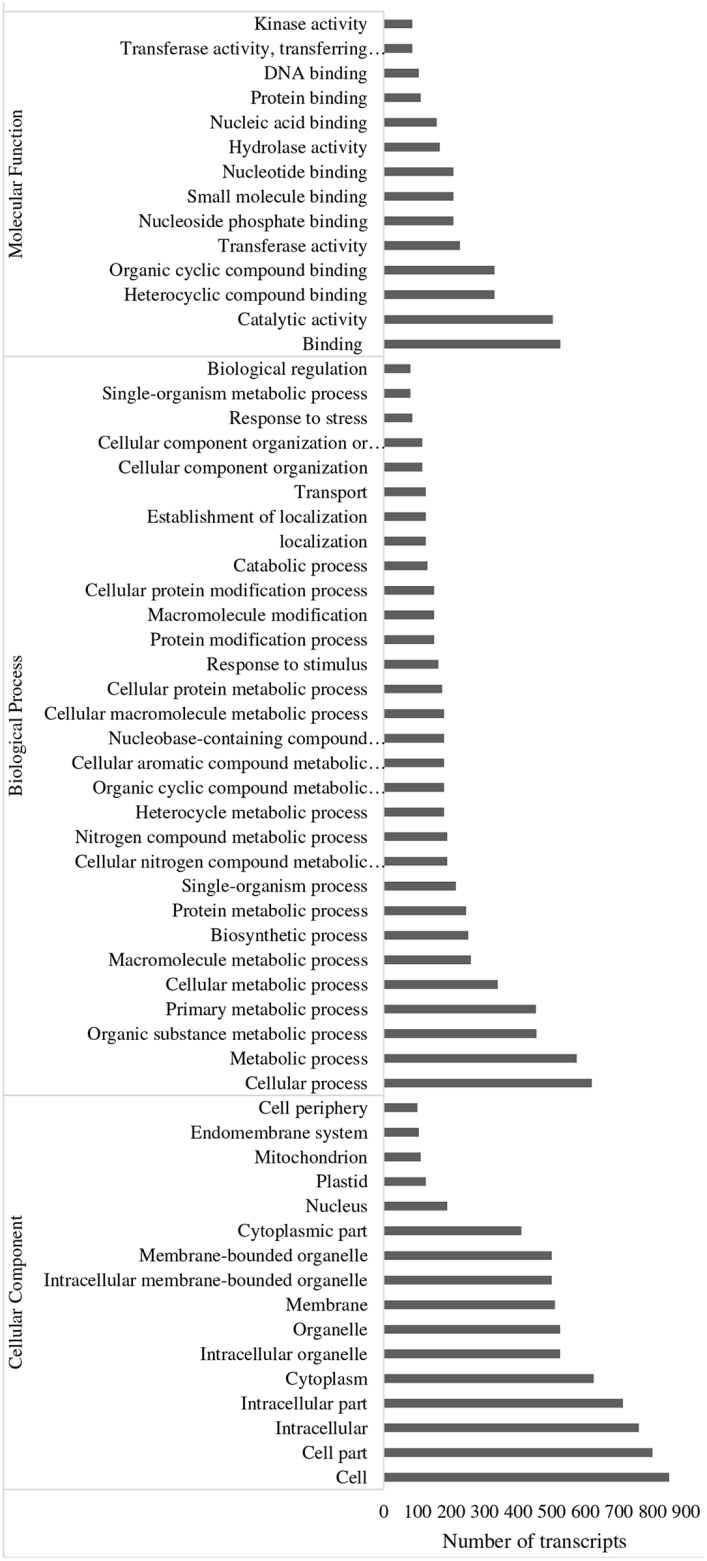
Gene ontology categories of up-regulated transcripts in maize leaves in response to MIMV infection determined using Blast2GO.

**Fig 5:**
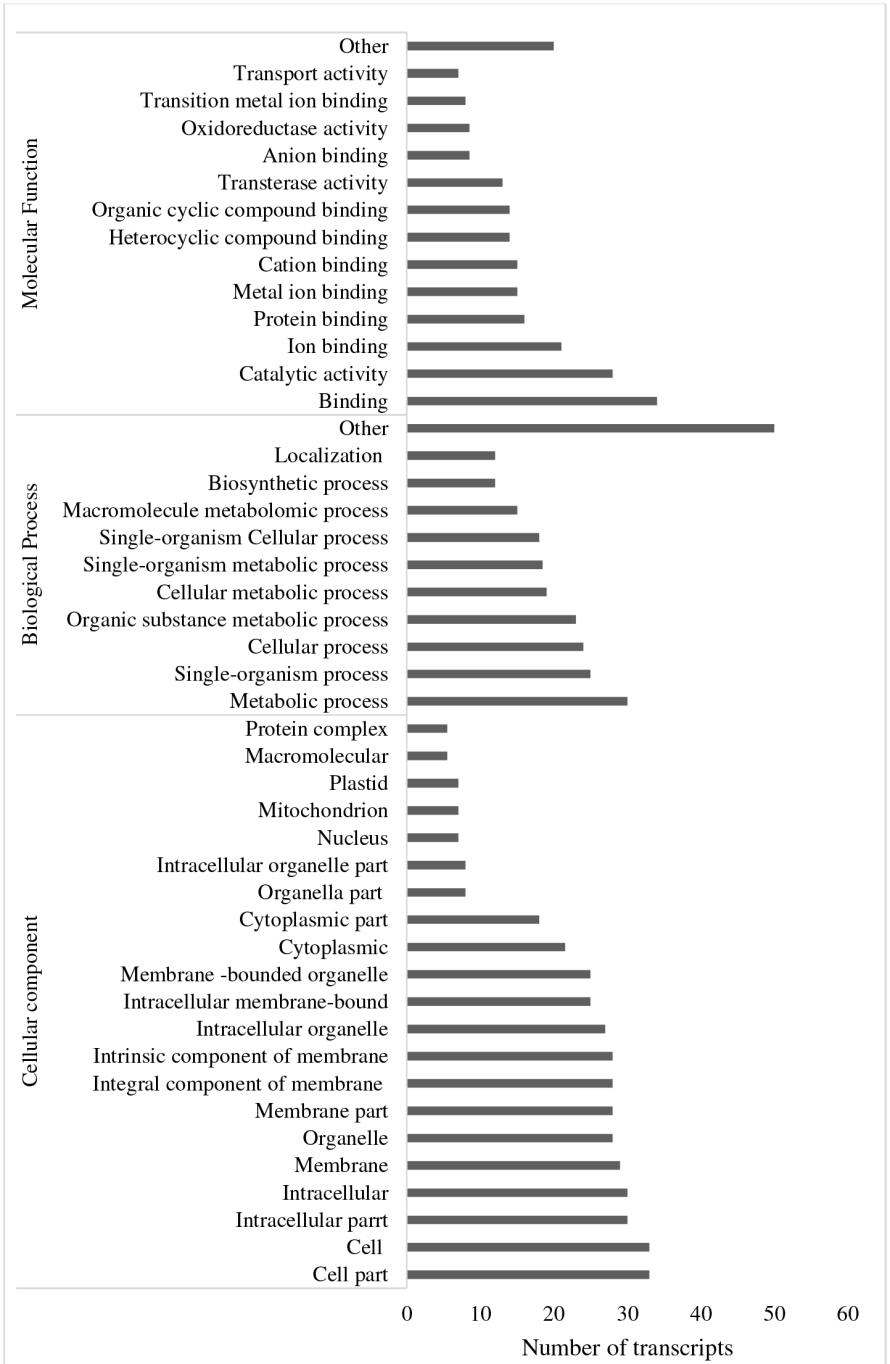
Gene ontology categories of down-regulated transcripts in maize leaves in response to MIMV infection determined using Blast2GO.

Thirteen transcripts were most strongly up-regulated (fold change >80) in MIMV-infected maize leaves (Table 3). ‘Integral component of membrane’ transcripts were significantly affected, both down (28 transcripts) and up (500 transcripts) regulated following MIMV infection (Table 3, Figs 4 and 5). Enveloped viruses like MIMV have been shown to interact with cellular membranes [3] and may affect dynamics of cell and membrane proteins. Transcription factors play roles in gene regulation and are responsive to biotic and abiotic stress [21]. Immune system and plant response to viruses is controlled by transcription factors [23]. We identified fourteen transcription factors (TF) that were significantly up-regulated in this study (Table 3, S1 Table). For example, NAC domain TF transcripts were increased >300-fold in MIMV-infected maize (Table 3). This particular TF was previously reported to interact with the nucleocapsid protein of the nucleorhabdovirus sonchus yellow net virus in *N*. *benthamiana* [33] and was shown to be involved in the regulation of systemic host defenses by interaction with tobacco mosaic virus replicase [34]; it may have a similar role in modulating maize defense responses to MIMV infection. Hydrophobic protein RCI2B, which is induced in plants in response to biotic and abiotic stress [35, 36], was strongly up-regulated in infected maize. Adenosylmethionine decarboxylase, which plays an essential regulatory role in the polyamine biosynthetic pathway [37] was also up-regulated in MIMV-infected maize (Table 3). Constitutive expression of this enzyme was shown to increase tolerance of tomato to stress [36]. Oxidoreductases catalyze the transfer of electrons between molecules and are important in plant defense signaling [38]. Several transcripts that relate to oxido-reductase and -transferase activity were induced in MIMV-infected maize indicating their importance in maize-MIMV interaction.

**Table 3:** Top 13 up-regulated transcripts in MIMV-infected compared to uninfected maize.

Transcript No.	GenBank accession	Fold Change	FDR p-value	Predicted gene function
1	NP_001140915	82	0.02862761	Metal ion binding, oxidoreductase activity
2	NP_001130429	91.2	0.03610904	Oxidoreductase, aldo/keto reductase family protein
3	NP_001149266	100.6	0.024657796	S-adenosylmethionine decarboxylase
4	NP_001105511	103.5	0.034602873	Glutathione transferase 35
5	NP_001147075	124.1	0.02357992	Nucleotide binding protein
6	XP_008657926	124.5	0.039400598	Zinc finger protein ZAT8-like
7	NP_001141884	143	0.033178243	Transcription factor DIVARICATA-like
8	NP_001153094	153.6	0.015501405	Metal-binding and oxidoreductase
9	LOC100280292	168	0.037108401	Flavin adenine dinucleotide binding
10	NP_001148452	234.8	0.046469706	Nucleic acid binding and endonuclease activity
11	NP_001147618	310.4	0.026666236	NAC domain transcription factor
12	EU955582	552	0.047195478	Hydrophobic protein RCI2B
13	XP_008652274	828	0.041989032	Unknown protein, integral component of membrane*

* Function predicted using BLAST2GO software

Pattern-triggered immunity (PTI) and effector-triggered immunity (ETI) are a two-part plant immune response to pathogens [39]. ETI results from detection of pathogen effectors by resistance proteins and PTI occurs following recognition of microbe- or pathogen associated molecular patterns. Both PTI and ETI are involved in early signaling events, including changes in protein phosphorylation status [39]. Some protein kinase and ATP binding activity-related transcripts were highly down-regulated in MIMV-infected maize (Table 4) whereas some others were up-regulated as previously shown in tomato cultivars resistant to tomato chlorotic mottle virus [40]. Differential expression of those host genes appears to depend on the type of transcripts and their specific roles in cell signaling. The majority of down-regulated transcripts with fold change less than 15 encoded uncharacterized proteins (Table 4).

**Table 4:** Top 13 down-regulated transcripts in MIMV-infected compared to uninfected maize.

Transcript No.	GenBank accession	Fold Change	FDR p-value	Predicted gene function
1	GRMZM2G113883*	-55	0.03463707	Uncharacterized protein
2	XP_008659627	-32	0.03957	Uncharacterized protein
3	GRMZM2G314339*	-27	0.04646	Uncharacterized protein
4	XP_020402672	-21.6	0.024615	Uncharacterized protein
5	NP_001152364	-17.7	0.02069	Protein kinase activity and ATP binding**
6	GRMZM2G064008*	-17.35	0.02772	Uncharacterized protein
7	GRMZM2G064250*	-16.8	0.02145	Integral component of membrane**
8	NP_001131699	-16.2	0.045915	Uncharacterized protein
9	XP_020407456	-15.7	0.0483	Uncharacterized protein
10	AC217841.3_FG001*	-15.5	0.03820	Uncharacterized protein
11	AQK91281	-15.2	0.03725	Uncharacterized protein
12	XP_008660379	-15.14	0.02004	Calcium uniporter protein
13	AC193658.3_FG001*	-15.1	0.02037	Uncharacterized protein

* Ensembl Plants ID (without any information in NCBI)

** Gene Ontology feature

### Functional annotation

MIMV-responsive maize transcripts were classified by gene ontology (GO) categories using Blast2Go. In the molecular function category, catalytic activity and various binding activities were both up- and down-regulated, but more transcripts in this category were up- than down-regulated (Figs 4 and 5). On the other hand, organic cyclic compound binding, transferase activity enzymes, kinases, hydrolase activity, and nucleotide binding related transcripts were significantly enriched in MIMV-infected maize tissue (Fig 4).

The important role of kinases in plant innate immunity is well known [41] and the response of maize to MIMV infection is no exception. GO classification of up-regulated transcripts in molecular function showed 158 transcripts that relate to nucleotide binding, 167 to hydrolase activity and 330 to heterocyclic compound binding that are well known responses in plant defense [40, 42]. Transcripts that relate to nucleotide binding were also up-regulated in maize infected with maize chlorotic dwarf virus (MCDV) and the nucleorhabdovirus maize fine streak virus (MFSV) [19]. Most of the up-regulated transcripts in MIMV-infected maize fell into the biological categories of cellular and metabolic processes (Fig 4). Transcripts related to stress response, like hormone signaling and synthesis of secondary metabolites were induced in MIMV-infected maize, similar to capsicum response to tospovirus infection [8]. Geminivirus infection in tomato led to up-regulated transcripts involved in cell wall reorganization, transcriptional regulation and secondary metabolite synthesis [43] that were similarly up-regulated in this study. Transcripts involved in metabolic and single-organism process categories were up- or down-regulated in this study, but the number of transcripts that were up-regulated were about 19 times and 8 times higher, respectively than that of down-regulated transcripts in those categories (Figs 4 and 5). Viruses rely on host cell transport systems and have been shown to affect expression of transport-related genes [29]; several responsive transcripts in this study were also involved in transport (Figs 4 and 5). GO analysis of cellular components showed up-regulated transcripts associated with all parts of the cell (Fig 4).

Transcripts for several transcription factors including MYB and NAC were up-regulated in MIMV-infected plants (S1 Table). On the other hand, transcripts for the transcription factors ASIL1-like, ICE1 and transcription repressor OFP13 were down-regulated. Leucine-rich repeat (LRR) receptor-like serine threonine-kinase that belongs to the nucleotide-binding (NB) LRR gene family (S1 Table), and represents the majority of known disease resistance genes in plants [44], was down-regulated in MIMV-infected maize possibly due to the susceptibility of this maize cultivar to MIMV. ‘Constitutive expressor of pathogenesis’ related transcripts were also up-regulated during MIMV infection (S1 Table); this gene is involved in cell wall biogenesis, cell signaling or transcription [45]. Our data show significant down- or up-regulation of transcripts related to defense response, plant-pathogen interaction and response to biotic and abiotic stress, as well as nucleic acid binding during MIMV infection in maize.

### MIMV-responsive biochemical pathways

KEGG analysis showed that 58 different pathways were up-regulated in response to MIMV infection in maize. The top five pathways that contained the largest numbers of up-regulated genes in response to MIMV were metabolic pathways (100 transcripts), biosynthesis of secondary metabolites (51 transcripts), proteasome (30 transcripts), protein processing in endoplasmic reticulum (28 transcripts) and endocytosis (21 transcripts) indicating that MIMV replication leads to significant metabolic changes in infected tissues. Fatty acid metabolism (9 transcripts) and fatty acid degradation (9 transcripts) are two pathways induced by MIMV that were shown previously to play significant roles in pathogen defense [46].

Viruses have been shown to disrupt many processes in plant cells, resulting in temporal changes that affect hormone signaling and metabolite responses [47]. Plant hormones play important roles in complex plant response networks and tune plant responses to biotic and abiotic stress [48]. Four hormones primarily regulate plant defense to pathogens: salicylic acid, jasmonic acid, ethylene and abscisic acid [48]. Eighteen transcripts were up-regulated following MIMV infection that GO analysis showed are involved in plant hormone signal transduction, similar to maize infected by *Bipolaris zeicola* and *Fusarium graminearum* [16, 17]. Gibberellin 20 oxidase was up-regulated in this study (S1 Table) which may be related to the observed stunted growth of maize infected with MIMV. Plant-pathogen interaction pathways (13 transcripts) that are related to hormone signaling and plant immunity were up-regulated in MIMV-infected maize, similar to maize infected by *B*. *zeicola* [16]. Nine transcripts were up-regulated in MIMV-infected maize plants that are related to RNA transport pathways (S1 Table) linked to the well-known nucleo-cytoplasmic trafficking activities in maize plants [49]. In this study, five transcripts were up-regulated in MIMV-infected plant that are involved in RNA degradation with functions in messenger RNA biogenesis, mRNA-decapping and spliceosome activities (S1 Table). We identified 35 photosynthesis-related transcripts that were responsive to MIMV infection (S1 Table) and that may be related to yellow stripe and chlorosis symptoms on the leaves (Fig 1).

#### Metabolic pathways and biosynthesis of secondary metabolites

**Fig 6:**
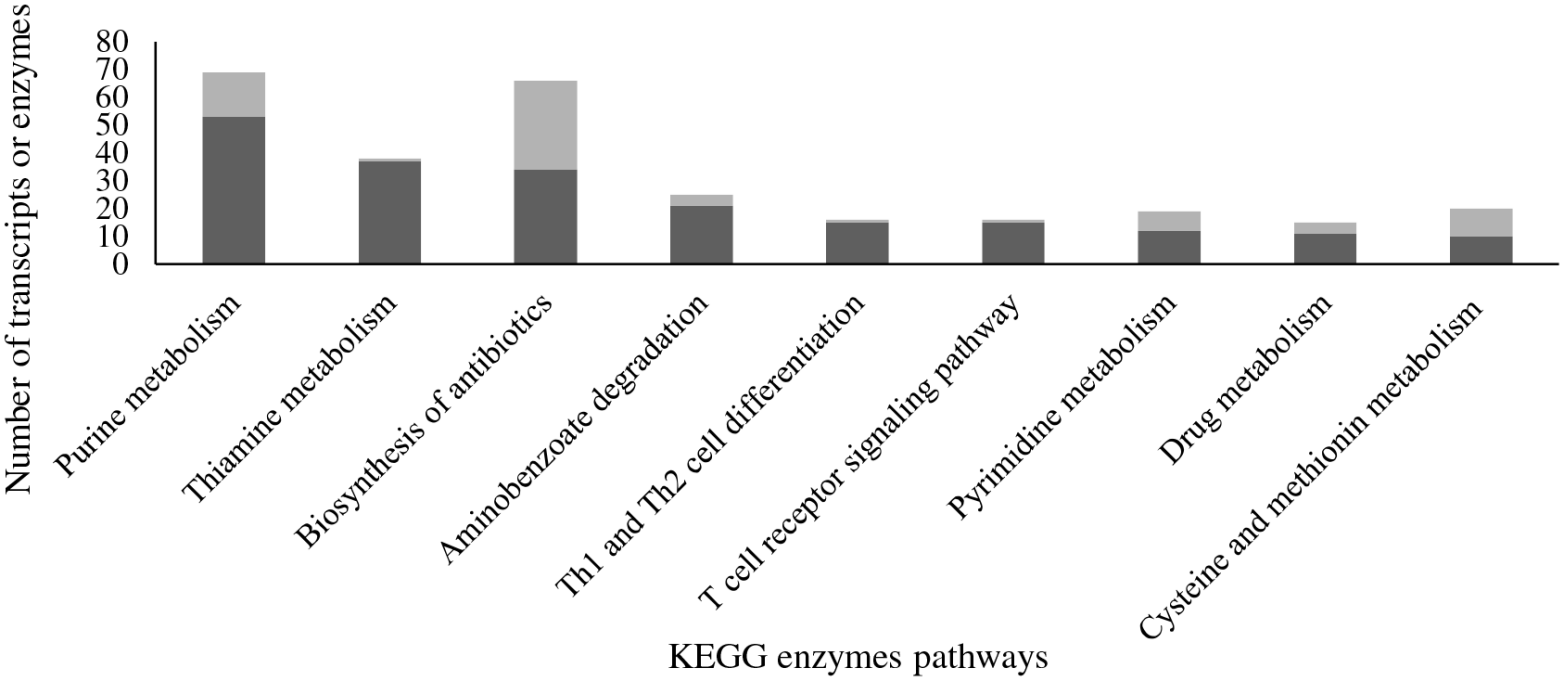
KEGG pathways for up-regulated enzymes determined using BLAST2GO. Black color shows the number of transcripts involved in each process and gray color shows the number of enzymes involved.

Plants produce chemically diverse secondary metabolites and every plant species has its own characteristic set [50]. In our study, most of the maize transcripts related to metabolic processes were up-regulated in response to MIMV infection similar to what has been reported following virus infection in rice, jatropha, capsicum and tobacco [8, 21, 51, 52] and also similar to maize infected by *B*. *zeicola* [16]. Several transcripts involved in the biosynthesis of secondary metabolites such as antibiotics were up-regulated in this and other studies upon virus infection (50). Mapping up-regulated transcripts for secondary metabolite enzymes into KEGG pathways revealed genes involved in the biosynthesis of antibiotics (34 transcripts), biosynthesis of tyrosine (6 transcripts), tryptophan biosynthesis (7 transcripts), phenylalanine metabolism (5 transcripts), and terpenoid backbone synthesis pathway (7 transcripts) (Fig 6), which lead to the synthesis of antimicrobial phytoalexins and phytoanticipins, and phenolic compounds that are known to be involved in plant defense against pathogens [53].

#### Protein processing in the endoplasmic reticulum

Twenty-eight maize transcripts related to protein processing in the endoplasmic reticulum (ER) were up-regulated upon MIMV infection (S1 Table). These included transcripts for ubiquitin-conjugating enzymes, chaperone DNA, heat shock proteins, protein disulfide enzyme, N-Glycan biosynthesis, a transport protein, calnexin, B-cell receptor-associated proteins and Derlin proteins (transmembrane proteins that detect and target misfolded proteins in the ER). This responsiveness may be related to MIMV surface glycoprotein G or matrix protein M interacting with the ER during virus maturation [3]. Previous studies have shown that G protein of the animal rhabdovirus vesicular stomatitis virus is incorporated into ER membranes where chaperones like calnexin facilitate its proper folding and assembly into trimmers and its posttranslational glycosylation [54].

#### Ubiquitin-mediated proteolysis and proteasome

Ubiquitin-mediated proteasome pathway targets proteins for degradation by the 26S proteasome and has been implicated in a number of plant diseases, especially virus infections [48, 55, 56]. Polerovirus P0 proteins and beet necrotic yellow vein virus p25 and p31 proteins have been shown to induce ubiquitination in host cells [9, 55, 56]. MIMV infection of maize led to up-regulation of transcripts for proteins in the ubiquitin-proteasome pathways such as ubiquitin-conjugating enzymes, ubiquitin-protein ligase, proteasome maturation protein, 20S proteasome and 26S proteasome consistent with a previous study on maize infected with MCDV and MFSV [19]. Our data provide evidence that ubiquitin also has an important role in response to MIMV infection.

#### KEGG enzyme pathways

A total of 110 KEGG enzyme pathways were up-regulated in response to MIMV infection in maize (S3 Table). Largest number of up-regulated transcripts mapped to purine metabolism (53 transcripts, 16 enzymes), thiamine metabolism (37 transcripts, 1 enzyme), biosynthesis of antibiotics (34 transcripts, 32 enzymes), aminobenzoate degradation (21 transcripts, 4 enzymes) and Th1 (T-helper 1) and Th2 (T-helper 2) cell differentiation (15 transcripts, 1 enzyme). Th1 and Th2 are involved in immune regulation of animal cells against pathogens [57, 58]. This analysis also showed that transcripts involved in synthesis of amino acids were significantly induced in response to MIMV infection. Transcripts involved in proline metabolism were up-regulated in MIMV-infected maize in this study, in *Arabidopsis thaliana* after infection with tobacco etch virus and in cotton in response to sap sucking insects [59, 60]. Biosynthesis of vitamins was also induced in response to MIMV infection as evidenced by the up-regulation of 37 transcripts that are involved in the biosynthesis of thiamine, a plant defense signaling molecule [61].

MIMV replicates in the nucleus of infected maize cells and establishes a nuclear viroplasm [6]. KEGG pathway analysis of our RNA-Seq data showed that of 141 nucleus-associated transcripts, four were related to plant-pathogen interactions, two to vesicular transport and three to RNA transport (S3 Table). These nuclear transcripts were upregulated in response to MIMV infection and may be considered as transcripts encoding MIMV replication-associated host factors.

## Conclusions

In response to MIMV infection, maize plants activated multiple pathways, including immune receptor signaling, metabolic pathways, RNA silencing, hormone-mediated pathways, protein degradation, protein kinase and ATP binding activity, and fatty acid metabolism. Many of these pathways are known to be involved in plant defense. Transcripts that were significantly up-regulated in this study are reportedly involved in plant response to pathogens in other pathosystems, pointing to common responses across plant species and pathogens. Several transcripts lacking annotations were up and down-expressed with high fold change in response to MIMV-infection. These unknown genes will be explored in future studies to better understand the infection mechanism of MIMV in maize.

## Supporting information

S1 Table List of maize transcripts characterized by gene ontology terms. (DOCX)

S2 Table Data output from CLC Genomics Workbench software. RNA-Seq dataset of responsive transcripts in MIMV-infected maize compared to uninfected maize. Column A is the Feature ID or Ensemble ID of transcripts, column B is the fold change >2 of transcripts, column C is the p-value, columns D-F are Fragments Per Kilobase Million (FPKM) of uninfected samples, column G is the mean of FPKM in uninfected samples, columns H-J are FPKM of infected samples and columns K is mean of FPKM in infected samples.
(XLSX)

S3 Table Maize up-regulated transcripts in MIMV-infected compared to uninfected maize attributed to the Kyoto Encyclopedia of Genes and Genomes (KEGG) pathways. Transcripts are shown with Ensemble ID. Columns A-BI show the name of KEGG pathways and the transcripts that are involved in those pathways.
(XLSX)
