# Supplementary material for: Changes in maize transcriptome in response to maize Iranian mosaic virus infection
Source: PLoS One. 2018 Apr 10;13(4):e0194592. doi: 10.1371/journal.pone.0194592 (PMC5892904; doi:10.1371/journal.pone.0194592)
Supplement: S1 Table — (DOCX) [file pone.0194592.s001.docx]

| S1 Table. List of maize transcripts characterized by Gene Ontology terms | | | |
| --- | --- | --- | --- |
| Transcript name | **Gene description ^a^** | **GO terms ^b^** | |
| GRMZM2G017685_P01 | Hypothetical protein ZEAMMB73_484654 | C: membrane | |
| nac126 | NAC domain-containing 77 | F: DNA binding; P: biosynthetic process, nucleobase-containing compound metabolic process; C: nucleus | |
| mybr37 | Transcription factor DIVARICATA | F: DNA binding; P: biosynthetic process, nucleobase-containing compound metabolic process; C: nucleus | |
| pco102923 | Nuclease S1 | F: nucleic acid binding, nuclease activity; P: catabolic process; DNA metabolic process; C: cytoplasm | |
| aasr6 | Abscisic stress-ripening 3-like | P: response to stress | |
| GRMZM2G086210_P01 | HOTHEAD | F: nucleotide binding, catalytic activity; P: metabolic process; C: cytoplasm | |
| GRMZM2G112799_P01 | Zinc finger ZAT11-like | F: transcription factor activity, sequence-specific DNA binding; P: biosynthetic process, nucleobase-containing compound, metabolic process; C: nucleus, plastid | |
| GRMZM2G132875_P02 | Aldo keto reductase family | F: catalytic activity; P: metabolic process; C: membrane | |
| fls1 | Flavonol synthase | F: catalytic activity, binding; P: biosynthetic process | |
| gst35 | Glutathione S-transferase GSTU6 | F: transferase activity; P: secondary metabolic process, catabolic process, cellular process; C: cytoplasm | |
| pmpm4 | Low temperature-induced | C: cytoplasm, membrane | |
| GRMZM2G317614_P01 | Nucleotide binding |  | |
| GRMZM2G333095_P01 | Cell wall RBR3-like | F: oxidoreductase activity; P: DNA repair, oxidation-reduction process | |
| GRMZM2G335407_P01 | GPI-anchored -like | C: cytoplasm, membrane | |
| GRMZM2G376061_P01 | Zinc finger ZAT11-like | F: transcription factor activity, sequence-specific DNA binding; P: biosynthetic process, nucleobase-containing compound metabolic process; C: nucleus | |
| nac111 | NAC domain-containing 8-like | F: DNA binding; P: biosynthetic process, nucleobase-containing compound metabolic process; C: nucleus | |
| IDP754 | S-adenosylmethionine decarboxylase proenzyme-like | F: catalytic activity; P: biosynthetic process, nucleobase-containing compound metabolic process; C: cytosol | |
| GRMZM5G817559_P01 | Acyltransferase, chloroplastic | F: transferase activity; P: lipid metabolic process, biosynthetic process, cellular process; C: plastid | |
| CYP734A7 | Cytochrome P450 monooxygenase | F: catalytic activity, binding; P: lipid metabolic process, growth; C: membrane | |
| GRMZM5G868296_P01 | Unknown |  | |
| cl51135_1 | Calcium-dependent kinase 8-like | F: nucleotide binding, protein binding, kinase activity; P: signal transduction, response to endogenous stimulus, cellular protein modification process; C: nucleus, cytoplasm, plasma membrane | |
| cl36099_1a | SH3 domain-containing 2-like | C: cytosol | |
| GRMZM2G104179_P01 | Tetratricopeptide repeat 1 | F: protein binding; C: plastid | |
| GRMZM2G104226_P01 | GDSL esterase lipase At1g28600-like isoform X1 | F: hydrolase activity; C: membrane | |
| GRMZM2G104299_P01 | AT-hook motif nuclear-localized 10 | F: DNA binding | |
| GRMZM2G104294_P01 | NADPH--cytochrome P450 reductase-like | F: nucleotide binding, catalytic activity; P: metabolic process; C: membrane, endoplasmic reticulum | |
| GRMZM2G104339_P01 | TPA: hypothetical protein  ZEAMMB73_008333 | | |
| GRMZM2G104504_P01 | Pantothenate kinase 2 | F: nucleotide binding, kinase activity; P: biosynthetic process, nucleobase-containing compound metabolic process | |
| cl3444_1 | Eukaryotic translation initiation factor 3 subunit 3 | F: translation factor activity, RNA binding; P: response to abiotic stimulus, post-embryonic development, cellular component organization, cellular protein modification process; C: cytosol, ribosome | |
| GRMZM2G104676_P01 | Cell division control | F: hydrolase activity; P: transport, cellular process; C: endosome, cytoplasm, membrane, Golgi apparatus | |
| GRMZM2G104955_P01 | PAT1 homolog 1-like isoform X1 | F: RNA binding, transferase activity; P: catabolic process, lipid metabolic process, cellular component organization, biosynthetic process, nucleobase-containing compound metabolic process; C: cytoplasm, membrane | |
| NRH3 | Uridine nucleosidase 2 | F: hydrolase activity, binding; P: catabolic process, nucleobase-containing compound metabolic process; C: cytosol | |
| umc1149 | Glutathionyl-hydroquinone reductase -like | F: transferase activity | |
| GRMZM2G105401_P01 | WD repeat-containing 55 | P: embryo development, post-embryonic development, cellular component organization, cell differentiation, biosynthetic process, nucleobase-containing compound metabolic process, reproduction; C: nucleus, cytoplasm | |
| GRMZM2G105539_P01 | Arsenical pump-driving ATPase | F: nucleotide binding, transporter activity, hydrolase activity; C: mitochondria | |
| hb62 | Homeobox-leucine zipper HOX19 | F: DNA binding, transcription factor activity, sequence-specific DNA binding; P: biosynthetic process, nucleobase-containing compound metabolic process; C: nucleus | |
| pco071587 | Hypothetical protein | C: mitochondria | |
| GRMZM2G105999_P01 | E3 ubiquitin- ligase RNF4-like | F: binding, transferase activity; P: catabolic process, cellular protein modification process; C: nucleus | |
| GRMZM2G106283_P01 | Transport yos1 | C: membrane, cytosol | |
| GRMZM2G106552_P01 | Branched-chain amino acid | F: transferase activity; P: metabolic process, cellular process | |
| GRMZM2G106960_P01 | Ras-related RIC1 | F: nucleotide binding; P: signal transduction, transport; C: plasma membrane, endoplasmic reticulum, Golgi apparatus | |
| pdlk1 | TPA: pyruvate dehydrogenase kinase family | F: nucleotide binding, receptor activity, kinase activity, signal transducer activity; C: cellular component | |
| TIDP2690 | 26S protease regulatory subunit 8 homolog A | F: nucleotide binding, protein binding, hydrolase activity; P: protein metabolic process, catabolic process, cellular component organization, biosynthetic process, nucleobase-containing compound metabolic process, response to stress; C: nucleus, cytosol | |
| GRMZM2G107575_P01 | Calcineurin B 1 | F: binding; C: membrane, vacuole | |
| tac905.27 | PTI1-like tyrosine- kinase At3g15890 | F: nucleotide binding, kinase activity; P: cellular protein modification process | |
| GRMZM2G104836_P01 | Glycine proline-rich family |  | |
| LSM7-like | sm LSM7 | F: RNA binding; P: nucleobase-containing compound metabolic process; C: nucleus, cytoplasm | |
| GRMZM2G106479_P01 | Uncharacterized membrane | C: membrane | |
| GRMZM2G106574_P01 | Splicing factor 3B subunit 5 | P: nucleobase-containing compound metabolic process; C: nucleus | |
| GRMZM2G106690_P01 | USP family | P: response to stress; C: plastid | |
| GRMZM2G106795_P01 | ADP-ribosylation factor | F: nucleotide binding, hydrolase activity; P: signal transduction; C: intracellular | |
| pco137813 | Transmembrane 205-like | C: membrane, endoplasmic reticulum | |
| GRMZM2G107591_P02 | Uncharacterized protein LOC103652906 isoform X4 | C: cytoplasmic vesicle | |
| GRMZM2G107757_P01 | Vacuolar sorting-associated 32 homolog 2-like | P: transport; C: intracellular | |
| GRMZM2G107838_P01 | Lipid phosphate phosphatase, chloroplastic | F: hydrolase activity; P: catabolic process, lipid metabolic process, cellular protein modification process, biosynthetic process, pollination; C: plastid, membrane, endoplasmic reticulum | |
| GRMZM2G108076_P01 | Methylmalonate-semialdehyde dehydrogenase [acylating], mitochondrial | F: catalytic activity, binding; P: carbohydrate metabolic process, generation of precursor metabolites and energy, catabolic process, lipid metabolic process, biosynthetic process, nucleobase-containing compound metabolic process, response to stress; C: plastid, mitochondria, membrane | |
| GRMZM2G108115_P01 | Endoplasmic reticulum oxidoreductin-1 | F: catalytic activity; P: protein metabolic process, response to abiotic stimulus, response to stress; C: membrane, endoplasmic reticulum, plastid | |
| GRMZM2G108265_P03 | Nodulation H-like | F: transferase activity; P: biosynthetic process, nucleobase-containing compound metabolic process; C: nucleoplasm, cytoplasm, membrane | |
| umc2775 | Phosphatase 2C 28 | F: binding, hydrolase activity; P: cellular protein modification process; C: cell, membrane | |
| pco078702 | S-acyltransferase 17 | F: binding, transferase activity; P: cellular process, biosynthetic process; C: cytoplasm, membrane | |
| A9009 | TPA: FYVE zinc finger containing actin-binding-domain family | F: lipid binding; P: cellular component organization; C: plasma membrane | |
| GRMZM2G108775_P01 | tRNA- dihydrouridine(16 17) synthase [NAD(P)(+)]-like | F: nucleotide binding, catalytic activity; P: nucleobase-containing compound metabolic process; C: mitochondria, cytosol | |
| PRO4 | Profilin | F: protein binding; P: cellular component organization; C: plastid, cytoskeleton | |
| GRMZM2G109268_P01 | Plant-specific domain TIGR01589 family | F: hydrolase activity, acting on ester bonds | |
| nlp7 | NLP1-like isoform X1 | F: DNA binding; P: biosynthetic process, nucleobase-containing compound metabolic process; C: nucleus | |
| GRMZM2G109582_P01 | ELC-like | P: cellular protein modification process, transport | |
| nac118 | NAC transcription factor 29-like | F: DNA binding; P: flower development, biosynthetic process, fruit ripening, nucleobase-containing compound metabolic process, cell growth; C: nucleus | |
| GRMZM2G109731_P01 | Phosphatase 1 regulatory subunit pprA |  | |
| GRMZM2G109831_P01 | Uncharacterized protein LOC100191166 | C: membrane, integral component of membrane | |
| GRMZM2G110185_P02 | 26S protease regulatory subunit 7 | F: nucleotide binding, protein binding, hydrolase activity; P: protein metabolic process, catabolic process, cellular component organization, biosynthetic process, nucleobase-containing compound metabolic process, response to stress; C: plasma membrane, cytosol, nucleus | |
| AY109678 | Caffeoyl shikimate esterase | F: hydrolase activity; P: catabolic process, lipid metabolic process; C: plastid, membrane | |
| IBP2 | Telomere repeat-binding 5-like | F: DNA binding; P: cellular component organization, DNA metabolic process; C: nucleus | |
| pza02402 | Magnesium transporter NIPA4 | F: nucleotide binding, kinase activity, transporter activity; P: cellular protein modification process; C: membrane | |
| GRMZM2G110413_P01 | TPA: hypothetical protein  ZEAMMB73_480915 | | |
| GRMZM2G110423_P01 | E3 ubiquitin- ligase ARI1 | F: protein binding, transferase activity; P: catabolic process, cellular protein modification process; C: cytoplasm | |
| PIS | Phosphatidylinositol synthase 2 isoform X1 | F: transferase activity; P: lipid metabolic process, biosynthetic process, cellular process; C: cytoplasm, membrane, Golgi apparatus | |
| lbd3 | LOB domain-containing 18-like | P: post-embryonic development, anatomical structure morphogenesis; C: nucleus | |
| GRMZM2G110922_P01 | Serine threonine- kinase SAPK4 | F: nucleotide binding, kinase activity; P: signal transduction, response to endogenous stimulus, cellular protein modification process; C: nucleus, cytoplasm | |
| pco096181 | Ubiquitin-conjugating enzyme E2 2 | F: nucleotide binding, protein binding, transferase activity; P: catabolic process, cellular protein modification process, cellular component organization, DNA metabolic process, response to stress; C: cytoplasm | |
| NIT2 | Bifunctional nitrilase nitrile hydratase NIT4 | F: hydrolase activity; P: metabolic process | |
| GRMZM2G111247_P01 | WD repeat-containing 26 |  | |
| GRMZM2G111472_P01 | coenzyme Q-binding COQ10 mitochondrial | C: mitochondria | |
| prc2 | Proteasome subunit beta type-2-A | F: hydrolase activity; P: protein metabolic process, catabolic process, cellular process; C: nucleus, cytoplasm | |
| gsh1 | Gamma-glutamylcysteine synthetase | F: catalytic activity, nucleic acid binding; P: secondary metabolic process, biosynthetic process, response to stress, response to biotic stimulus, response to abiotic stimulus, response to external stimulus, flower development, cellular component organization; C: plastid, mitochondria | |
| GRMZM2G111611_P01 | Vesicle transport v-SNARE 13 | F: protein binding; P: cellular component organization, transport; C: cytosol, endoplasmic reticulum, membrane, Golgi apparatus, vacuole, endosome | |
| GRMZM2G112100_P01 | Subtilisin-like protease | F: hydrolase activity; P: protein metabolic process; C: cytoplasm, cell wall | |
| TIDP2694 | Pollen-specific SF21-like | C: membrane | |
| CBL3 | Calcineurin B 3 | F: protein binding; C: membrane, vacuole | |
| IDP1604 | Clathrin light chain 2-like | F: protein binding, structural molecule activity; P: transport; C: plasma membrane, cytoplasm, Golgi apparatus | |
| GRMZM2G113062_P03 | Methionine aminopeptidase 1A | F: binding, hydrolase activity; P: cellular protein modification process; C: cytosol, ribosome | |
| GRMZM2G113250_P01 | Elongation factor 2 | F: nucleotide binding, translation factor activity, RNA binding, hydrolase activity; C: ribosome | |
| GRMZM2G113423_P01 | Muscle M-line assembly unc-89 | F: protein binding | |
| cl37059_1 | EH domain-containing 1 | F: nucleotide binding | |
| uaz207 | LOC100284144 isoform X1 | F: binding; C: membrane | |
| glk38 | Myb family transcription factor-related | F: DNA binding; P: biosynthetic process, P: nucleobase-containing compound metabolic process; C: nucleus | |
| GRMZM2G113995_P01 | ADP-ribosylation factor | F: nucleotide binding; P: signal transduction; C: intracellular | |
| pco081818 | Clathrin assembly At2g01600 | F: protein binding, lipid binding; P: cellular component organization; C: cytoplasm, membrane | |
| GRMZM2G114918_P01 | Agmatine coumaroyltransferase-1-like | F: transferase activity | |
| cl35669_1 | Pro-MCH partial | C: cytoplasm, membrane | |
| GRMZM2G114992_P01 | Alan shepard (shep)-like |  | |
| GRMZM2G115105_P01 | DETOXIFICATION 12-like | F: transporter activity C: cytoplasm, membrane | |
| TIDP3497 | Cytoplasmic membrane | C: membrane | |
| serk2 | Somatic embryogenesis receptor kinase 1-like | F: nucleotide binding, receptor activity, kinase activity, signal transducer activity; P: cellular protein modification process; C: mitochondria, membrane | |
| GRMZM2G115939_P01 | NEDD8-conjugating enzyme Ubc12-like | F: nucleotide binding, protein binding, transferase activity; P: cellular protein modification process; C: cytoplasm | |
| GRMZM2G115775_P01 | SNARE domain containing | F: protein binding; P: cellular component organization, transport; C: cytoplasm, membrane | |
| AY110296 | Zinc metallo-ase-like |  | |
| umc1316 | 60S ribosomal L22-like | C: mitochondria, membrane, plastid | |
| GRMZM2G116520_P01 | Bowman-Birk type trypsin inhibitor | F: enzyme regulator activity, hydrolase activity; P: protein metabolic process, cellular process; C: extracellular region | |
| GRMZM2G116554_P02 | Amino-acid permease BAT1 homolog | F: transporter activity; C: plasma membrane | |
| GRMZM2G116700_P01 | Autophagy-related 18f | P: response to abiotic stimulus, cell communication, response to extracellular stimulus, response to stress, transport; C: mitochondria | |
| uaz7c01b12 | Ubiquitin conjugating enzyme 2 | F: protein binding, transferase activity; P: catabolic process, cellular protein modification process, response to stress; C: endoplasmic reticulum, membrane | |
| glk6 | TPA: MYB DNA-binding domain superfamily | F: DNA binding; P: biosynthetic process, nucleobase-containing compound metabolic process; C: nucleus | |
| GRMZM2G117410_P01 | Uncharacterized protein LOC100383986 isoform X1 | F: protein binding; P: cellular component organization, transport; C: endosome, membrane, vacuole, mitochondria | |
| GRMZM2G117459_P01 | Uncharacterized protein LOC100191310 | P: biological process | |
| GRMZM2G117507_P01 | Guanosine nucleotide diphosphate dissociation inhibitor 2 | F: enzyme regulator activity; P: signal transduction, metabolic process, transport; C: intracellular | |
| GRMZM2G117544_P01 | Proteasome subunit beta type-3 | F: hydrolase activity; P: protein metabolic process, catabolic process, cellular process; C: nucleus, cytoplasm | |
| Zmrcalm | Calmodulin | F: protein binding, kinase activity; P: response to biotic stimulus, response to abiotic stimulus, response to external stimulus, signal transduction, post-embryonic development, cellular protein modification process; C: cytosol | |
| HIR1 | Hypersensitive-induced response 1 | C: membrane | |
| pco079496 | NADH-ubiquinone oxidoreductase kDa subunit | P: protein metabolic process, catabolic process, cellular component organization, biosynthetic process, response to stress; C: membrane, mitochondria | |
| umc2614 | Signal peptidase complex DTM1 | F: hydrolase; P: protein metabolic process, cellular process; C: membrane, endoplasmic reticulum | |
| GRMZM2G118022_P01 | Uncharacterized protein  LOC100383691 isoform X1 | | |
| gpm191 | Subtilisin-like protease | F: hydrolase activity; P: protein metabolic process; C: cytoplasm | |
| GRMZM2G117823_P01 | SEC14 1 | F: transporter activity; C: intracellular | |
| pco097181 | Uncharacterized protein LOC103632822 | C: cytoplasm, membrane | |
| GRMZM2G118265_P01 | E3 ubiquitin- ligase RGLG1 | F: nucleotide binding, catalytic activity; P: nucleobase-containing compound metabolic process; C: plastid | |
| hsfb | Heat stress transcription factor A-4b-like | F: DNA binding, transcription factor activity, sequence-specific DNA binding; P: biosynthetic process, nucleobase-containing compound metabolic process; C: nucleus, cytoplasm | |
| hsf14 | Heat stress transcription factor A-9 | F: DNA binding, transcription factor activity, sequence-specific DNA binding; P: biosynthetic process, nucleobase-containing compound metabolic process; C: nucleus | |
| pco141010b | Mitochondrial deoxynucleotide carrier | F: structural molecule activity; P: transport, translation; C: mitochondria, membrane, ribosome | |
| cl8986_1 | Actin-related 2 3 complex subunit 4 | F: protein binding; P: cellular component organization; C: cytoskeleton, cytoplasm | |
| GRMZM2G118714_P01 | WEB family At5g55860 | P: cellular component organization; C: cytosol | |
| GRMZM2G118766_P01 | VASP homolog |  | |
| AY110514 | 1-aminocyclopropane-1-carboxylate oxidase homolog 1-like | F: catalytic activity, binding; P: metabolic process | |
| IDP851 | Universal stress A | P: response to stress | |
| GRMZM2G119300_P01 | probable mannose-1-phosphate guanylyltransferase 3 | F: nucleotide binding, transferase activity; P: carbohydrate metabolic process, biosynthetic process, nucleobase-containing compound metabolic process; C: mitochondria | |
| GRMZM2G119370_P01 | S-norcoclaurine synthase-like | F: receptor activity, binding, enzyme regulator activity; P: response to biotic stimulus, signal transduction, response to endogenous stimulus, cellular protein modification process, response to stress; C: nucleus, cytoplasm | |
| GRMZM2G119769_P02 | Snf1-related kinase  Interacting protein SKI1 | | |
| GRMZM2G119802_P01 | Methyl-binding domain-containing 13-like isoform X1 | F: DNA binding; C: nucleus | |
| AY110374 | Glyoxalase II | F: hydrolase activity; P: catabolic process, lipid metabolic process, biosynthetic process, cellular process; C: membrane, endoplasmic reticulum | |
| GRMZM2G120857_P01 | Isocitrate dehydrogenase [NAD] catalytic subunit mitochondrial | F: nucleotide binding, catalytic activity; P: generation of precursor metabolites and energy; C: mitochondria | |
| GRMZM2G121237_P01 | Glycosyltransferase family 64 C5-like | F: transferase activity; P: biosynthetic process, cellular process; C: mitochondria, membrane | |
| cl1285_-2 | Ubiquitin-conjugating enzyme E2 32 | F: protein binding, transferase activity; P: catabolic process, cellular protein modification process; C: endoplasmic reticulum, membrane | |
| GRMZM2G121360_P01 | Heavy metal transport detoxification superfamily | F: binding; P: cellular homeostasis, transport; C: plastid | |
| GRMZM2G121460_P01 | Inosine-5 -monophosphate dehydrogenase-like | F: nucleotide binding, catalytic activity; P: biosynthetic process, nucleobase-containing compound metabolic process; C: membrane, mitochondria | |
| VP15 | Molybdopterin synthase sulfur carrier subunit | F: nucleotide binding, transferase activity; P: signal transduction, response to endogenous stimulus, cellular protein modification process, biosynthetic process; C: cytosol | |
| GRMZM2G121683_P01 | Transmembrane 214-B | C: membrane | |
| GRMZM2G121851_P02 | Calmodulin binding | F: protein binding; P: generation of precursor metabolites and energy, multicellular organism development, cellular component organization, cell differentiation, cellular protein modification process, anatomical structure morphogenesis, photosynthesis, biosynthetic process, nucleobase-containing compound metabolic process, response to stress; C: plastid | |
| GRMZM2G122045_P01 | Transmembrane 184A | F: kinase activity, transporter activity; C: cytoplasm, membrane | |
| GRMZM2G122135_P02 | Serine threonine- phosphatase 2A  65 kDa regulatory subunit A  beta isoform isoform X1 | | |
| GRMZM2G122239_P02 | OTU domain-containing DDB_G0284757 |  | |
| pco087009 | OS-9 homolog | F: carbohydrate binding; P: carbohydrate metabolic process, protein metabolic process, response to abiotic stimulus, catabolic process, biosynthetic process, transport, cellular process, response to stress; C: endoplasmic reticulum | |
| me2 | NADP-dependent malic enzyme | F: nucleotide binding, catalytic activity; P: generation of precursor metabolites and energy; C: plastid | |
| elfg1 | Elongation factor 1-gamma 2 | F: translation factor activity, RNA binding, transferase activity; C: ribosome, nucleus | |
| bzip97 | Basic leucine zipper 43-like | F: DNA binding, transcription factor activity, sequence-specific DNA binding; P: biosynthetic process, nucleobase-containing compound metabolic process; C: intracellular | |
| GRMZM2G122983_P01 | Vacuolar sorting-associated 20 homolog 2-like | P: transport; C: intracellular | |
| GRMZM2G123029_P04 | Aspartic protease | F: hydrolase activity; P: protein metabolic process, catabolic process, lipid metabolic process; C: vacuole | |
| GRMZM2G123309_P01 | Secologanin synthase | F: catalytic activity, binding; P: metabolic process; C: membrane | |
| GRMZM2G123519_P01 | Ubiquitin-conjugating enzyme E2 38 | F: protein binding, transferase activity; P: cellular protein modification process, transport; C: plastid, membrane | |
| nac125 | NAC domain transcription factor superfamily | F: DNA binding, hydrolase activity; P: protein metabolic process, catabolic process, biosynthetic process, nucleobase-containing compound metabolic process; C: nucleus; | |
| GRMZM2G123901_P01 | B-cell receptor-associated 31 | P: transport; C: membrane, endoplasmic reticulum | |
| GRMZM2G123987_P01 | Glycerol-3-phosphate acyltransferase 3 | F: transferase activity; P: metabolic process; C: endoplasmic reticulum, plasma membrane | |
| GRMZM2G124026_P01 | Hypothetical protein | C: cytoplasm, membrane | |
| GRMZM2G124313_P01 | CDP-diacylglycerol--glycerol-3-phosphate 3-phosphatidyltransferase | F: transferase activity; P: lipid metabolic process, biosynthetic process, cellular process; C: plastid, mitochondria, membrane | |
| GRMZM2G124371_P01 | E3 ubiquitin- ligase HERC2 | F: catalytic activity, structural molecule activity, binding; P: transport, translation; C: mitochondria, membrane, ribosome | |
| GRMZM2G124416_P01 | MADS box interactor-like | C: plastid | |
| glk44 | DNA binding | F: DNA binding; P: biosynthetic process, nucleobase-containing compound metabolic process; C: nucleus | |
| GRMZM2G146280_P01 | autophagy-related 18a | F: lipid binding; P: multicellular organism development, response to extracellular stimulus, cellular protein modification process, biosynthetic process, response to stress, response to abiotic stimulus, cell death, cellular component organization; C: cytosol, nucleus, plastid, membrane | |
| GRMZM2G146374_P01 | TPA: ubiquitin-conjugating enzyme family | F: nucleotide binding, protein binding, transferase activity; P: catabolic process, cellular protein modification process; C: cytoplasm | |
| nac30 | NAC domain-containing 92-like isoform X1 | F: DNA binding; P: biosynthetic process, nucleobase-containing compound metabolic process; C: nucleus; | |
| cl31766_1 | Golgin subfamily A member 4 isoform X1 |  | |
| GRMZM2G146553_P01 | CBL-interacting kinase 32 | F: nucleotide binding, kinase activity; P: signal transduction, cellular protein modification process; C: nucleus, mitochondria, membrane | |
| IDP1437 | Ribosomal S19 (mitochondria) | F: RNA binding, structural molecule activity; P: cellular component organization, nucleobase-containing compound metabolic process, translation; C: mitochondria, cytosol, ribosome | |
| GRMZM2G147046_P01 | Transmembrane emp24 domain-containing p24beta3 | P: transport; C: membrane, endoplasmic reticulum, Golgi apparatus, vacuole | |
| cl23538_1a | Fiber Fb34 | C: cytoplasm, membrane | |
| GRMZM2G147446_P01 | Integral membrane like | C: membrane | |
| GRMZM2G147671_P01 | 26S proteasome non-ATPase regulatory subunit 4 homolog | F: protein binding; P: protein metabolic process, catabolic process, cellular component organization; C: cytosol, nucleus | |
| pco065335b | Membrane -like | F: hydrolase activity; P: lipid metabolic process, cellular process; C: cytoplasm, membrane | |
| GRMZM2G147726_P01 | ADP-ribosylation factor 3 | F: nucleotide binding; P: signal transduction; C: cytoplasm, Golgi apparatus, plasma membrane | |
| IDP1628 | OPA3 | P: lipid metabolic process; C: mitochondria | |
| GRMZM2G149031_P01 | Glucosidase 2 subunit beta | C: membrane | |
| pco123975 | UMP CMP kinase 4 | F: nucleotide binding, kinase activity; P: biosynthetic process, nucleobase-containing compound metabolic process; C: nucleus, cytoplasm | |
| pco075430a | Cyclin-dependent kinase family | F: nucleotide binding, kinase activity; P: cellular protein modification process, cell cycle; C: nucleus | |
| GRMZM2G149662_P01 | LIKE COV 2-like | C: membrane | |
| GRMZM2G149717_P01 | Dynamin-related 5A | F: nucleotide binding, hydrolase activity; C: membrane, plastid | |
| GRMZM2G149800_P02 | Uncharacterized protein LOC100277913 isoform X1 | F: catalytic activity, binding; P: metabolic process; C: mitochondria | |
| GRMZM2G150193_P02 | Canopy-1 | P: response to biotic stimulus, response to external stimulus, response to stress, cellular process; C: cytoplasm | |
| TIDP2711 | Galactosylgalactosylxylosyl 3-beta-glucuronosyltransferase 1 | F: transferase activity; P: carbohydrate metabolic process, cellular component organization, biosynthetic process; C: cytoplasm, membrane, Golgi apparatus | |
| GRMZM2G150631_P02 | WD repeat-containing 89 homolog | P: protein metabolic process, nucleobase-containing compound metabolic process | |
| pco061688 | Glucan endo-1,3-beta-glucosidase 4 | F: carbohydrate binding, hydrolase activity; P: carbohydrate metabolic process; C: plasma membrane | |
| GRMZM2G150912_P01 | Diphthine--ammonia ligase | F: nucleotide binding, kinase activity; P: cellular protein modification process | |
| GRMZM2G150941_P02 | RNA-binding 10 isoform X1 | F: nucleic acid binding | |
| PAS2 | Very-long-chain (3R)-3-hydroxyacyl- dehydratase PASTICCINO 2A | F: catalytic activity; P: multicellular organism development, lipid metabolic process, biosynthetic process, cellular process; C: membrane, endoplasmic reticulum | |
| ZmHK1 | Histidine kinase 6 | F: receptor activity, signal transducer activity, hydrolase activity, kinase activity, protein binding; P: embryo development, response to extracellular stimulus, post-embryonic development, cellular protein modification process, anatomical structure morphogenesis, transport, response to stress, response to biotic stimulus, response to abiotic stimulus, response to endogenous stimulus; C: endoplasmic reticulum, plasma membrane | |
| GRMZM2G151406_P01 | Copper-transporting ATPase RAN1 | F: nucleotide binding, hydrolase activity, transporter activity; C: plasma membrane, intracellular | |
| GRMZM2G151580_P01 | AUGMIN subunit 2 | P: cellular component organization, cell cycle | |
| GRMZM2G151582_P01 | Seleno K | C: mitochondria, membrane | |
| wrky95 | WRKY transcription partial | F: DNA binding, transcription factor activity, sequence-specific DNA binding; P: biosynthetic process, nucleobase-containing compound metabolic process; C: intracellular | |
| GRMZM2G148387_P01 | Glutaredoxin subgroup I | F: catalytic activity; P: cellular homeostasis, generation of precursor metabolites and energy; C: cytosol, Golgi apparatus, extracellular region, vacuole, plasma membrane, plastid | |
| GRMZM2G149211_P01 | Peroxisomal adenine nucleotide carrier 1-like | F: structural molecule activity; P: transport, translation; C: membrane, ribosome | |
| GRMZM2G149617_P01 | Sterol 3-beta-glucosyltransferase UGT80B1 | F: transferase activity; P: carbohydrate metabolic process, post-embryonic development, lipid metabolic process, biosynthetic process, reproduction, cellular process; C: membrane, vacuole | |
| GRMZM2G150834_P01 | Germinal-center associated nuclear | C: mitochondria | |
| cl2746_1 | Beta-lactamase 2 isoform X2 | C: membrane, plastid | |
| GRMZM2G147775_P03 | Actin-depolymerizing factor 5 | F: protein binding; P: cellular component organization; C: cytoskeleton | |
| GRMZM2G149406_P01 | Coatomer subunit alpha-3 | F: structural molecule activity; P: transport; C: membrane, mitochondria, Golgi apparatus | |
| GRMZM2G151916_P01 | Shaggy-related kinase eta | F: nucleotide binding, kinase activity; P: cellular protein modification process | |
| GRMZM2G151921_P01 | Inositol 1,4,5-trisphosphate 5-phosphatase | F: hydrolase activity; P: lipid metabolic process, cellular process | |
| GRMZM2G152421_P01 | 26S proteasome non-ATPase regulatory subunit 11 homolog | C: cytosol | |
| pco119600 | Cytochrome b5 domain-containing RLF | F: binding | |
| pco103710 | Purple acid phosphatase 3 | F: hydrolase activity; P: metabolic process, cellular process; C: cytoplasm | |
| GRMZM2G152599_P01 | 30S ribosomal S16 | F: structural molecule activity; P: embryo development, post-embryonic development, cellular component organization, reproduction, translation; C: mitochondria, ribosome, plastid | |
| cl30719_1 | FLX-like 3 |  | |
| pco069906 | Proteasome subunit beta type-6 | F: DNA binding, hydrolase activity; P: protein metabolic process, cell death, catabolic process, biosynthetic process, nucleobase-containing compound metabolic process, response to stress; C: membrane, cytosol, nucleus, vacuole | |
| mkkk63 | ACT-domain containing kinase family | F: nucleotide binding, kinase activity; P: signal transduction, cellular protein modification process; C: cytosol | |
| CaM | Calmodulin-7-like isoform X1 | F: binding; P: response to abiotic stimulus, post-embryonic development | |
| GRMZM2G152929_P03 | ADP-ribosylation factor | F: nucleotide binding; P: signal transduction; C: mitochondria | |
| FBL2 | F-box SKP2A |  | |
| ARF2 | Auxin response factor 22 | F: DNA binding; P: signal transduction, response to endogenous stimulus, biosynthetic process, nucleobase-containing compound metabolic process; C: nucleus | |
| pco093744 | Glycerophosphodiester phosphodiesterase GDPD6 | F: hydrolase activity; P: carbohydrate metabolic process, catabolic process, lipid metabolic process, cellular process; C: cytoplasm | |
| gras69 | Scarecrow 1 | F: DNA binding, transcription factor activity, sequence-specific DNA binding; P: biosynthetic process, nucleobase-containing compound metabolic process; C: nucleus | |
| pmpm2 | Hydrophobic OSR8 | C: cytoplasm, membrane | |
| GRMZM2G153706_P01 | Uncharacterized membrane At4g09580-like | C: membrane, endoplasmic reticulum | |
| umc2338 | Mediator of RNA polymerase II transcription subunit 13 | F: molecular function; P: biosynthetic process, nucleobase-containing compound metabolic process; C: nucleoplasm | |
| GRMZM2G153766_P01 | Mediator of RNA polymerase II transcription subunit 22a | F: molecular function; P: biosynthetic process, nucleobase-containing compound metabolic process; C: nucleoplasm | |
| GRMZM2G153977_P01 | Aspartic protease oryzasin-1 precursor | F: binding, hydrolase activity; P: protein metabolic process, catabolic process, lipid metabolic process, cellular process; C: lysosome | |
| IDP1950 | 14-3-3 GF14-C | F: protein binding; C: nucleus, cytoplasm | |
| pco147721a | Alcohol dehydrogenase-like 2 | F: binding, catalytic activity; P: metabolic process | |
| PT2 | Inorganic phosphate transporter 1-8 | F: transporter activity; C: plasma membrane | |
| GRMZM2G154499_P01 | Iron-sulfur assembly -like mitochondrial | F: binding, structural molecule activity; P: protein metabolic process, cellular component organization, biosynthetic process; C: mitochondria | |
| GRMZM2G154845_P02 | DETOXIFICATION 16-like isoform X1 | F: transporter activity; C: membrane | |
| GRMZM2G155877_P01 | Tetraspanin- partial | C: membrane | |
| GRMZM2G156013_P01 | Serine threonine kinase | F: nucleotide binding, kinase activity; P: cellular protein modification process | |
| GRMZM2G155991_P01 | Phosphatase 2C 44 | F: binding, hydrolase activity; P: cellular protein modification process; C: plastid | |
| ZmRR5 | Orphans transcription partial | F: DNA binding, signal transducer activity; P: response to endogenous stimulus, biosynthetic process, nucleobase-containing compound metabolic process; C: nucleus | |
| cl37643_1 | DEAD-box ATP-dependent RNA helicase 25 | F: nucleotide binding, RNA binding, hydrolase activity; P: protein metabolic process, response to abiotic stimulus, catabolic process, nucleobase-containing compound metabolic process, response to stress; C: mitochondria, membrane, plastid | |
| GRMZM2G157115_P01 | Serine threonine- kinase At5g01020 | F: nucleotide binding, RNA binding, kinase activity; P: cellular protein modification process, nucleobase-containing compound metabolic process | |
| GRMZM2G157316_P01 | Rhomboid 20 | F: hydrolase activity; P: protein metabolic process; C: membrane | |
| TIDP3014 | GTP-binding nuclear Ran-3 | F: nucleotide binding, hydrolase activity; P: signal transduction, transport; C: nucleus, cytoplasm | |
| GRMZM2G158300_P01 | UPF0496 At3g19330-like isoform X1 | F: hydrolase activity; P: protein metabolic process, cellular process, catabolic process; C: extracellular space, membrane, lysosome | |
| pco074498b | Farnesyltransferase subunit beta isoform X1 | F: transferase activity; P: cellular protein modification process; C: mitochondria | |
| pco075569 | Enoyl- hydratase peroxisomal isoform X1 | F: catalytic activity; P: biosynthetic process, cellular process; C: cytoplasm | |
| GRMZM2G158811_P01 | Constitutive expressor of pathogenesis related genes 5 copy 1 | P: response to stress, response to biotic stimulus, response to abiotic stimulus, response to external stimulus, post-embryonic development, reproduction, cellular component organization, cell differentiation, anatomical structure morphogenesis; C: plastid, nucleus, membrane | |
| pco081417 | Transmembrane emp24 domain-containing p24delta9-like | P: transport; C: mitochondria, membrane, endoplasmic reticulum | |
| GRMZM2G158887_P01 | Ras-related Rab7 | F: nucleotide binding; P: signal transduction, transport; C: intracellular, plasma membrane | |
| alf11 | PHD finger | F: DNA binding, protein binding; P: biosynthetic process, nucleobase-containing compound metabolic process; C: nucleus, membrane | |
| GRMZM2G159013_P01 | Ferrochelatase- chloroplastic | F: catalytic activity, P: cellular component organization, catabolic process, cell death, biosynthetic process, transport, response to stress C: mitochondria, membrane, plastid | |
| HDT1 | Histone deacetylase HDT2 | F: hydrolase activity, binding; P: biosynthetic process, nucleobase-containing compound metabolic process; C: nucleolus | |
| GRMZM2G159330_P01 | F-box-like WD repeat-containing TBL1XR1 | F: molecular function; P: response to abiotic stimulus, cellular component organization, cellular protein modification process, biosynthetic process, nucleobase-containing compound metabolic process, response to stress; C: nucleoplasm, cytoplasm | |
| PDIL2-2 | Disulfide isomerase-like 2-2 | F: catalytic activity; P: embryo development, cellular homeostasis, generation of precursor metabolites and energy, post-embryonic development, pollination, response to stress; C: membrane, cell wall, endoplasmic reticulum, extracellular region, vacuole | |
| nac29 | NAC domain-containing 92-like | F: DNA binding; P: biosynthetic process, nucleobase-containing compound metabolic process; C: nucleus | |
| GRMZM2G159660_P01 | Alkaline phosphatase D | C: cytoplasm, membrane | |
| GRMZM2G159756_P01 | Serine threonine- kinase At1g28390 | F: nucleotide binding, kinase activity; P: signal transduction, cellular protein modification process; C: plasma membrane | |
| umc2380 | AT-hook motif nuclear-localized 10 | F: DNA binding | |
| GRMZM2G160273_P05 | Dehydration-induced 19 homolog 4-like | C: mitochondria | |
| GRMZM2G160430_P01 | Solute carrier family facilitated glucose transporter member 8 | F: transporter activity; C: plasma membrane | |
| GRMZM2G160719_P01 | Uncharacterized protein  LOC103636006 | | |
| GRMZM2G160983_P01 | LTV1 homolog isoform X1 | C: mitochondria | |
| mterf21 | Mitochondrial transcription termination factor family | F: DNA binding; P: biosynthetic process, nucleobase-containing compound metabolic process; C: mitochondria | |
| GRMZM2G161299_P01 | Mitochondrial substrate carrier family B | F: structural molecule activity, transporter activity; P: translation; C: mitochondria, membrane, ribosome, plastid | |
| IDP96 | TPA: DUF1664 domain family | C: membrane | |
| GRMZM2G161377_P01 | BPI LBP family At1g04970 | F: lipid binding; C: extracellular space, cytoplasm | |
| GRMZM2G161452_P01 | Serine threonine- kinase isoform X1 | F: nucleotide binding, kinase activity; P: response to biotic stimulus, response to external stimulus, response to endogenous stimulus, cellular protein modification process; C: plasma membrane, plastid | |
| gpm530 | Endo-1,3 1,4-beta-D-glucanase-like | F: hydrolase activity | |
| GRMZM2G161780_P01 | Heptahelical transmembrane 4 | F: transferase activity; P: lipid metabolic process, response to endogenous stimulus, biosynthetic process, cellular process; C: mitochondria, membrane | |
| GRMZM2G162145_P01 | AP-1 complex subunit mu-2 | P: transport; C: cytoplasm, membrane | |
| pco072737a | HMG1 2 |  | |
| csu3 | DeSI At4g17486 |  | |
| GRMZM2G162347_P02 | CTD small phosphatase 2 | F: hydrolase activity; P: cellular protein modification process; C: plastid | |
| pco086069b | Anaphase-promoting complex subunit 11 | F: protein binding, transferase activity; P: catabolic process, cellular protein modification process, cellular component organization, cell cycle; C: nucleus | |
| glk15 | PHR1-LIKE 1-like | F: DNA binding; P: biosynthetic process, nucleobase-containing compound metabolic process; C: nucleus, membrane | |
| GRMZM2G162798_P04 | Hypothetical protein isoform X1 | F: nucleotide binding; P: cellular process; C: membrane | |
| pco125769 | Transmembrane 19-like | C: membrane | |
| GRMZM2G164562_P01 | Chorismate synthase, chloroplastic | F: nucleotide binding, catalytic activity; P: secondary metabolic process, cellular protein modification process, response to stress, biosynthetic process; C: nucleolus, plastid, cytosol | |
| GRMZM2G164821_P01 | Transmembrane 9 superfamily member 3 | C: cytoplasm, membrane | |
| GRMZM2G165060_P01 | Serine threonine- kinase | F: nucleotide binding, kinase activity; P: cellular protein modification process; C: plasma membrane, cytoplasm | |
| GRMZM2G165511_P01 | Ubiquitin-conjugating enzyme E2-17 kDa | F: nucleotide binding, transferase activity; P: catabolic process, cellular protein modification process | |
| GRMZM2G165622_P01 | Cysteine synthase 2 | F: binding; P: biosynthetic process, cellular process; C: cytoplasm, membrane | |
| TIDP3524 | 5-methyl tetrahydro pteroyltriglutamate--homocysteine methyltransferase 1 | F: transferase activity, binding; P: biosynthetic process, cellular process; C: cytosol, extracellular region | |
| GRMZM2G165931_P01 | L-arabinokinase | F: nucleotide binding, kinase activity; C: cytoplasm | |
| GRMZM2G164640_P01 | Wall-associated receptor-like kinase 1 | F: nucleotide binding, kinase activity; P: cellular protein modification process; C: membrane | |
| GRMZM2G166089_P05 | Cullin-1-like | F: protein binding, transferase activity; P: catabolic process, cellular protein modification process; C: intracellular | |
| mir3 | Cysteine protease 1 precursor | F: hydrolase activity; P: protein metabolic process, catabolic process, lipid metabolic process, cellular process; C: lysosome, extracellular space | |
| GRMZM2G166383_P01 | Conserved membrane | C: membrane, plastid | |
| GRMZM2G166459_P01 | Detoxification 16 | F: transporter activity; C: membrane | |
| GRMZM2G166603_P01 | TPA: kinase superfamily | F: nucleotide binding, RNA binding, transferase activity, hydrolase activity; P: protein metabolic process, nucleobase-containing compound metabolic process, transport; C: membrane | |
| GRMZM2G166658_P01 | CBL-interacting kinase 2 | F: nucleotide binding, kinase activity; P: signal transduction, cellular protein modification process; C: membrane | |
| GRMZM2G166694_P04 | Cullin-1-like | F: protein binding, transferase activity; P: catabolic process, cellular protein modification process; C: intracellular | |
| GRMZM2G166767_P01 | Trifunctional UDP-glucose  4,6-dehydratase UDP-4-keto-  6-deoxy-D-glucose 3,5-epimerase  UDP-4-keto-L-rhamnose-reductase  RHM1 | | |
| pco076782 | Inositol phosphorylceramide glucuronosyl transferase 1 | F: transferase activity; P: lipid metabolic process, biosynthetic process, cellular process; C: endosome, cytoplasm, membrane, Golgi apparatus | |
| GRMZM2G167245_P01 | Uncharacterized protein LOC100278420 isoform X1 | C: membrane | |
| GRMZM2G167262_P01 | Signal recognition particle 19 kDa | F: RNA binding; P: cellular component organization, transport C: cytoplasm | |
| GRMZM2G167758_P01 | Nuclear transport factor 2 (NTF2) family | C: plasma membrane | |
| GRMZM2G168393_P01 | Uncharacterized protein LOC100303803 | C: membrane | |
| GRMZM2G168428_P01 | Angio-associated migratory cell | C: cytosol | |
| FPS | Farnesyl pyrophosphate synthase | F: binding, transferase activity; P: lipid metabolic process, biosynthetic process, cellular process; C: cytoplasm | |
| GRMZM2G168744_P01 | Endonuclease 1 | F: nucleic acid binding, nuclease activity; P: catabolic process, DNA metabolic process; C: cytoplasm, membrane | |
| pco088018 | Zinc finger 622 | F: nucleic acid binding; P: biological process; C: cytosol, ribosome | |
| pco096967 | Carbonyl reductase 3 |  | |
| GRMZM2G169020_P01 | Serine threonine- kinase isoform X1 | F: nucleotide binding, kinase activity; P: cellular protein modification process; C: plasma membrane, mitochondria | |
| GRMZM2G169044_P01 | Hypothetical protein ZEAMMB73_137349 | C: plastid | |
| GRMZM2G164665_P01 | Potassium transporter 11 |  | |
| cl11072_-2 | Barley B recombinant | F: transcription factor activity, sequence-specific DNA binding; P: biosynthetic process, nucleobase-containing compound metabolic process; C: nucleus | |
| myb36 | Transcription factor MYB44 | F: DNA binding; C: plastid | |
| IDP634 | Nitrilase cyanide hydratase and apolipo N-acyltransferase family, partial | F: binding, hydrolase activity, transferase activity; P: metabolic process, cellular process; C: cytosol, plastid | |
| GRMZM2G169548_P01 | Acyl-[acyl-carrier- ]-UDP-N-acetylglucosamine O- mitochondrial isoform X1 | F: transferase activity; P: carbohydrate metabolic process; P: lipid metabolic process, biosynthetic process, cellular process; C: mitochondria | |
| GRMZM2G169628_P01 | 7-deoxyloganetin glucosyltransferase-like | F: transferase activity; P: biosynthetic process, cellular process; C: membrane, intracellular | |
| GRMZM2G169694_P01 | Ras-related RABH1b | F: nucleotide binding; P: signal transduction; C: endosome, cytosol, Golgi apparatus, vacuole, plasma membrane | |
| pza02040 | Transmembrane 9 superfamily member 1 | C: cytoplasm, membrane | |
| GRMZM2G165644_P01 | Cysteine-rich receptor kinase 15 | F: nucleotide binding, kinase activity; P: cellular protein modification process, cellular component organization, response to stress, cell growth; C: plasma membrane | |
| GRMZM2G165695_P01 | Hypothetical protein ZEAMMB73_342661 |  | |
| GRMZM2G165939_P01 | Developmentally-regulated G- 2 | F: nucleotide binding | |
| GRMZM2G165998_P01 | 1,2-dihydroxy-3-keto-5-methylthiopentene dioxygenase 2 | F: catalytic activity, binding; P: biosynthetic process, cellular process; C: nucleus, cytoplasm | |
| GRMZM2G169773_P02 | Secretory carrier-associated membrane 2 | P: transport; C: plasma membrane, cytoplasm | |
| gpm41 | Transcription factor LAF1 | F: DNA binding | |
| GRMZM2G170276_P03 | TPA: hypothetical protein  ZEAMMB73_292465 | | |
| GRMZM2G170313_P02 | Prolyl-tRNA synthetase associated domain-containing 1 | F: hydrolase activity; P: translation, nucleobase-containing compound metabolic process; C: membrane, cytosol | |
| gpm915 | RING zinc finger domain superfamily | F: binding, transferase activity; P: catabolic process, cellular protein modification process; C: mitochondria | |
| GRMZM2G170281_P01 | Transmembrane 9 superfamily member 8-like | C: cytoplasm, membrane | |
| GRMZM2G171060_P02 | ADP-ribosylation factor 8A | F: nucleotide binding; P: response to biotic stimulus, response to external stimulus, signal transduction, response to stress; C: intracellular | |
| GRMZM2G171080_P01 | Phosphoinositide phosphatase SAC7-like | F: hydrolase activity; C: membrane | |
| GRMZM2G171118_P01 | TPA: cytochrome P450 superfamily | F: binding, catalytic activity; P: secondary metabolic process, biosynthetic process; C: cytoplasm, membrane | |
| GRMZM2G171139_P01 | Cytochrome P450 94C1 | F: DNA binding, catalytic activity; P: generation of precursor metabolites and energy, biosynthetic process, nucleobase-containing compound metabolic process; C: nucleus, cytoplasm, membrane | |
| GRMZM2G171254_P01 | TPA: hypothetical protein  ZEAMMB73_065337 | | |
| GRMZM2G171430_P01 | Ubiquitin-fold modifier 1 |  | |
| pco148380b | Serine threonine- kinase SAPK8 | F: nucleotide binding, kinase activity; P: response to abiotic stimulus, signal transduction, response to endogenous stimulus, cellular protein modification process, response to stress; C: nucleus, cytoplasm | |
| rpl19 | 50S ribosomal L19, chloroplastic | F: structural molecule activity; P: translation; C: endoplasmic reticulum, ribosome, plastid, mitochondrion, membrane | |
| IDP150 | DNA binding | F: DNA binding; P: biosynthetic process, nucleobase-containing compound metabolic process; C: nucleus | |
| prc4 | 26S protease regulatory subunit, S10B homolog B | F: nucleotide binding, hydrolase activity, protein binding; P: protein metabolic process, catabolic process, cellular component organization, biosynthetic process, nucleobase-containing compound metabolic process, response to stress; C: nucleus, cytosol | |
| GRMZM2G171664_P01 | Cytochrome b-c1 complex subunit mitochondrial | F: catalytic activity, binding, transporter activity; P: generation of precursor metabolites and energy, nucleobase-containing compound metabolic process; C: mitochondria, membrane | |
| GRMZM2G171723_P01 | Uncharacterized protein LOC103633165 | C: plastid | |
| GRMZM2G172210_P01 | BTB POZ and MATH  domain-containing 1-like | | |
| GRMZM2G172230_P01 | Chaperone, chloroplastic | F: nucleotide binding, hydrolase activity; P: protein metabolic process, response to abiotic stimulus, response to stress, cellular process; C: plastid | |
| IDP99 | SPIRAL1-like 1 | C: cytoskeleton, plasma membrane | |
| GRMZM2G172448_P01 | TPA: hypothetical protein  ZEAMMB73_457207, partial | | |
| GRMZM2G172647_P01 | CMP-sialic acid transporter 4 | F: transporter activity; C: cytoplasm, membrane, Golgi apparatus | |
| GRMZM2G172726_P01 | Arabinosyltransferase RRA3-like | F: transferase activity; P: cellular component organization; C: Golgi apparatus, membrane, mitochondria | |
| GRMZM2G172826_P01 | Cytochrome P450 71A1-like | F: catalytic activity, binding; P: secondary metabolic process, biosynthetic process; C: membrane | |
| pco107465a | GDP-mannose transporter GONST3 | C: membrane | |
| GRMZM2G173085_P01 | Uncharacterized protein LOC100384115 | C: membrane | |
| GRMZM2G173119_P01 | AMSH-like ubiquitin thioesterase 3 | C: membrane | |
| pco111077 | ER membrane complex subunit 10 | C: membrane, endoplasmic reticulum, vacuole | |
| Atg4b | Autophagy-related 4b | F: protein binding, hydrolase activity; P: carbohydrate metabolic process, cellular protein modification process, cellular component organization, biosynthetic process, transport, cell cycle; C: nucleus, cytosol, vacuole | |
| umc21 | Tyrosine-phosphatase IBR5 | F: hydrolase activity; P: signal transduction, cellular protein modification process; C: nucleus | |
| GRMZM2G174246_P01 | F-actin-capping subunit alpha | F: protein binding; P: cellular component organization; C: nucleus, cytoskeleton | |
| pco101267 | SelT isoform X2 | C: cytoplasm | |
| RAB2B | Ras-related Rab-2-B | F: nucleotide binding; P: signal transduction, transport; C: cytoplasm, membrane | |
| bzip52 | Transcription factor HBP-1b(c38)-like | F: DNA binding, transcription factor activity, sequence-specific DNA binding; P: biosynthetic process, nucleobase-containing compound metabolic process; C: nucleus | |
| GRMZM2G174286_P01 | Tobamovirus multiplication 3 | C: membrane | |
| GRMZM2G174671_P01 | Arginase mitochondrial | F: binding, hydrolase activity; P: catabolic process, biosynthetic process, cellular process; C: mitochondria, plastid | |
| pco063791 | Unknown |  | |
| GRMZM2G174732_P01 | 4-coumarate- ligase 1 | F: nucleotide binding, catalytic activity; P: secondary metabolic process, biosynthetic process, cellular process; C: membrane | |
| umc1490 | Programmed cell death 5 | F: DNA binding; C: cytosol | |
| GRMZM2G174926_P01 | E3 ubiquitin- ligase HIP1 isoform X1 | F: protein binding, transferase activity; P: catabolic process, cellular protein modification process, growth | |
| GRMZM2G175177_P01 | Replication A 32 kDa subunit B-like | F: DNA binding, protein binding; P: biosynthetic process, DNA metabolic process, response to stress; C: nucleus | |
| GRMZM2G175661_P01 | Zinc finger | F: binding | |
| acp | Acyl carrier 3 | F: binding; P: lipid metabolic process, biosynthetic process, cellular process; C: plastid | |
| GRMZM2G176029_P01 | UDP-galactose UDP-glucose transporter 3-like | F: transporter activity; C: membrane, Golgi apparatus, endoplasmic reticulum | |
| GRMZM2G176355_P01 | Hypothetical protein ZEAMMB73_353506 | C: membrane | |
| GRMZM2G176375_P01 | TPA: bifunctional inhibitor LTP seed storage family | F: lipid binding; P: transport | |
| GRMZM2G176396_P02 | Proline iminopeptidase | F: hydrolase activity; P: protein metabolic process; C: cytosol, plastid | |
| PDIL5-2 | Disulfide isomerase-like 5-2 | F: catalytic activity; P: cellular homeostasis, response to stress; C: membrane, endoplasmic reticulum | |
| GRMZM2G176612_P02 | ADP-ribosylation factor GTPase-activating AGD8 | F: enzyme regulator activity | |
| nac120 | NAC domain-containing 82 | F: DNA binding; P: biosynthetic process, nucleobase-containing compound metabolic process; C: nucleus | |
| GRMZM2G176735_P02 | Vam6 Vps39 | P: cellular component organization, transport; C: intracellular, membrane | |
| GRMZM2G176774_P01 | Beta-1,3-galactosyltransferase 19 | F: transferase activity, carbohydrate binding; P: cellular protein modification process, biosynthetic process; C: Golgi apparatus, membrane, mitochondria | |
| pco140287 | Myosin-binding 1 isoform X1 | C: membrane | |
| GRMZM2G177263_P02 | Transcription initiation factor IIE subunit beta | F: DNA binding, translation factor activity, RNA binding; P: nucleobase-containing compound metabolic process; C: ribosome, nucleoplasm | |
| umc2627 | Dolichyl-diphosphooligosaccharide-- glycosyltransferase subunit 1B | F: transferase activity; P: cellular protein modification process; P: biosynthetic process; C: endoplasmic reticulum, vacuole, plasma membrane, cell wall | |
| uce2 | Ubiquitin conjugating enzyme 2 | F: protein binding, transferase activity; P: catabolic process, cellular protein modification process, response to stress; C: membrane, endoplasmic reticulum | |
| GRMZM2G177324_P01 | Ubiquitin-like-specific protease partial | F: DNA binding, hydrolase activity; P: protein metabolic process; C: membrane, nucleus | |
| cl18707_1 | Beta-D-xylosidase 6 | F: hydrolase activity; P: carbohydrate metabolic process, catabolic process, nucleobase-containing compound metabolic process; C: membrane, cell wall, vacuole | |
| ada2 | Transcriptional adapter ADA2 | F: DNA binding, transcription factor activity, sequence-specific DNA binding, chromatin binding, transferase activity; P: cellular protein modification process, cellular component organization, biosynthetic process, nucleobase-containing compound metabolic process; C: nucleoplasm | |
| GRMZM2G178209_P01 | UDP-glycosyltransferase 73C1-like | F: transferase activity; P: biosynthetic process, cellular process C: intracellular | |
| GRMZM2G178244_P01 | VAMP YKT61 | F: DNA binding, protein binding; P: cellular component organization, transport; C: nucleus, cytoplasm, membrane | |
| GRMZM2G178576_P06 | PRA1 family F3-like | C: membrane | |
| GRMZM2G178618_P01 | Coatomer subunit gamma-2 | F: structural molecule activity; P: transport; C: cytoplasm, membrane, Golgi apparatus | |
| GRMZM2G178289_P03 | Hypothetical protein ZEAMMB73_925141 | C: membrane | |
| TIDP2673 | Aldo-keto reductase family 4 member C9-like | F: nucleic acid binding, catalytic activity; P: metabolic process | |
| GRMZM2G178880_P01 | Mannan synthase 4 | F: transferase activity; P: cellular component organization; C: Golgi apparatus, cytoplasm, membrane | |
| GRMZM2G178906_P02 | OTU domain-containing DDB_G0284757 |  | |
| nac131 | NAC domain-containing 7 | F: DNA binding; P: biosynthetic process, nucleobase-containing compound metabolic process; C: nucleus | |
| pco128668 | Ras-related RABE1e-like | F: nucleotide binding; P: signal transduction, transport; C: plastid | |
| GRMZM2G179301_P02 | Endo-1,3 1,4-beta-D-glucanase-like | F: hydrolase activity | |
| GRMZM2G179155_P01 | Uncharacterized protein LOC100275347 | C: endoplasmic reticulum | |
| GRMZM2G179351_P01 | C2 and GRAM domain-containing At1g03370-like | C: membrane | |
| GRMZM2G179411_P01 | DDB1- and CUL4-associated factor 8 |  | |
| pco074808b | Catalytic hydrolase | F: hydrolase activity; P: metabolic process | |
| GRMZM2G179528_P03 | Serine carboxypeptidase-like 18 | F: transferase activity, hydrolase activity; P: secondary metabolic process, protein metabolic process, catabolic process, cellular process; C: cytoplasm, membrane | |
| GRMZM2G179550_P01 | Hypothetical protein ZEAMMB73_468807 |  | |
| GRMZM2G179662_P01 | Serine threonine- kinase vps15 isoform X1 | F: nucleotide binding, kinase activity; P: multicellular organism development, cellular protein modification process, cellular component organization, transport, pollination; C: nuclear envelope, endosome, membrane, vacuole | |
| GRMZM2G179685_P03 | Cinnamoyl- reductase 1-like | F: catalytic activity; P: lipid metabolic process, biosynthetic process, cellular process | |
| GRMZM2G180211_P01 | Cell division control 50 | P: cellular process; C: membrane, plastid | |
| GRMZM2G180254_P01 | Disease resistance RPP13 3 | F: nucleotide binding | |
| pco139265 | Phosphatase 2C 41 | F: binding, hydrolase activity; P: cellular protein modification process; C: cell | |
| GRMZM2G180558_P01 | TPA: PYM | P: cell cycle; C: cytoplasm, nucleolus, nucleoplasm | |
| GRMZM2G180578_P01 | 26S proteasome non-ATPase regulatory subunit 3 | F: enzyme regulator activity; P: protein metabolic process, catabolic process, cellular process; C: plastid | |
| gpt | Glucose-6-phosphate translocator | F: transporter activity; C: membrane, plastid | |
| GRMZM2G180975_P01 | Splicing arginine serine-rich 19-like | F: nucleotide binding; P: signal transduction; C: plastid | |
| GRMZM2G181018_P01 | UPF0664 stress-induced | C: mitochondria, plasma membrane | |
| GRMZM2G181259_P01 | Beta-glucosidase 3B-like | F: hydrolase activity; P: carbohydrate metabolic process, catabolic process | |
| cl3035_1 | Short-chain dehydrogenase reductase family 42E member 1 | F: catalytic activity; P: lipid metabolic process, biosynthetic process, cellular process; C: membrane, endoplasmic reticulum | |
| GRMZM2G180930_P03 | Ribose-phosphate pyrophosphokinase 4 | F: nucleotide binding, kinase activity; P: biosynthetic process, nucleobase-containing compound metabolic process; C: plasma membrane, cytosol | |
| GRMZM2G180988_P02 | Villin- expressed | F: protein binding; P: cellular component organization | |
| TIDP2951 | HLH DNA-binding domain superfamily | F: protein binding; C: nucleus | |
| GRMZM2G180384_P01 | Proteasome maturation factor UMP1 family | P: cellular component organization; C: nucleus, cytoplasm | |
| umc1450 | Unknown |  | |
| pco128617 | FAM136A | C: membrane | |
| pco080082 | Acyl carrier mitochondrial-like | F: binding; P: lipid metabolic process, biosynthetic process, cellular process; C: mitochondria | |
| GRMZM2G181566_P01 | UPF0496 1 | C: membrane, plastid | |
| nac2 | NAC domain-containing 92-like | F: DNA binding; P: biosynthetic process, nucleobase-containing compound metabolic process; C: nucleus | |
| GRMZM2G300125_P01 | Phosphatase 2C family | F: binding, hydrolase activity; P: cellular protein modification process; C: cell | |
| GRMZM2G301122_P01 | Trihelix transcription factor |  | |
| GRMZM2G309933_P02 | Hypothetical protein isoform X1 | | |
| GRMZM2G312877_P01 | Lactoylglutathione lyase | F: catalytic activity | |
| GRMZM2G318180_P01 | Grx_I1-glutaredoxin  subgroup III | F: catalytic activity, binding; P: cellular homeostasis, metabolic process; C: cell | |
| GRMZM2G302279_P01 | Disease resistance RGA2 | F: ADP binding | |
| GRMZM2G313184_P01 | Peroxidase 47 | F: binding, catalytic activity; P: catabolic process, cellular component organization, response to stress; C: extracellular region, cytoplasm, cell wall | |
| GRMZM2G313320_P01 | Uncharacterized protein  LOC103651693 | | |
| GRMZM2G314233_P01 | 26S proteasome non-ATPase regulatory subunit 2 homolog A-like | F: enzyme regulator activity, hydrolase activity; P: protein metabolic process, catabolic process, cellular process; C: nucleus, membrane, cytosol | |
| GRMZM2G314386_P01 | L-type lectin-domain containing receptor kinase | F: nucleotide binding; P: signal transduction; C: intracellular | |
| GRMZM2G314955_P01 | GEM 1 | F: peptidyl-prolyl cis-trans isomerase activity, isomerase activity; P: protein peptidyl-prolyl isomerization, protein folding | |
| GRMZM2G315125_P01 | ABC1-like partial | F: nucleotide binding, kinase activity; P: cellular protein modification process; C: plastid | |
| GRMZM2G315769_P01 | TPA: CBL-interacting serine threonine- kinase 15 | F: nucleotide binding, kinase activity; P: signal transduction, cellular protein modification process; C: membrane | |
| cl12749_1 | Auxin-responsive SAUR36-like | F: protein binding; P: signal transduction, response to endogenous stimulus, transport, growth; C: mitochondria | |
| GRMZM2G319465_P03 | 5-methylthioadenosine S-adenosylhomocysteine deaminase-like | F: hydrolase activity; P: catabolic process, nucleobase-containing compound metabolic process | |
| GRMZM2G316967_P01 | Topless-related 2 isoform X1 | P: biosynthetic process, nucleobase-containing compound metabolic process | |
| AY104566 | Proteasome maturation factor UMP1 family | P: cellular component organization; C: nucleus, cytoplasm | |
| GRMZM2G319781_P01 | Phosphatidylinositol transfer 2-like | P: transport; C: intracellular | |
| GRMZM2G320949_P01 | ATG8-interacting 1 | C: membrane, integral component of membrane | |
| pco096212 | E3 ubiquitin- ligase LIN-1 | C: plastid | |
| GRMZM2G322950_P01 | VQ motif family |  | |
| GRMZM2G323672_P02 | Phox Bem1p | F: kinase activity | |
| TIDP3299 | UDP-glucose 6-dehydrogenase 5 | F: nucleotide binding, catalytic activity; P: carbohydrate metabolic process, biosynthetic process, nucleobase-containing compound metabolic process; C: nucleus, cytosol | |
| RAB2A | Ras-related Rab-2-B | F: nucleotide binding; P: signal transduction transport; C: membrane, cytoplasm | |
| pco110570 | AP-2 complex subunit mu | P: transport; C: cytoplasm, membrane | |
| pco131734 | Caffeoyl- O-methyltransferase 1 | F: transferase activity, binding; P: metabolic process; C: nucleus | |
| GRMZM2G333875_P01 | Cysteine-rich receptor kinase 23 isoform X4 | F: nucleotide binding, kinase activity; P: cellular protein modification process | |
| GRMZM2G335521_P01 | Polyadenylate-binding 2 | F: nucleotide binding, RNA binding; C: cytoplasm | |
| gst19 | Glutathione transferase | F: transferase activity; P: secondary metabolic process, catabolic process, cellular process; C: cytoplasm | |
| GRMZM2G338691_P01 | L-type lectin-domain containing receptor kinase | F: nucleotide binding, carbohydrate binding, kinase activity; P: cellular protein modification process; C: cytoplasm, membrane | |
| GRMZM2G345081_P01 | Little Zipper 1-like |  | |
| GRMZM2G346639_P01 | Non-canonical poly(A) RNA polymerase PAPD5 | F: transferase activity | |
| P4H3 | Prolyl 4-hydroxylase 9 | F: catalytic activity, binding; P: metabolic process, response to stress; C: cytoplasm, membrane, Golgi apparatus | |
| GRMZM2G348866_P02 | mRNA-decapping enzyme | F: RNA binding, enzyme regulator activity; P: catabolic process, nucleobase-containing compound metabolic process; C: cytoplasm | |
| GRMZM2G351074_P01 | Serine threonine- kinase BLUS1 isoform X2 | F: nucleotide binding, signal transducer activity, kinase activity; P: cellular protein modification process; C: plasma membrane, cytoplasm | |
| GRMZM2G354579_P02 | Uncharacterized protein LOC103651651 isoform X1 | C: plastid | |
| GRMZM2G359397_P01 | TPA: VTC2 | F: transferase activity; P: carbohydrate metabolic process; C: cytoplasm | |
| GRMZM2G360455_P01 | Phosphatase 2C 11 | F: binding, hydrolase activity; P: cellular protein modification process; C: cell | |
| gpm517 | Transcription factor HBP-1b(c38)-like | F: DNA binding, transcription factor activity, sequence-specific DNA binding; P: biosynthetic process, nucleobase-containing compound metabolic process; C: nucleus | |
| GRMZM2G362883_P02 | E3 ubiquitin- ligase PRT1 | F: binding, transferase activity; P: catabolic process, cellular protein modification process | |
| GRMZM2G363908_P01 | Uncharacterized protein LOC100274728 | C: mitochondria | |
| mdJ1 | Dna J homolog | F: nucleotide binding, protein binding; P: response to abiotic stimulus, cellular process, response to stress | |
| GRMZM2G364208_P01 | Uncharacterized protein  LOC103630767 | | |
| GRMZM2G366392_P01 | S-adenosylmethionine decarboxylase | F: catalytic activity; P: biosynthetic process, nucleobase-containing compound metabolic process; C: cytosol | |
| GRMZM2G366910_P01 | Hypothetical protein  ZEAMMB73_910861, partial | | |
| GRMZM2G367001_P02 | 26S proteasome non-ATPase regulatory subunit 12 homolog A-like | P: protein metabolic process, catabolic process, cellular process; C: nucleus, cytoplasm, membrane | |
| pco145619 | BSD domain containing |  | |
| GRMZM2G368908_P01 | 26S proteasome non-ATPase regulatory subunit 7 homolog A | P: protein metabolic process, catabolic process, cellular process; C: intracellular | |
| GRMZM2G369340_P04 | Uncharacterized Rho GTPase-activating At5g61530 | P: signal transduction; C: cytoplasm | |
| GRMZM2G372077_P01 | Hypothetical protein | F: catalytic activity; P: cellular protein modification process; C: membrane | |
| GRMZM2G372475_P02 | Enhanced Disease Resistance 2-like |  | |
| CYP | Peptidyl-prolyl cis-trans isomerase | F: binding, catalytic activity; P: cellular protein modification process; C: cytoplasm | |
| pco139456 | Mediator of RNA polymerase II transcription subunit 19a-like isoform X1 | C: membrane | |
| GRMZM2G330453_P01 | PNS1 isoform X2 | C: cytoplasm, membrane | |
| GRMZM2G331283_P02 | Amino acid permease 1 | F: transporter activity; C: plasma membrane | |
| GRMZM2G335978_P01 | Aspartic protease 1 | F: hydrolase activity; P: protein metabolic process, catabolic process; C: plasma membrane, cytoplasm | |
| GRMZM2G341404_P01 | Dna J P58IPK homolog | P: cellular process; C: endoplasmic reticulum, plasma membrane | |
| GRMZM2G341918_P02 | Callose synthase 3-like isoform X1 | F: transferase activity; P: carbohydrate metabolic process, biosynthetic process, cellular process; C: plasma membrane | |
| GRMZM2G354604_P01 | GTP-binding nuclear Ran-2 | F: nucleotide binding, hydrolase activity; P: signal transduction, transport; C: nucleus, cytoplasm | |
| GRMZM2G354610_P03 | HGA4 isoform X1 | F: transferase activity; C: membrane | |
| GRMZM2G357112_P01 | Protein YLS9-like | F: signal transducer activity; P: response to stress; C: plasma membrane | |
| GRMZM2G358311_P01 | Transmembrane 131 homolog | P: metabolic process, cellular process; C: membrane, plastid | |
| GRMZM2G327234_P01 | Potassium transporter 11 | F: transporter activity; P: cellular process; C: membrane | |
| pco133868b | Exocyst complex component SEC8 | F: lipid binding; P: post-embryonic development, biosynthetic process, transport, cellular process, reproduction; C: cytosol, plasma membrane | |
| bzip50 | Ocs element-binding factor 1 | F: DNA binding, transcription factor activity, sequence-specific DNA binding; P: biosynthetic process, nucleobase-containing compound metabolic process; C: intracellular | |
| GRMZM2G339820_P01 | VIN3 1 |  | |
| GRMZM2G372870_P01 | Dna J homolog subfamily B member 1 | F: protein binding; P: cellular process | |
| GRMZM2G372930_P01 | Kelch domain-containing 4 |  | |
| GRMZM2G374881_P02 | Proteasome subunit beta type-6 | F: hydrolase activity, DNA binding; P: protein metabolic process, cell death, catabolic process, biosynthetic process, nucleobase-containing compound metabolic process, response to stress; C: nucleus, membrane, cytosol, vacuole | |
| GRMZM2G373329_P01 | U-box domain-containing 33-like isoform X1 | F: transferase activity; P: cellular protein modification process | |
| umc1549a | Calcitonin peptide-receptor component | F: nucleotide binding, transferase activity; P: biosynthetic process, nucleobase-containing compound metabolic process; C: nucleoplasm | |
| GRMZM2G375807_P01 | ABC transporter D family member 1 | F: nucleotide binding, transporter activity, hydrolase activity; C: mitochondria, membrane | |
| GRMZM2G376918_P03 | Pentatricopeptide repeat-containing mitochondrial | F: catalytic activity; P: cellular protein modification process; C: mitochondria | |
| GRMZM2G380184_P01 | Cullin-3A-like isoform X1 | F: protein binding, transferase activity; P: catabolic process, cellular protein modification process; C: intracellular | |
| GRMZM2G381025_P01 | Anthocyanidin 5,3-O-glucosyltransferase | F: transferase activity; P: biosynthetic process, cellular process; C: intracellular | |
| GRMZM2G382581_P01 | NAC domain transcription factor superfamily isoform 1 | F: DNA binding; P: transcription, DNA-templated, regulation of transcription; C: nucleus | |
| GRMZM2G382774_P01 | EARLY flowering 4 isoform X1 |  | |
| GRMZM2G383240_P05 | CBL-interacting kinase 8 | F: nucleotide binding, kinase activity; P: signal transduction, multicellular organism development, cellular protein modification process; C: plasma membrane, nucleus, cytoplasm | |
| GRMZM2G383338_P01 | TPA: hypothetical protein ZEAMMB73_240948 | C: membrane | |
| GRMZM2G384972_P01 | Peroxisomal membrane 11-3 | P: cellular component organization; C: peroxisome, membrane | |
| GRMZM2G385989_P01 | Vesicle-associated 1-3 | C: membrane, endoplasmic reticulum | |
| IDP3821 | Erwinia-induced 2 | P: transport; C: membrane, endoplasmic reticulum | |
| GRMZM2G386590_P01 | 14-3-3 GF14 partial | F: protein binding; C: membrane | |
| GRMZM2G386998_P01 | Inactive purple acid phosphatase 1 | F: binding, hydrolase activity; P: metabolic process, cellular process; C: cytoplasm, membrane | |
| GRMZM2G387076_P02 | VAMP YKT61 | F: DNA binding, protein binding; P: cellular component organization, transport; C: nucleus, cytoplasm, membrane | |
| GRMZM2G387360_P01 | Nonspecific lipid-transfer precursor | F: lipid binding, hydrolase activity; P: protein metabolic process, transport; C: cytoplasm | |
| PDIL2-3 | Disulfide isomerase-like 2-3 | F: catalytic activity; P: cellular homeostasis, response to stress C: extracellular region, membrane, endoplasmic reticulum | |
| GRMZM2G390374_P01 | ROOT HAIR DEFECTIVE 3 homolog 1 | F: nucleotide binding, hydrolase activity; C: membrane, endoplasmic reticulum | |
| GRMZM2G388576_P01 | 26S proteasome non-ATPase regulatory subunit 14 homolog | P: generation of precursor metabolites and energy, catabolic process, post-embryonic development, cellular protein modification process, biosynthetic process, nucleobase-containing compound metabolic process, transport, response to stress, carbohydrate metabolic process, response to abiotic stimulus, lipid metabolic process, cellular component organization; C: cytosol, nucleus | |
| GRMZM2G387381_P01 | Cysteine-rich receptor kinase 19 | F: nucleotide binding, kinase activity; P: cellular protein modification process, response to stress; C: cytoplasm, plasma membrane | |
| GRMZM2G391000_P01 | Galacturonosyl  transferase 4 | F: transferase activity; P: carbohydrate metabolic process, cellular component organization, biosynthetic process, nucleobase-containing compound metabolic process; C: cytoplasm, membrane, Golgi apparatus | |
| GRMZM2G392320_P01 | RING zinc finger domain superfamily | F: binding | |
| GRMZM2G395061_P01 | Trichome birefringence-like 13 | F: transferase activity; P: biosynthetic process, cellular process; C: mitochondria, membrane, Golgi apparatus | |
| ARF1 | ADP-ribosylation factor 1 | F: nucleotide binding, hydrolase activity, transporter activity; P: signal transduction; C: Golgi apparatus | |
| pco104752 | NADH dehydrogenase [ubiquinone] iron-sulfur, mitochondrial | F: binding, catalytic activity; P: response to abiotic stimulus, generation of precursor metabolites and energy, response to stress; C: membrane, C: mitochondria | |
| GRMZM2G396856_P01 | Polyamine oxidase 2 | F: transferase activity; P: metabolic process; | |
| GRMZM2G397055_P01 | Alcohol dehydrogenase-like 7 | F: binding, catalytic activity; P: metabolic process | |
| GRMZM2G397557_P01 | Disease resistance RGA3 | F: nucleotide binding; C: membrane | |
| IDP290 | Methylglutaconyl- hydratase | F: catalytic activity; P: metabolic process; C: mitochondria | |
| pco084191 | TPA: WRKY DNA-binding domain superfamily | F: DNA binding, transcription factor activity, sequence-specific DNA binding; P: biosynthetic process, nucleobase-containing compound metabolic process; C: nucleus | |
| GRMZM2G399067_P01 | SNF1-type serine-threonine kinase | F: nucleotide binding, kinase activity; P: signal transduction, response to endogenous stimulus, cellular protein modification process; C: plasma membrane, nucleus, cytoplasm | |
| GRMZM2G400169_P01 | Cytochrome C oxidase biogenesis Cmc1 | F: transferase activity; P: catabolic process, cellular protein modification process | |
| GRMZM2G406196_P01 | UPF0496 2 | C: membrane | |
| TIDP3103 | DNA-directed RNA polymerase III subunit RPC6 | F: transferase activity; P: biosynthetic process, nucleobase-containing compound metabolic process; C: plastid, nucleoplasm | |
| GRMZM2G406603_P01 | Palmitoyl-acyl carrier chloroplastic | F: hydrolase activity, binding, transferase activity; P: lipid metabolic process, biosynthetic process, transport, cellular process; C: plastid, cytosol | |
| GRMZM2G408537_P01 | Uncharacterized protein LOC103636108 | C: membrane | |
| GRMZM2G411653_P01 | Leucine Rich Repeat family expressed |  | |
| GRMZM2G408706_P03 | Violaxanthin de-chloroplastic | F: transferase activity; P: lipid metabolic process, biosynthetic process; C: plastid | |
| GRMZM2G412888_P01 | Bem46-like isoform X1 | F: hydrolase activity; P: protein metabolic process; C: cytoplasm, membrane | |
| pco130711 | Urease accessory G | F: nucleotide binding, hydrolase activity; P: metabolic process; C: membrane | |
| GRMZM2G414955_P01 | Hypothetical protein ZEAMMB73_432538 | F: nucleotide binding, nucleic acid binding, ATP binding, ATP-dependent helicase activity, helicase activity, RNA binding, ATP-dependent RNA helicase activity, hydrolase activity; P: mRNA splicing, via spliceosome; C: cytoplasm, spliceosomal complex | |
| GRMZM2G415327_P03 | Rac-like GTP-binding 3 | F: nucleotide binding, protein binding; P: signal transduction; C: membrane, cytoplasm | |
| gst23 | Glutathione transferase GST 23-like | F: transferase activity; P: secondary metabolic process, catabolic process, cellular process, response to stress; C: cytoplasm | |
| GRMZM2G416817_P02 | Triacylglycerol lipase | F: hydrolase activity; P: lipid metabolic process; C: membrane | |
| GRMZM2G416965_P01 | Bidirectional sugar transporter SWEET6b | F: transporter activity; C: plasma membrane | |
| GRMZM2G418160_P01 | ROOT HAIR DEFECTIVE 3 homolog 2-like isoform X2 | F: nucleotide binding, hydrolase activity; C: membrane, endoplasmic reticulum | |
| Atg8b | Autophagy-related 8c | P: cell communication, response to extracellular stimulus, transport, response to stress; C: cytoskeleton, membrane, vacuole | |
| ubi2 | Polyubiquitin | C: cytoplasm | |
| GRMZM2G420713_P01 | Nucleic acid binding | F: nucleotide binding, nucleic acid binding | |
| GRMZM2G420883_P01 | Unknown |  | |
| GRMZM2G421604_P01 | Folate-biopterin transporter 4 isoform X1 | P: transport; C: membrane, plastid | |
| c3h22 | Zinc finger CCCH domain-containing 8-like | F: binding | |
| myb115 | MYB DNA-binding domain superfamily | F: DNA binding, kinase activity; P: biosynthetic process | |
| GRMZM2G424783_P02 | Hypothetical protein ZEAMMB73_436424 | P: transport; C: membrane | |
| GRMZM2G425719_P03 | Flavin-containing monooxygenase FMO GS-OX-like 2 | F: nucleotide binding, catalytic activity; P: metabolic process | |
| GRMZM2G432083_P01 | Cation-chloride cotransporter 1 isoform X1 | F: transporter activity; P: cellular process; C: membrane | |
| GRMZM2G432390_P01 | ABC transporter B family member, chloroplastic | F: nucleotide binding, transporter activity, hydrolase activity; C: membrane, plastid | |
| AY107726 | Amino acid permease 3-like | F: transporter activity; C: plasma membrane | |
| GRMZM2G436583_P01 | Cyclic nucleotide-gated ion channel 17 | F: transporter activity; P: cellular process; C: plasma membrane | |
| GRMZM2G436835_P01 | AP-1 complex subunit mu-2 | P: transport; C: cytoplasm, membrane | |
| GRMZM2G437490_P01 | Leucine zipper -like | P: transport, cellular process; C: plastid | |
| GRMZM2G439201_P02 | Elongation factor 1-beta | F: translation factor activity, RNA binding; C: ribosome | |
| GRMZM2G439950_P01 | Envelope glycoprotein | C: cellular component | |
| pco085840 | Sugar phosphate/phosphate translocator At3g11320 | C: membrane | |
| GRMZM2G443655_P01 | TPA: hypothetical protein  ZEAMMB73_728277 | | |
| mads52 | 26S proteasome non-ATPase regulatory subunit 14 homolog | F: DNA binding, protein binding; P: generation of precursor metabolites and energy, catabolic process, post-embryonic development, cellular protein modification process, biosynthetic process, nucleobase-containing compound metabolic process, transport, response to stress, carbohydrate metabolic process, response to abiotic stimulus, lipid metabolic process, cellular component organization; C: cytosol, nucleus | |
| GRMZM2G447791_P01 | Hypothetical protein ZEAMMB73_211749 |  | |
| pr5 | Thaumatin, partial |  | |
| GRMZM2G403076_P01 | Inactive tetrahydrocannabinolic acid synthase-like | F: nucleotide binding, DNA binding, lipid binding, catalytic activity, receptor activity, signal transducer activity; P: response to endogenous stimulus, biosynthetic process, nucleobase-containing compound metabolic process; C: nucleus, cytoplasm | |
| GRMZM2G406119_P01 | VAMP SEC22 | F: signal transducer activity; P: response to stress; C: plasma membrane | |
| GRMZM2G426553_P01 | Pentatricopeptide repeat-containing At5g52630 | F: zinc ion binding, microtubule-severing ATPase activity, hydrolase activity | |
| GRMZM2G428168_P01 | Glutathione S-transferase | F: transferase activity; P: secondary metabolic process, catabolic process, cellular process; C: cytoplasm | |
| GRMZM2G435373_P01 | Guanosine nucleotide diphosphate dissociation inhibitor 2 | F: enzyme regulator activity; P: signal transduction, metabolic process, transport; C: intracellular | |
| GRMZM2G440003_P01 | Salicylic acid-binding 2-like | F: hydrolase activity, P: lipid metabolic process | |
| GRMZM2G441798_P01 | Sec1 family domain-containing MIP3 | P: transport, cellular process; C: membrane | |
| tac901.2 | Sulfate transporter | F: transporter activity; C: plasma membrane | |
| IDP149 | Proteasome subunit alpha type 3 | F: hydrolase activity; P: protein metabolic process, catabolic process, cellular process; C: nucleus, membrane, cytosol, extracellular region, vacuole | |
| myb163 | Myb-related 308 | F: DNA binding | |
| GRMZM2G441937_P01 | TPA: hypothetical protein  ZEAMMB73_483327 | | |
| bzip113 | TPA: bZIP transcription  factor superfamily | F: DNA binding, transcription factor activity, sequence-specific DNA binding; P: biosynthetic process, nucleobase-containing compound metabolic process; C: intracellular | |
| shpl1 | Endoplasmin homolog | F: nucleotide binding, protein binding; P: anatomical structure morphogenesis, transport, cellular process, response to stress, growth, response to abiotic stimulus; C: mitochondria, endoplasmic reticulum, extracellular region, vacuole, plasma membrane, plastid, nucleus | |
| GRMZM2G404702_P01 | Copper-transporting ATPase RAN1 | F: nucleotide binding, transporter activity, hydrolase activity; C: plasma membrane, intracellular | |
| GRMZM2G434277_P01 | STRUBBELIG-RECEPTOR FAMILY 3 isoform X1 | F: nucleotide binding, kinase activity; P: cellular protein modification process; C: cytoplasm, membrane | |
| GRMZM2G448258_P02 | Cyclin-dependent kinase C-3 | F: nucleotide binding, kinase activity; P: cellular protein modification process, cell cycle | |
| TIDP3450 | Acylpyruvase mitochondrial | F: hydrolase activity; P: metabolic process; C: mitochondria | |
| GRMZM2G448456_P01 | Thioredoxin domain-containing 9 homolog | F: transferase activity; P: cellular homeostasis, biosynthetic process, nucleobase-containing compound metabolic process; C: cytoplasm | |
| mlip15 | bZIP transcription factor 53-like | F: DNA binding, transcription factor activity, sequence-specific DNA binding; P: biosynthetic process, nucleobase-containing compound metabolic process; C: plastid, nucleus | |
| GRMZM2G449163_P01 | Phosphatidylinositol-3-phosphatase myotubularin-1-like | F: hydrolase activity; P: cellular protein modification process; C: plastid | |
| GRMZM2G449177_P01 | Phosphatidylinositol-3-phosphatase myotubularin-1 | F: hydrolase activity; P: cellular protein modification process | |
| GRMZM2G451281_P02 | Cytochrome b-c1 complex subunit 6 | F: transporter activity, catalytic activity; P: generation of precursor metabolites and energy, nucleobase-containing compound metabolic process; C: mitochondria, membrane | |
| GRMZM2G451327_P01 | Geranylgeranyl transferase type-1 subunit beta | F: transferase activity; P: response to abiotic stimulus, response to endogenous stimulus, cellular protein modification process, response to stress; C: intracellular | |
| GRMZM2G452633_P02 | WVD2-like 1 | C: plastid | |
| pco120949 | Calcium permeable stress-gated cation channel 1 | C: cytoplasm, membrane | |
| GRMZM2G457267_P01 | MPPN domain containing | F: nucleotide binding, hydrolase activity, DNA binding, lipid binding, transporter activity; P: cellular component organization, biosynthetic process, nucleobase-containing compound metabolic process; C: nuclear envelope, membrane | |
| TIDP3101 | DNA-directed RNA polymerases IV and V subunit 4 | F: nucleotide binding, DNA binding, RNA binding, protein binding, transferase activity; P: catabolic process, nucleobase-containing compound metabolic process, transport, response to stress, translation, response to abiotic stimulus, cellular component organization; C: cytoplasm, nucleolus, nucleoplasm | |
| GRMZM2G454556_P01 | Defence-related precursor | P: response to biotic stimulus, response to external stimulus, response to stress; C: cytoplasm, membrane | |
| GRMZM2G457346_P01 | Seed maturation PM41 |  | |
| GRMZM2G457544_P01 | E3 ubiquitin ligase DRIP2 | F: binding | |
| GRMZM2G459291_P02 | Metal-dependent HD subdomain | F: hydrolase activity | |
| GRMZM2G460866_P01 | 14 kDa zinc-binding | F: nucleotide binding, protein binding, hydrolase activity; P: nucleobase-containing compound metabolic process; C: plasma membrane, peroxisome, cytosol | |
| GRMZM2G461557_P05 | Plant UBX domain-containing 1 | F: protein binding; P: cellular component organization, growth; C: membrane | |
| IDP284 | ATP-dependent zinc metalloprotease FTSH mitochondrial | F: nucleotide binding, hydrolase activity; P: protein metabolic process, cellular process; C: mitochondria, membrane | |
| GRMZM2G463267_P01 | 26S proteasome non-ATPase regulatory subunit 13 | F: structural molecule activity; P: protein metabolic process, catabolic process, cellular component organization; C: cytosol, nucleus, membrane | |
| GRMZM2G467059_P03 | 26S proteasome non-ATPase regulatory subunit 14 homolog | P: generation of precursor metabolites and energy, catabolic process, post-embryonic development, cellular protein modification process, biosynthetic process, nucleobase-containing compound metabolic process, transport, response to stress, carbohydrate metabolic process, response to abiotic stimulus, lipid metabolic process, cellular component organization; C: cytosol, nucleus | |
| GRMZM2G467370_P01 | Uncharacterized protein LOC100277379 | C: membrane | |
| GRMZM2G467520_P01 | Hypothetical protein ZEAMMB73_412982 | P: transport; C: cell | |
| GRMZM2G467682_P01 | Uncharacterized protein LOC100275497 | F: metal ion binding | |
| GRMZM2G467893_P01 | Nuclear Fusion Defective  4-like | C: membrane | |
| GRMZM2G467992_P01 | V-type proton ATPase subunit e1 | F: transporter activity, hydrolase activity; P: generation of precursor metabolites and energy, nucleobase-containing compound metabolic process; C: cytoplasm, membrane | |
| pco063192 | Choline ethanolamine kinase | F: kinase activity | |
| GRMZM2G470524_P01 | 7-deoxyloganetin glucosyltransferase-like | F: transferase activity; P: biosynthetic process, cellular process; C: membrane, intracellular | |
| GRMZM2G471065_P02 | Mannan endo-1,4-beta-mannosidase 5 | F: hydrolase activity; P: carbohydrate metabolic process, catabolic process, cellular process; C: membrane | |
| GRMZM2G472226_P01 | Uncharacterized protein  LOC103643999 | | |
| pco083050 | Dynein light chain,  cytoplasmic | P: cellular process; C: cytoskeleton | |
| phd38 | PHD transcription, partial | F: binding, transferase activity; C: membrane | |
| GRMZM2G473976_P01 | Isoprenylcysteine alpha-carbonyl methylesterase ICME isoform X1 | F: DNA binding, hydrolase activity, protein binding; P: biosynthetic process, nucleobase-containing compound metabolic process; C: plastid, nucleus, membrane, Golgi apparatus, endoplasmic reticulum | |
| GRMZM2G474755_P01 | Polcalcin Jun o 2 | F: binding; C: mitochondria | |
| GRMZM2G476040_P01 | Uncharacterized protein LOC103628972 | C: membrane | |
| GRMZM2G477146_P01 | Magnesium transporter NIPA8 | F: transporter activity; C: membrane | |
| GRMZM2G477205_P01 | E3 ubiquitin ligase BIG BROTHER-related-like | F: binding, transferase activity; P: catabolic process, cellular protein modification process | |
| GRMZM2G477314_P01 | PHLOEM PROTEIN 2-LIKE A10 | C: mitochondria, membrane | |
| GRMZM2G478370_P01 | Methyl- -binding domain-containing 13-like | F: DNA binding; C: nucleus | |
| ZmNAS3 | Nicotianamine synthase 3 | F: transferase activity; P: biosynthetic process, transport, cellular process | |
| cl3986_1 | Two-component response regulator ORR23 | F: DNA binding, transcription factor activity, sequence-specific DNA binding; P: signal transduction, response to endogenous stimulus, biosynthetic process, nucleobase-containing compound metabolic process; C: nucleus | |
| OBF1 | Ocs element-binding factor 1 | F: DNA binding, transcription factor activity, sequence-specific DNA binding; P: biosynthetic process, nucleobase-containing compound metabolic process, transport; C: plastid, nucleus | |
| GRMZM2G480364_P01 | RNA-directed DNA methylation 3-like |  | |
| GRMZM2G480516_P01 | Fatty acyl coA reductase | F: catalytic activity; P: secondary metabolic process, cellular process, biosynthetic process; C: membrane, intracellular | |
| GRMZM2G481531_P01 | G-type lectin S-receptor-like serine threonine- kinase At1g34300 | F: nucleotide binding, kinase activity; P: cellular protein modification process; C: mitochondria, membrane | |
| GRMZM2G481755_P01 | Lysophospholipid acyltransferase 1 | F: transferase activity; C: membrane | |
| GRMZM2G528010_P02 | Hypothetical protein ZEAMMB73_273912 | F: structural molecule activity; P: cellular component organization, cell cycle; C: nuclear envelope, cytoskeleton, membrane | |
| GRMZM2G545326_P01 | Ubiquitin-like-specific protease 2B | F: hydrolase activity; P: cellular protein modification process; C: nucleus, membrane | |
| GRMZM2G700148_P01 | AP-2 complex subunit alpha-1-like | F: transporter activity; C: cytoplasm, membrane | |
| GRMZM2G700503_P01 | Hypothetical protein ZEAMMB73_981162 |  | |
| GRMZM2G701144_P02 | Hypothetical protein ZEAMMB73_494173 | P: cellular process; C: cytoplasm, membrane | |
| pco085431 | NADPH:quinone oxidoreductase 1 | F: catalytic activity; P: lipid metabolic process, biosynthetic process | |
| GRMZM2G703104_P01 | Formin 5 |  | |
| GRMZM2G703245_P01 | TPA: hypothetical protein  ZEAMMB73_640009 | | |
| GRMZM2G704222_P01 | Hypothetical protein ZEAMMB73_601722 |  | |
| GRMZM5G800558_P01 | Golgi apparatus membrane ECHIDNA | P: cellular component organization, anatomical structure morphogenesis, transport, cell growth; C: endosome, cytoplasm, membrane, Golgi apparatus | |
| GRMZM5G800853_P01 | DNA-directed RNA polymerase I subunit RPA12-like | F: transferase activity, nucleic acid binding; P: biosynthetic process, nucleobase-containing compound metabolic process; C: nucleolus | |
| GRMZM5G801031_P02 | F1F0-ATPase inhibitor | F: catalytic activity; P: metabolic process; C: membrane | |
| cl12258_2b | UMP CMP kinase 4 | F: nucleotide binding, kinase activity; P: biosynthetic process, nucleobase-containing compound metabolic process; C: nucleus, cytoplasm | |
| GRMZM5G802232_P01 | Unknown |  | |
| GRMZM5G805609_P02 | Glucan endo-1,3-beta-glucosidase 7 precursor | F: carbohydrate binding, hydrolase activity; P: carbohydrate metabolic process; C: cytoplasm, plasma membrane | |
| GRMZM5G807064_P02 | BTB POZ and TAZ domain-containing 3 isoform X1 | F: protein binding, transferase activity; P: abscission, response to abiotic stimulus, flower development, cellular protein modification process, cellular component organization, anatomical structure morphogenesis, biosynthetic process, nucleobase-containing compound metabolic process; C: nucleoplasm | |
| GRMZM5G807260_P01 | Uncharacterized protein LOC100277630 | C: membrane | |
| wrky42 | WRKY DNA-binding domain superfamily | F: DNA binding, transcription factor activity, sequence-specific DNA binding; P: biosynthetic process, nucleobase-containing compound metabolic process; C: intracellular | |
| GRMZM5G806784_P02 | Transmembrane 64 | C: membrane | |
| GRMZM5G812660_P01 | Dna J domain containing | C: membrane | |
| mybr43 | Transcription factor MYB1R1 | F: DNA binding; P: response to endogenous stimulus, biosynthetic process, nucleobase-containing compound metabolic process; C: plastid, nucleus | |
| GRMZM5G814310_P01 | S3 self-incompatibility locus-linked pollen expressed | C: membrane, endoplasmic reticulum | |
| GRMZM5G816609_P01 | 26S proteasome non-ATPase regulatory subunit 2 homolog A-like | F: enzyme regulator activity, hydrolase activity; P: protein metabolic process, catabolic process, cellular process; C: nucleus, cytosol | |
| GRMZM5G817037_P02 | Proteasome subunit beta type-1 | F: hydrolase activity; P: protein metabolic process, generation of precursor metabolites and energy, catabolic process, biosynthetic process, nucleobase-containing compound metabolic process, response to stress, carbohydrate metabolic process, response to biotic stimulus, response to abiotic stimulus, response to external stimulus, lipid metabolic process, cellular component organization; C: cytosol, extracellular region, plasma membrane, plastid, nucleus | |
| ATG3 | Autophagy-related 3 | F: transferase activity; P: cellular component organization, anatomical structure morphogenesis, transport, cell growth; C: cytosol | |
| AY110290 | Palmitoyltransferase ZDHHC9 | F: binding, transferase activity; P: biosynthetic process, cellular process; C: membrane | |
| cl4845_1 | Pre-mRNA processing expressed | C: membrane | |
| GRMZM5G824405_P01 | 6-phosphofructo-2-kinase fructose-2,6-bisphosphatase-like isoform X2 | F: nucleotide binding, carbohydrate binding, kinase activity; P: carbohydrate metabolic process; C: plasma membrane, cytosol | |
| FDH | Alcohol dehydrogenase class-3 | F: catalytic activity, binding; P: metabolic process; C: cytoplasm | |
| GRMZM5G825110_P02 | Phosphatidate cytidylyltransferase 1-like | F: transferase activity; P: lipid metabolic process, biosynthetic process, cellular process; C: membrane, endoplasmic reticulum | |
| GRMZM5G825854_P02 | Conserved oligomeric Golgi complex subunit 3 | F: transporter activity; C: membrane, cytosol, Golgi apparatus | |
| GRMZM5G826174_P01 | TPA: hypothetical protein  ZEAMMB73_528523 | | |
| GRMZM5G826216_P01 | Cell division control | F: hydrolase activity; P: cellular homeostasis, response to biotic stimulus, response to external stimulus, transport; C: cytoplasm, membrane, Golgi apparatus, endosome | |
| GRMZM5G827566_P01 | TPA: hypothetical protein  ZEAMMB73_268437 | | |
| pco151017 | PEROXIN-4 isoform X1 | F: nucleotide binding, protein binding, transferase activity; P: catabolic process, lipid metabolic process, cellular component organization, cellular protein modification process, transport; C: cytoplasm | |
| fat2 | Palmitoyl-acyl carrier, chloroplastic-like | F: hydrolase activity; P: lipid metabolic process, biosynthetic process, cellular process; C: plastid | |
| idh2 | Isocitrate dehydrogenase [NADP] | F: nucleotide binding, catalytic activity; P: generation of precursor metabolites and energy | |
| pco083622 | Transcription factor bHLH69 | F: DNA binding, transcription factor activity, sequence-specific DNA binding, protein binding; P: biosynthetic process, nucleobase-containing compound metabolic process; C: nucleus | |
| pco087396 | CUE domain containing | F: polysaccharide binding; C: chloroplast, membrane, integral component of membrane | |
| GRMZM5G832908_P01 | Galacturonosyl transferase 13 | F: transferase activity; P: carbohydrate metabolic process, cellular component organization, biosynthetic process, nucleobase-containing compound metabolic process; C: cytoplasm, membrane, Golgi apparatus | |
| GRMZM5G833332_P01 | Uncharacterized protein LOC103639541 | C: plastid | |
| rf1-C2-g10 | AMSH-like ubiquitin thioesterase 3 | C: membrane | |
| zim27 | TPA: tify domain CCT motif transcription factor family | F: molecular function; P: signal transduction, P: response to endogenous stimulus, biosynthetic process, nucleobase-containing compound metabolic process, response to stress; C: nucleus | |
| GRMZM5G836939_P02 | LMBR1 domain-containing 2 homolog A-like isoform X1 | C: membrane | |
| GRMZM5G837364_P02 | Methionine aminopeptidase 1A | F: binding, hydrolase activity; P: cellular protein modification process; C: cytosol, ribosome | |
| pco129434 | ROOT HAIR DEFECTIVE 3 | F: nucleotide binding, hydrolase activity; C: membrane, endoplasmic reticulum | |
| GRMZM5G839592_P03 | Sorting nexin 1 | F: lipid binding; P: protein metabolic process, catabolic process, multicellular organism development, biosynthetic process, transport, response to stress, tropism, response to abiotic stimulus, cellular component organization; C: endosome, membrane, cytosol, vacuole | |
| GRMZM5G840013_P03 | Fiber Fb2 |  | |
| GRMZM5G840435_P02 | Folate transporter, chloroplastic | F: structural molecule activity; P: transport, translation; C: membrane, ribosome, plastid | |
| GRMZM5G840909_P01 | Cytidine deaminase | F: binding, hydrolase activity; P: nucleobase-containing compound metabolic process; C: cytosol | |
| GRMZM5G841142_P02 | Thioredoxin reductase 2 | F: catalytic activity; P: nucleobase-containing compound metabolic process, response to stress; C: mitochondria | |
| GRMZM5G841900_P03 | Exosome complex component CSL4 | F: RNA binding; C: nucleus, cytoplasm | |
| GRMZM5G842965_P01 | Negatively light-regulated |  | |
| GRMZM5G844046_P01 | Hypothetical protein ZEAMMB73_317467 |  | |
| GRMZM5G844096_P02 | Alpha-ketoglutarate-dependent dioxygenase alkB | F: catalytic activity; P: DNA metabolic process, response to stress | |
| cl15755_1 | Peptidyl-prolyl cis-trans isomerase G | F: catalytic activity; C: cytoplasm, membrane | |
| GRMZM5G846097_P01 | Hypothetical protein ZEAMMB73_450786, partial | P: cellular component organization, translation; C: membrane, mitochondria | |
| GRMZM5G847159_P02 | Premnaspirodiene oxygenase-like | F: binding, catalytic activity; P: secondary metabolic process, generation of precursor metabolites and energy, biosynthetic process; C: membrane | |
| GRMZM5G848608_P01 | C2 and GRAM domain-  containing At1g03370 | | |
| GRMZM5G848768_P02 | 3-ketoacyl- thiolase peroxisomal | F: transferase activity; P: catabolic process, lipid metabolic process, biosynthetic process, cellular process | |
| GRMZM5G851862_P01 | Cytochrome P450 superfamily | F: binding, catalytic activity; P: secondary metabolic process, biosynthetic process; C: cytoplasm, membrane | |
| GRMZM5G852833_P01 | DUF1644 and RING zinc finger domain | C: mitochondria | |
| iaa34 | Auxin-responsive IAA4 | P: signal transduction, response to endogenous stimulus, biosynthetic process, nucleobase-containing compound metabolic process; C: plastid, nucleus | |
| GRMZM5G853854_P04 | Peroxisomal (S)-2-hydroxy-acid oxidase GLO3 | F: nucleotide binding, catalytic activity; P: photosynthesis, biosynthetic process; C: peroxisome | |
| pco097710 | Peroxisomal fatty acid beta-oxidation multifunctional | F: RNA binding, protein binding, catalytic activity; P: catabolic process, flower development, lipid metabolic process, biosynthetic process, cellular process; C: plastid, peroxisome, cytoskeleton, cell wall | |
| GRMZM5G854655_P01 | Cytokinin-O-glucosyltransferase 3 | F: transferase activity; P: biosynthetic process, cellular process; C: intracellular | |
| GRMZM5G856011_P01 | Cysteine-rich receptor kinase 10 isoform X1 | F: nucleotide binding, kinase activity; P: cellular protein modification process, response to stress; C: plasma membrane, cytoplasm | |
| ckb1 | Casein kinase II subunit beta-like isoform X1 | F: kinase activity, enzyme regulator activity; P: cellular protein modification process; C: intracellular | |
| GRMZM5G858609_P02 | TPA: hypothetical protein  ZEAMMB73_731170 | | |
| GRMZM5G860761_P02 | Glucan endo-1,3-beta-  glucosidase 4 precursor | F: carbohydrate binding, hydrolase activity; P: carbohydrate metabolic process; C: cytoplasm, plasma membrane | |
| GRMZM5G860590_P01 | B2 -like | P: signal transduction | |
| GRMZM5G861269_P01 | Microtubule motor | P: cellular process; C: cytoskeleton | |
| GRMZM5G861300_P01 | Long chain acyl- synthetase 4-like | F: catalytic activity; P: metabolic process; C: plastid | |
| GRMZM5G861603_P01 | ATP-dependent Clp protease ATP-binding subunit clpX | F: nucleotide binding, protein binding, hydrolase activity; P: protein metabolic process, catabolic process, cellular process; C: mitochondria | |
| cl716_1 | Acyl-coenzyme A oxidase peroxisomal-like | F: nucleotide binding, catalytic activity; P: embryo development, catabolic process, post-embryonic development, lipid metabolic process, cellular component organization, transport, reproduction; C: peroxisome | |
| GRMZM5G862317_P01 | GDSL esterase lipase EXL3-like | F: hydrolase activity; C: cytoplasm | |
| GRMZM5G862467_P01 | MOB kinase activator-like 1A | F: kinase activity | |
| GRMZM5G863656_P01 | 40S ribosomal S3a | F: structural molecule activity; P: translation; C: cytosol, ribosome | |
| GRMZM5G864239_P01 | Hypothetical protein ZEAMMB73_059442 |  | |
| GRMZM5G864784_P01 | Hypothetical protein ZEAMMB73_843635 |  | |
| GRMZM5G865298_P01 | NRT1 PTR FAMILY -like | F: transporter activity; C: membrane | |
| GRMZM5G866947_P03 | Ubiquitin-conjugating enzyme E2-17 kDa | F: nucleotide binding, transferase activity; P: catabolic process, cellular protein modification process | |
| GRMZM5G868047_P01 | Vesicle-associated 1-2-like | C: membrane, endoplasmic reticulum | |
| GRMZM5G868683_P02 | F-box LRR-repeat At3g48880 | C: membrane | |
| IDP2367 | 26S proteasome non-ATPase regulatory subunit 13 | F: structural molecule activity; P: protein metabolic process, catabolic process, cellular component organization; C: cytosol, nucleus | |
| GRMZM5G869482_P02 | S3 self-incompatibility locus-linked pollen expressed | C: membrane, endoplasmic reticulum | |
| GRMZM5G871126_P01 | DDT domain-containing DDR4 |  | |
| GRMZM5G871336_P01 | Regulator of G-signaling 7 | C: nucleus, membrane, endoplasmic reticulum | |
| pco108773a | Tripartite motif-containing 5 |  | |
| GRMZM5G872216_P02 | SH3 domain-containing | F: lipid binding; P: cellular component organization; C: plasma membrane | |
| GRMZM5G873287_P01 | Root Hair Defective 3 | F: nucleotide binding, hydrolase activity; C: membrane, endoplasmic reticulum | |
| GRMZM5G876597_P01 | Hypothetical protein ZEAMMB73_587501 |  | |
| GRMZM5G876773_P01 | UPF0664 stress-induced | C: mitochondria, plasma membrane | |
| gpm710 | Eukaryotic initiation factor 5C CG2922- isoform F | F: translation factor activity, RNA binding; C: ribosome, mitochondrion | |
| PRO5 | Profilin | F: protein binding; P: cellular component organization; C: plastid, cytoskeleton | |
| mfs2 | Zinc-induced facilitator-like-1 isoform X1 | F: transporter activity; C: membrane | |
| GRMZM5G877815_P01 | 40S ribosomal S27a | F: structural molecule activity; P: translation; C: ribosome | |
| GRMZM5G877985_P01 | Unknown |  | |
| GRMZM5G878044_P01 | Outer envelope 61 | F: binding, catalytic activity; P: cellular protein modification process transport; C: plastid, membrane, cytosol, endoplasmic reticulum | |
| GRMZM5G878070_P01 | ABC1-like partial | F: nucleotide binding, kinase activity; P: cellular protein modification process; C: plastid | |
| GRMZM5G878139_P01 | 1-acyl-sn-glycerol-3-phosphate acyltransferase PLS1 | F: transferase activity; P: lipid metabolic process, biosynthetic process, cellular process; C: cytoplasm, membrane | |
| GRMZM5G879172_P01 | Uncharacterized protein  LOC100279502 precursor | | |
| GRMZM5G882364_P01 | Uncharacterized protein LOC103627351 | F: binding; P: cellular homeostasis, transport; C: cytoplasm | |
| GRMZM5G882821_P03 | Lipase precursor | F: hydrolase activity; P: lipid metabolic process; C: membrane | |
| GRMZM5G882986_P01 | Fatty-acid-binding 2-like | F: catalytic activity | |
| pco113418 | Negatively light-regulated |  | |
| GRMZM5G884972_P02 | Uncharacterized aarF domain-containing kinase 1 isoform X2 | F: nucleotide binding, kinase activity; P: cellular protein modification process | |
| GRMZM5G886785_P01 | Ran-binding 9 |  | |
| GRMZM5G886096_P01 | Zinc C3HC4 type family expressed | F: binding, transferase activity; P: catabolic process, cellular protein modification process; C: membrane | |
| cl8995_1a | Trafficking particle complex subunit 6B | P: response to endogenous stimulus, transport, pollination | |
| GRMZM5G890241_P01 | Plant intracellular Ras-group-related LRR 7 | C: plasma membrane | |
| GRMZM5G891187_P01 | WAT1-related At3g18200-like | F: transporter activity; C: cytoplasm, plasma membrane | |
| GRMZM5G892025_P02 | GEM 1 | F: nucleic acid binding, nuclease activity | |
| GRMZM5G894156_P01 | Vesicle transport v-SNARE 13 | F: protein binding; P: cellular component organization, transport; C: cytosol, endoplasmic reticulum, Golgi apparatus, vacuole, endosome, membrane | |
| GRMZM5G894233_P02 | Proline transporter 2 isoform X2 | F: transporter activity; C: plasma membrane | |
| GRMZM5G896604_P01 | Hypothetical protein ZEAMMB73_822579 | C: membrane | |
| GRMZM5G897394_P01 | Unknown |  | |
| GRMZM5G897604_P01 | Uncharacterized RING finger -like | F: nucleic acid binding | |
| GRMZM5G897958_P01 | Receptor kinase HERK 1 | F: nucleotide binding, kinase activity; P: cellular protein modification process; C: cytoplasm, membrane | |
| pco111461 | Ataxin-3 homolog | F: hydrolase activity; P: protein metabolic process, biosynthetic process, nucleobase-containing compound metabolic process; C: nucleus | |
| GRMZM5G898668_P01 | Glucuronosyltransferase Os01g0926700 | F: transferase activity; P: cellular component organization; C: Golgi apparatus, cytoplasm, membrane | |
| GRMZM5G898740_P01 | Haloacid dehalogenase-like hydrolase domain-containing 3 | F: hydrolase activity; P: metabolic process; C: mitochondria | |
| GRMZM5G899123_P01 | Aspartic protease oryzasin-1 precursor | F: binding, hydrolase activity; P: protein metabolic process, catabolic process, lipid metabolic process, cellular process; C: lysosome | |
| GRMZM2G041959_P01 | F-box kelch-repeat SKIP11-like | C: membrane | |
| PCO061815 | Uncharacterized protein LOC100194095 | C: cytoplasm, membrane | |
| GRMZM2G042133_P01 | Dna J homolog | F: nucleotide binding, protein binding; P: response to abiotic stimulus, cellular process, response to stress | |
| GRMZM2G042146_P03 | Mitochondrial dicarboxylate tricarboxylate transporter DTC | F: structural molecule activity, transporter activity; P: translation; C: cell wall, mitochondria, membrane, ribosome, vacuole, plastid | |
| GRMZM2G042371_P01 | Elongator complex 2 |  | |
| GRMZM2G042933_P03 | Amino acid permease 7 | F: transporter activity; C: plasma membrane | |
| GRMZM2G043147_P01 | Heat-stress associated 32 | F: catalytic activity; P: response to abiotic stimulus, metabolic process, cellular process, response to stress | |
| cl13451_1 | 3-oxoacyl-[acyl-carrier- ] reductase 4-like | F: nucleotide binding, transferase activity; P: lipid metabolic process, biosynthetic process, cellular process; C: plastid, cytosol | |
| pco137095b | GDSL esterase lipase At1g58430-like | F: hydrolase activity; P: catabolic process, lipid metabolic process | |
| GRMZM2G043799_P01 | Calmodulin-binding receptor-like cytoplasmic kinase 2 | F: nucleotide binding, kinase activity; P: multicellular organism development, cellular protein modification process | |
| GRMZM2G043932_P01 | Heavy metal-associated isoprenylated plant 3-like isoform X1 | F: binding; P: cellular homeostasis, transport; C: cytoplasm | |
| GRMZM2G044011_P01 | PELOTA 1 | F: nuclease activity, binding; P: catabolic process; C: membrane, nucleus, cytoplasm | |
| jmj10 | Lysine-specific demethylase JMJ18-like | C: nucleus, plastid | |
| pco149699b | Sphingosine-1-phosphate lyase | F: binding, catalytic activity; P: protein metabolic process, catabolic process, lipid metabolic process, transport, cellular process, response to stress; C: membrane, endoplasmic reticulum | |
| GRMZM2G044423_P01 | Hypothetical protein ZEAMMB73_653863 |  | |
| CL10251_1 | Endoplasmic reticulum-Golgi intermediate compartment 3-like | C: membrane | |
| GRMZM2G044493_P01 | Unknown |  | |
| cl10175_1 | Rho GTPase-activating 5-like | P: signal transduction, cellular component organization; C: plasma membrane, cell wall | |
| GRMZM2G044805_P01 | Vacuolar sorting-associated 32 homolog 2 | F: kinase activity; P: biosynthetic process, nucleobase-containing compound metabolic process, transport; C: intracellular | |
| GRMZM2G044900_P01 | Seed maturation expressed | C: plastid | |
| GRMZM2G044963_P01 | Calmodulin-7 | F: protein binding, catalytic activity; P: response to biotic stimulus, response to abiotic stimulus, response to external stimulus, signal transduction, post-embryonic development, metabolic process; C: cytosol | |
| GRMZM2G045192_P01 | Polyketide synthase | F: nucleotide binding, catalytic activity; P: response to biotic stimulus, response to external stimulus, response to stress, metabolic process; C: cytosol, plastid | |
| GRMZM2G045239_P01 | Hypothetical protein ZEAMMB73_162762 | P: signal transduction; C: membrane | |
| GRMZM2G045294_P02 | Phospholipase A2 homolog 1 | F: binding, hydrolase activity; P: catabolic process, lipid metabolic process, cellular process; C: extracellular region | |
| GRMZM2G045318_P05 | TPA: hypothetical protein ZEAMMB73_378482, partial | P: transport; C: membrane, endoplasmic reticulum | |
| GRMZM2G045371_P02 | Proteasome subunit beta type-3 | F: hydrolase activity; P: protein metabolic process, catabolic process, cellular process; C: nucleus, cytoplasm | |
| PT3 | Inorganic phosphate transporter 1-4 | F: transporter activity; C: plasma membrane | |
| GRMZM2G045686_P03 | UDP-galactose UDP-glucose transporter 3-like | P: transport; C: membrane | |
| GRMZM2G045714_P01 | Lipase member N | P: lipid metabolic process; C: membrane | |
| GRMZM2G045720_P01 | Eukaryotic peptide chain release factor subunit 1-3 | F: translation factor activity, RNA binding; P: cellular component organization; C: ribosome | |
| GRMZM2G045971_P01 | Transport Sec61 beta subunit | P: transport; C: membrane, endoplasmic reticulum | |
| GRMZM2G046529_P01 | Fatty acid export, chloroplastic-like | C: membrane | |
| GRMZM2G046098_P01 | Like COV 2-like | C: membrane | |
| GRMZM2G046700_P01 | Hexose carrier HEX6 | F: transporter activity; C: plasma membrane | |
| GRMZM2G046583_P01 | Nicotinate phosphoribosyltransferase | F: transferase activity; P: biosynthetic process, nucleobase-containing compound metabolic process | |
| pco095974 | Ferrochelatase- chloroplastic | F: catalytic activity; P: cell death, catabolic process, cellular component organization, biosynthetic process, transport, response to stress; C: plastid, mitochondria, membrane | |
| pco067418a | Mitochondrial fission 1 A | F: transporter activity; P: cellular component organization; C: plastid, membrane | |
| GRMZM2G047607_P01 | Uncharacterized protein LOC100382598 | C: plastid | |
| umc2782 | EI24 homolog | C: membrane | |
| cka2 | Casein kinase II alpha subunit | F: nucleotide binding, transcription factor activity, sequence-specific DNA binding, kinase activity; P: response to abiotic stimulus, post-embryonic development, cellular protein modification process, biosynthetic process, reproduction, nucleobase-containing compound metabolic process; C: plastid, membrane | |
| spds2 | Spermidine synthase | F: transferase activity; P: biosynthetic process, cellular process | |
| pco099200 | Golgi apparatus membrane ECHIDNA | P: cellular component organization, anatomical structure morphogenesis, transport, cell growth; C: endosome, cytoplasm, membrane, Golgi apparatus | |
| GRMZM2G048210_P02 | Serine threonine- kinase CDL1-like | F: nucleotide binding, kinase activity; P: cellular protein modification process; C: plastid | |
| GRMZM2G048549_P01 | EF-Hand containing | F: binding | |
| GRMZM2G048665_P01 | F-box At-B |  | |
| J9009 | Caltractin | F: binding; P: cellular component organization, cell cycle; C: cytoskeleton, cytoplasm | |
| umc2200 | ADP-ribosylation factor 5 | F: nucleotide binding; P: signal transduction; C: intracellular, plasma membrane | |
| GRMZM2G049322_P02 | Uncharacterized protein  LOC100274687  isoform X1 | | |
| cl6408_-2 | Lipo signal peptidase | C: plasma membrane | |
| GRMZM2G050085_P01 | PB1 domain containing | F: transferase activity, carbohydrate binding; P: cellular protein modification process, biosynthetic process; C: membrane | |
| PINY | PIN-LIKES 6-like | P: transport; C: membrane | |
| nas5 | Nicotianamine synthase 3 | F: transferase activity; P: biosynthetic process, transport, cellular process | |
| GRMZM2G050172_P01 | GTP diphosphokinase chloroplastic | F: nucleotide binding, kinase activity, hydrolase activity; P: response to extracellular stimulus, nucleobase-containing compound metabolic process, response to stress; C: plastid, membrane | |
| GRMZM2G050435_P01 | GPI-anchored adhesin  PGA55 isoform X2 | | |
| GRMZM2G050583_P01 | Uncharacterized protein LOC100382548 | C: cytosol | |
| GRMZM2G050641_P07 | Diacylglycerol  O-acyltransferase 1 | F: transferase activity; C: membrane | |
| cl9255_1 | DUF21 domain-containing At2g14520 | C: cytoplasm, membrane | |
| GRMZM2G050701_P02 | Serine threonine- kinase At5g01020 | F: nucleotide binding, RNA binding, kinase activity; P: cellular protein modification process, nucleobase-containing compound metabolic process | |
| GRMZM2G050730_P01 | Pop3 peptide | C: plastid | |
| GRMZM2G050984_P01 | Xanthine dehydrogenase | F: nucleotide binding, catalytic activity; P: catabolic process, nucleobase-containing compound metabolic process; C: cytosol | |
| cko2 | Cytokinin dehydrogenase 4 | F: nucleotide binding, catalytic activity; P: generation of precursor metabolites and energy; C: cytoplasm, extracellular space | |
| GRMZM2G051101_P01 | E3 ubiquitin- ligase SINAT5-like | F: binding, transferase activity; P: cell death, multicellular organism development, catabolic process, cellular protein modification process; C: nucleus, cytoplasm | |
| ERTC | Multi -bridging factor 1c | F: DNA binding, transcription factor activity, sequence-specific DNA binding; P: response to abiotic stimulus, biosynthetic process, nucleobase-containing compound metabolic process, response to stress; C: nucleolus | |
| GRMZM2G051208_P01 | Glycine cleavage system H mitochondrial | F: nucleotide binding; P: catabolic process, cellular process; C: mitochondria | |
| myb95 | R2R3 Myb transcription factor MYB-IF35 | F: DNA binding | |
| GRMZM2G051541_P01 | Phloem-specific lectin | F: carbohydrate binding | |
| FRK2 | Fructokinase-2 | F: nucleotide binding, kinase activity; P: carbohydrate metabolic process, biosynthetic process | |
| GRMZM2G052034_P02 | E3 ubiquitin- ligase MIEL1 | F: protein binding, transferase activity; P: response to biotic stimulus, response to external stimulus, catabolic process, cellular protein modification process, response to stress; C: nucleus, cytoplasm | |
| GRMZM2G051750_P01 | Unknown |  | |
| GRMZM2G052088_P01 | V-type proton ATPase subunit d2 | F: transporter activity; C: membrane | |
| TIDP2896 | RNA recognition motif containing family | F: nucleotide binding, nucleic acid binding | |
| GRMZM2G052471_P01 | PITH domain-containing At3g04780 | F: catalytic activity; P: cellular homeostasis, metabolic process, response to stress; C: cytoplasm | |
| e2f11 | Transcription factor E2F2 | F: DNA binding, transcription factor activity, sequence-specific DNA binding; P: biosynthetic process, nucleobase-containing compound metabolic process; C: nucleus | |
| GRMZM2G052630_P03 | Isovaleryl, mitochondrial | F: nucleotide binding, catalytic activity; P: catabolic process, lipid metabolic process, cellular process; C: membrane, mitochondria | |
| GRMZM2G052713_P01 | AP-2 complex subunit sigma | F: transporter activity; C: mitochondria | |
| Maz56 | Actin-7 | F: nucleotide binding; C: cytoskeleton, cytoplasm | |
| IDP2442 | RER1B-like | P: transport; C: membrane, intracellular | |
| fdx3 | TPA: ferredoxin3 | F: binding; P: generation of precursor metabolites and energy; C: plastid | |
| GRMZM2G053588_P01 | Mediator of RNA polymerase II transcription subunit 13 | F: molecular function; P: biosynthetic process, nucleobase-containing compound metabolic process; C: nucleoplasm, membrane | |
| pco105010 | Acyl- -binding domain-containing 4 | F: binding | |
| GRMZM2G053925_P01 | TOM1 2 | P: transport; C: intracellular | |
| GRMZM2G054193_P01 | Vesicle-associated membrane 721 | F: protein binding; P: cellular component organization, transport; C: cytoplasm, membrane | |
| GRMZM2G054023_P01 | Lectin-like receptor kinase family | F: nucleotide binding, carbohydrate binding, kinase activity; P: cellular protein modification process; C: membrane | |
| GRMZM2G054227_P01 | CASP 5B3 | C: mitochondria, plasma membrane | |
| nac63 | NAC domain-containing 18 | F: DNA binding; P: biosynthetic process, nucleobase-containing compound metabolic process; C: nucleus | |
| apx1 | Ascorbate peroxidase | F: binding, catalytic activity; P: catabolic process, response to stress, cellular process; C: cytoplasm | |
| GRMZM2G054468_P01 | THO complex subunit 4 | F: nucleotide binding, nucleic acid binding | |
| pza01936 | Undecaprenyl pyrophosphate synthetase | F: transferase activity; C: plastid | |
| GRMZM2G055054_P02 | Exocyst complex component SEC15A | P: transport, cellular process; C: cytoplasm | |
| GRMZM2G055970_P01 | Nucleic acid binding | F: nucleic acid binding | |
| TIDP3082 | TPA: hypothetical protein ZEAMMB73_798642 | C: membrane | |
| hsp70-5 | Heat shock cognate 70 kDa 2 | F: nucleotide binding, catalytic activity; P: metabolic process | |
| GRMZM2G056750_P03 | Flocculation FLO11-like isoform X11 | P: cellular component organization; C: cytoskeleton | |
| GRMZM2G056870_P01 | Proteasome subunit alpha type-5 | F: hydrolase activity; P: protein metabolic process, catabolic process, cellular process; C: nucleus, cytoplasm | |
| c3h38 | Zinc finger CCCH domain-containing 14 | F: DNA binding, RNA binding | |
| GRMZM2G057026_P01 | Pre-mRNA-splicing factor SLU7 | F: catalytic activity, binding; P: nucleobase-containing compound metabolic process; C: nucleus, membrane | |
| cl8808_2 | Auxin-responsive IAA6-like | F: protein binding; P: signal transduction, response to endogenous stimulus, biosynthetic process, nucleobase-containing compound metabolic process; C: nucleus | |
| cl1856_4 | Histidine methyltransferase 1 homolog | F: transferase activity; P: response to abiotic stimulus, response to endogenous stimulus, metabolic process, response to stress | |
| pco127416b | Magnesium-dependent phosphatase 1 | F: hydrolase activity; P: metabolic process, cellular process | |
| CCD1 | Carotenoid cleavage dioxygenase | F: catalytic activity; P: catabolic process, cellular process, lipid metabolic process; C: Golgi apparatus, vacuole, plasma membrane | |
| pco100771 | Syntaxin 72 | F: protein binding; P: cellular component organization, transport; C: cytoplasm, membrane | |
| GRMZM2G057283_P01 | Plasma membrane associated | C: cytoplasm, membrane | |
| mybr77 | DNA binding | F: DNA binding; P: biosynthetic process, nucleobase-containing compound metabolic process; C: nucleus | |
| jmj14 | Lysine-specific demethylase JMJ706 | F: binding, catalytic activity; P: flower development, cellular component organization, cellular protein modification process, anatomical structure morphogenesis, biosynthetic process, nucleobase-containing compound metabolic process, regulation of gene expression, epigenetic; C: nucleus | |
| GRMZM2G057475_P04 | Sphingosine kinase 1-like | F: kinase activity | |
| GRMZM2G057491_P01 | Glutamyl-tRNA cytoplasmic | F: nucleotide binding, RNA binding, catalytic activity; P: translation, nucleobase-containing compound metabolic process; C: mitochondria, cytosol, plastid | |
| GRMZM2G057674_P01 | Hypothetical protein  isoform X1 | | |
| GRMZM2G057733_P01 | GABA transporter 2 | F: transporter activity; C: plasma membrane | |
| GRMZM2G057611_P01 | NRT1 PTR family | F: transporter activity; P: cellular component organization, cell cycle; C: nuclear envelope, cytoplasm, membrane | |
| ald1 | Fructose-bisphosphate aldolase cytoplasmic isozyme | F: catalytic activity; P: carbohydrate metabolic process, generation of precursor metabolites and energy, catabolic process, biosynthetic process, nucleobase-containing compound metabolic process; C: cytoplasm | |
| GRMZM2G057910_P01 | Scyllo-inositol 2-dehydrogenase (NADP(+)) | F: catalytic activity; P: metabolic process; C: cytosol | |
| pco080186 | Serine hydroxymethyltransferase | C: membrane | |
| cl862_1 | Polyadenylate-binding RBP45-like | F: nucleotide binding, nucleic acid binding | |
| sod4A | Superoxide dismutase 2 | F: binding, catalytic activity; P: metabolic process, response to stress, cellular process; C: cytoplasm | |
| fnr1 | Ferredoxin--NADP root chloroplastic | F: nucleotide binding, catalytic activity; P: generation of precursor metabolites and energy, photosynthesis; C: plastid | |
| TIDP3061 | Zinc finger A20 and AN1 domain-containing stress-associated 8 | F: DNA binding; P: response to abiotic stimulus, post-embryonic development, cellular component organization, cell cycle, reproduction | |
| pco113424 | Accelerated cell death 11 | F: lipid binding, transporter activity; P: response to biotic stimulus, cell death, response to external stimulus, response to stress; C: cytoplasm | |
| GRMZM2G058900_P05 | Cell division control 50 | P: cellular process; C: membrane | |
| GRMZM2G059073_P02 | NAD kinase 1 | F: nucleotide binding, kinase activity; P: biosynthetic process, nucleobase-containing compound metabolic process | |
| pco124263 | Transmembrane 18 | C: membrane | |
| dsc3 | ADP-ribosylation factor GTPase-activating AGD3 | F: nucleic acid binding, enzyme regulator activity; C: cytoplasm | |
| GRMZM2G059381_P01 | Long chain acyl- synthetase peroxisomal-like isoform X1 | F: catalytic activity; P: metabolic process | |
| GRMZM2G059618_P01 | Nuclear nucleic acid-binding C1D |  | |
| bzip91 | bZIP transcription factor 17 | F: DNA binding, transcription factor activity, sequence-specific DNA binding, transferase activity; P: biosynthetic process, nucleobase-containing compound metabolic process; C: intracellular, membrane | |
| GRMZM2G060118_P01 | PIFa transposase | C: membrane | |
| cpp4 | CXC domain containing TSO1 1 | C: plastid | |
| GRMZM2G060190_P01 | Uncharacterized protein LOC103632822 | C: cytoplasm, membrane | |
| ADF3 | Actin-depolymerizing factor 3 | F: protein binding; P: cellular component organization, response to stress; C: cytoskeleton, cytoplasm | |
| GRMZM2G060762_P01 | Major facilitator superfamily domain-containing 12-like | P: transport; C: membrane | |
| GRMZM2G060800_P01 | Aldehyde dehydrogenase family 3 member H1 | F: catalytic activity; P: carbohydrate metabolic process, P: generation of precursor metabolites and energy, catabolic process, lipid metabolic process, biosynthetic process, nucleobase-containing compound metabolic process; C: membrane | |
| GRMZM2G061096_P01 | Transmembrane 9 superfamily member 3 | C: cytoplasm, membrane | |
| GRMZM2G061232_P01 | TPA: hypothetical protein  ZEAMMB73_197601 | | |
| umc2605 | Ceramide  glucosyltransferase | F: transferase activity; P: lipid metabolic process, biosynthetic process, cellular process; C: membrane | |
| TIDP3725 | Syntaxin-61-like | F: protein binding; P: cellular component organization, transport; C: cytoplasm, Golgi apparatus, membrane | |
| Pti1b | Pto kinase interactor 1 | F: nucleotide binding, kinase activity; P: cellular protein modification process | |
| hypro4 | DNA-dependent metalloprotease WSS1 | F: binding | |
| pco082032 | 26S proteasome non-ATPase regulatory subunit 7 homolog A | P: protein metabolic process, catabolic process, cellular process; C: intracellular | |
| umc2257 | CWC15 homolog | P: nucleobase-containing compound metabolic process; C: nucleus, membrane | |
| GRMZM2G062151_P01 | Cytochrome P450 86A2-like | F: binding, catalytic activity; P: metabolic process; C: membrane | |
| umc1314 | Ras-related RGP2 | F: nucleotide binding, DNA binding; P: signal transduction, transport; C: intracellular, plasma membrane | |
| umc1140 | Tubby-like F-box 1 | F: lipid binding; P: biological process; C: cell | |
| GRMZM2G062156_P02 | Polyol transporter 5-like | F: transporter activity; C: cytoplasm, plasma membrane | |
| GRMZM2G062390_P01 | Peroxidase 46-like | F: binding, catalytic activity; P: catabolic process, cellular component organization, response to stress; C: cell wall, extracellular region | |
| GRMZM2G062394_P01 | PP2Ac-2 - Phosphatase 2A isoform 2 belonging to family 2 | F: binding, hydrolase activity; P: cellular protein modification process; C: cytoplasm | |
| pco139673a | Lactation elevated 1-like | F: nucleotide binding; C: mitochondria | |
| GRMZM2G062476_P02 | Myeloid leukemia factor 1-like isoform X1 | C: plasma membrane | |
| GRMZM2G062632_P01 | 2-oxoglutarate-dependent dioxygenase AOP1 | F: binding; catalytic activity; P: metabolic process | |
| GRMZM2G062673_P01 | Calcium-binding CML22 | F: binding; P: carbohydrate metabolic process, catabolic process; C: plastid | |
| cl14031_1 | BRCA1-associated RING domain 1 | C: membrane, cytosol | |
| mpk14 | Mitogen-activated kinase 3 | F: nucleotide binding, signal transducer activity, kinase activity; P: cellular protein modification process; C: intracellular | |
| GRMZM2G063069_P01 | Serine threonine- kinase HT1-like | F: nucleotide binding, kinase activity; P: cellular protein modification process | |
| GRMZM2G063291_P01 | One cut domain family member 2-like | F: carbohydrate binding, galactosyltransferase activity; P: protein glycosylation; C: mitochondria, membrane | |
| e2f6 | Transcription factor DPB | F: DNA binding, transcription factor activity, sequence-specific DNA binding; P: biosynthetic process, nucleobase-containing compound metabolic process, cell cycle; C: nucleus | |
| pco146901 | Bet1 At4g14600 | C: membrane | |
| umc2313 | RNA-binding -like | F: nucleotide binding, nucleic acid binding, transferase activity | |
| GRMZM2G063931_P01 | SUMO-conjugating enzyme SCE1 | F: nucleotide binding, protein binding, transferase activity; P: cellular protein modification process; C: nucleus, cytoplasm | |
| pco126351 | Serine threonine- kinase SAPK4 | F: nucleotide binding, kinase activity; P: signal transduction, response to endogenous stimulus, cellular protein modification process; C: nucleus, cytoplasm | |
| Atg4a | Cysteine protease ATG4B isoform X1 | F: protein binding, hydrolase activity; P: carbohydrate metabolic process, cellular component organization, cellular protein modification process, biosynthetic process, transport; C: vacuole, cytosol | |
| chb102 | SWI SNF complex subunit SWI3B | F: DNA binding; P: response to abiotic stimulus, post-embryonic development, cellular component organization, regulation of gene expression_epigenetic, reproduction; C: plastid, nucleus | |
| umc1796 | Zinc transporter 4 | F: transporter activity; C: membrane | |
| caat1 | Nuclear transcription factor Y subunit B | F: DNA binding, protein binding; P: biosynthetic process, nucleobase-containing compound metabolic process; C: nucleus | |
| GRMZM2G064695_P03 | Succinyl- ligase [ADP-forming] subunit mitochondrial | F: nucleotide binding, catalytic activity; P: generation of precursor metabolites and energy; C: mitochondria | |
| pco134887 | Ribonuclease NGR2 | F: RNA binding, nuclease activity; C: cytoplasm, membrane | |
| GRMZM2G064949_P01 | Acyl- thioesterase 1 | F: hydrolase activity; P: catabolic process, lipid metabolic process, cellular protein modification process; C: cytoplasm | |
| GRMZM2G064960_P01 | Outer envelope membrane 7 | C: membrane | |
| GRMZM2G065694_P01 | DeSI At4g17486 |  | |
| GRMZM2G065757_P01 | Aspartic protease oryzasin-1 precursor | F: hydrolase activity; P: protein metabolic process, catabolic process, lipid metabolic process; C: vacuole | |
| GRMZM2G066024_P01 | Fructose-bisphosphate aldolase cytoplasmic isozyme | F: catalytic activity; P: carbohydrate metabolic process, generation of precursor metabolites and energy, catabolic process, biosynthetic process, nucleobase-containing compound metabolic process; C: cytoplasm | |
| GRMZM2G064993_P01 | Annexin D1 | F: lipid binding | |
| GRMZM2G065097_P01 | UTP:RNA uridylyltransferase 1 | F: transferase activity | |
| GRMZM2G066153_P01 | Phosphoribosylanthranilate transferase | F: lipid binding, transferase activity; C: plasma membrane | |
| GRMZM2G066441_P01 | Cytochrome P450 family expressed | F: binding, catalytic activity; P: generation of precursor metabolites and energy; C: membrane, plastid | |
| GRMZM2G066612_P01 | Thioredoxin h | F: catalytic activity; P: cellular homeostasis, cell communication, response to stress, metabolic process; C: cytosol, plasma membrane, plastid, nucleus | |
| cl28155_-2 | CTD small phosphatase | F: hydrolase activity; P: cellular protein modification process; C: membrane | |
| PDIL5-4 | Disulfide isomerase-like 5-4 | F: nucleic acid binding, catalytic activity; P: cellular homeostasis; C: membrane, cell | |
| GRMZM2G067417_P01 | Cytochrome c oxidase subunit 6b-2 | F: catalytic activity, transporter activity; P: generation of precursor metabolites and energy, nucleobase-containing compound metabolic process; C: mitochondria, membrane | |
| CaM2 | Calmodulin | F: binding, kinase activity; P: signal transduction, cellular protein modification process; C: cytoplasm | |
| GRMZM2G067583_P01 | Tat pathway signal sequence family | C: membrane | |
| phm15278 | Cytochrome b5 | F: binding; P: generation of precursor metabolites and energy; C: membrane, endoplasmic reticulum | |
| AC149818.2_FGP001 | Ras-related Rab-18 | F: nucleotide binding; P: signal transduction; C: cytoplasm | |
| AC149818.2_FGP006 | Ubiquitin-conjugating enzyme E2 4-like | F: nucleotide binding, protein binding, transferase activity; P: catabolic process, cellular protein modification process; C: cytoplasm | |
| pco092990a | F-box kelch-repeat At1g74510 |  | |
| MYO1 | Myosin 1 | F: nucleotide binding, protein binding, carbohydrate binding, motor activity; P: carbohydrate metabolic process; C: plasma membrane, cytoskeleton | |
| GRMZM2G067747_P01 | Sodium hydrogen exchanger 6-like | F: transporter activity; C: membrane | |
| AC155624.2_FGP006 | Phosphatase 2C | F: binding, hydrolase activity; P: cellular protein modification process; C: cell | |
| IDP871 | Ras-related RABH1e | F: nucleotide binding; P: signal transduction, transport; C: cytosol, Golgi apparatus | |
| pco108652 | F-box LRR-repeat At3g28410 | C: mitochondria | |
| AC182482.3_FGP003 | Metal tolerance 7-like | F: transporter activity; P: cellular homeostasis; C: membrane, vacuole | |
| pco120447 | Reticulon B2 | C: membrane, endoplasmic reticulum | |
| AC194039.4_FGP009 | TPA: hypothetical protein ZEAMMB73_619464 | C: membrane, integral component of membrane | |
| AC194425.3_FGP005 | Serine threonine- kinase yrzF | F: kinase activity; C: mitochondria | |
| AC196426.3_FGP007 | Root Hair Defective 3 | F: nucleotide binding, hydrolase activity; C: membrane, endoplasmic reticulum | |
| AC199315.4_FGP001 | Transport Sec61 beta subunit | P: transport; C: endoplasmic reticulum, membrane | |
| pco094838 | ATPase ASNA1 homolog | F: nucleotide binding, hydrolase activity; P: protein metabolic process, catabolic process, cellular component organization, transport, response to stress; C: endoplasmic reticulum | |
| pco130633 | Hydroxymethylglutaryl- synthase | F: transferase activity; P: catabolic process, lipid metabolic process, biosynthetic process, cellular process | |
| AC203862.4_FGP001 | Vacuolar processing enzyme 4 | F: hydrolase activity; protein metabolic process, catabolic process, cellular process; C: vacuole | |
| PRMS | Pathogenesis-related maize seed | P: response to biotic stimulus, response to stress; C: extracellular region, cytoplasm | |
| glossy8 | Very-long-chain 3-oxoacyl- reductase 1-like | C: membrane | |
| AC208897.3_FGP004 | Sugar transport 13 | F: transporter activity; C: plasma membrane | |
| AC209374.4_FGP002 | 2-C-methyl-D-erythritol 2,4-cyclodiphosphate, chloroplastic | F: catalytic activity, binding; P: lipid metabolic process, biosynthetic process, cellular process; C: plastid | |
| IPT5 | Adenylate isopentenyltransferase chloroplastic-like | F: protein binding, transferase activity; P: nucleobase-containing compound metabolic process; C: plastid, mitochondria | |
| pco076336b | Seleno K | C: mitochondria, membrane | |
| AC211394.4_FGP004 | Phosphoenolpyruvate phosphatase | F: binding, hydrolase activity; P: response to abiotic stimulus, response to stress, cellular protein modification process; C: cytosol, vacuole, cell wall | |
| AC212835.3_FGP008 | Receptor kinase At5g47070 | F: nucleotide binding, kinase activity; P: cellular protein modification process | |
| AC213884.3_FGP001 | Ran-binding 1 | F: protein binding, enzyme regulator activity; P: protein metabolic process, catabolic process, cellular component organization, transport, cell cycle; C: nucleus, cytoskeleton, cytoplasm | |
| cl34400_1a | F-box kelch-repeat At1g22040-like |  | |
| pco076711 | Ankyrin repeat domain-containing 2 |  | |
| csu333 | Stearoyl-[acyl-carrier- ] 9-desaturase, chloroplastic | F: binding, catalytic activity; P: lipid metabolic process, biosynthetic process, cellular process; C: plastid | |
| AC149475.2_FGP002 | Nicotiana lesion-inducing like | C: cytoplasm, membrane | |
| AC149475.2_FGP003 | Proteasome subunit alpha type-5 | F: hydrolase activity; P: protein metabolic process, catabolic process, cellular process; C: nucleus, cytoplasm | |
| cl27369_1 | Diphthamide biosynthesis 2 | P: cellular protein modification process, biosynthetic process | |
| cl4777_1c | AIG1 | F: nucleotide binding | |
| AC217840.3_FGP001 | UPF0548 At2g17695 isoform X1 | F: hydrolase activity; P: protein metabolic process; C: plastid | |
| PDK2 | Pyruvate dehydrogenase (acetyl-transferring) mitochondrial-like | F: nucleotide binding, receptor activity, kinase activity, signal transducer activity; C: cellular component | |
| pco094349 | Uncharacterized protein LOC100274892 |  | |
| AC149828.2_FGP002 | Proteasome subunit alpha type-6 | F: hydrolase activity; P: protein metabolic process, catabolic process, cellular process; C: nucleus, cytoplasm | |
| pco142662 | 26S proteasome non-ATPase regulatory subunit 11 homolog | C: cytosol | |
| AC217050.4_FGP006 | 14-3-3-like protein | F: protein binding; C: nucleus, cytoplasm | |
| TIDP3322 | Uncharacterized membrane | C: membrane | |
| AC207347.3_FGP005 | Copper transporter | F: transporter activity; C: membrane | |
| AC207265.3_FGP002 | Clathrin assembly At5g35200 | F: protein binding, lipid binding; P: cellular component organization; C: cytoplasm | |
| AC206989.4_FGP002 | E3 ubiquitin- ligase ARI8 | F: protein binding, transferase activity; P: catabolic process, cellular protein modification process; C: mitochondria | |
| AC225147.4_FGP002 | 40S ribosomal S23 | F: structural molecule activity; P: translation; C: cytosol, ribosome, membrane | |
| AC226235.2_FGP003 | Swi5-dependent recombination DNA repair 1 homolog | C: plastid | |
| AC233910.1_FGP010 | SRSF kinase 1 | F: nucleotide binding, kinase activity; P: cellular protein modification process; | |
| AC233863.1_FGP002 | Glutamine Dumper 6 | C: cytoplasm, membrane | |
| AC233942.1_FGP001 | F-box domain containing | C: membrane | |
| AC233979.1_FGP008 | Uncharacterized protein LOC100502301 |  | |
| AC234154.1_FGP008 | Phospholipase A(1) partial | F: transferase activity; P: lipid metabolic process, biosynthetic process, cellular process; C: mitochondria | |
| AC235543.1_FGP002 | Anion transporter, chloroplastic | F: transporter activity; C: membrane, plastid | |
| GRMZM2G000014_P04 | RING zinc finger domain superfamily | F: transferase activity, binding; P: catabolic process, cellular protein modification process, cell cycle; C: nucleus | |
| pco073802a | E3 ubiquitin- ligase HIP1 | F: binding | |
| GRMZM2G000581_P01 | Glucuronosyltransferase Os01g0926700 | F: transferase activity P: cellular component organization; C: Golgi apparatus, cytoplasm, membrane | |
| GRMZM2G000718_P01 | Novel plant SNARE 13 | F: protein binding; P: cellular component organization, transport; C: cytosol, endoplasmic reticulum, Golgi apparatus, vacuole, endosome, membrane | |
| GRMZM2G000812_P01 | Bidirectional sugar transporter SWEET4 | F: transporter activity; C: plasma membrane | |
| GRMZM2G000823_P01 | DEAD-box ATP-dependent RNA helicase 38 | F: nucleotide binding, RNA binding, hydrolase activity; P: response to abiotic stimulus, response to endogenous stimulus, nucleobase-containing compound metabolic process, transport; response to stress, translation; C: nuclear envelope, cytoplasm, plasma membrane | |
| GRMZM2G001097_P01 | TNF receptor-associated factor family DDB_G0290965 isoform X1 | F: binding, transferase activity; P: nucleobase-containing compound metabolic process; C: nucleus, mitochondria, cytosol | |
| GRMZM2G001255_P01 | TPA: hypothetical protein ZEAMMB73_674865 | C: vacuole, plastid | |
| GRMZM2G001265_P01 | S-acyltransferase 14 | F: binding, transferase activity; P: biosynthetic process, cellular process; C: membrane | |
| GRMZM2G001639_P01 | F-box PP2-A13 |  | |
| GRMZM2G001820_P01 | Uncharacterized protein LOC100304339 | F: catalytic activity; C: membrane | |
| pco061337 | Methylmalonate-semialdehyde dehydrogenase [acylating] mitochondrial | F: binding, catalytic activity; P: carbohydrate metabolic process, generation of precursor metabolites and energy, catabolic process, lipid metabolic process, biosynthetic process, response to stress, nucleobase-containing compound metabolic process; C: plastid, membrane, mitochondria | |
| ROP1 | Rac-like GTP-binding 3 | F: nucleotide binding, protein binding; P: signal transduction, membrane, cytoplasm | |
| MPK7 | Mitogen-activated kinase 1 | F: nucleotide binding, signal transducer activity, kinase activity; P: cellular protein modification process, response to stress; C: intracellular | |
| cl17205_1 | Ubiquitin- ligase | F: catalytic activity | |
| GRMZM2G002786_P01 | Reticulon B1 | C: membrane, endoplasmic reticulum | |
| GRMZM2G002825_P01 | Actin-depolymerizing factor 3 | F: protein binding; P: cellular component organization, response to stress; C: cytoskeleton, cytoplasm | |
| GRMZM2G003028_P02 | Thioredoxin-like 3- chloroplastic | F: binding, catalytic activity; P: cellular homeostasis, metabolic process, transport, response to stress; C: plastid | |
| TIDP2770 | Tobamovirus multiplication 3 | C: membrane | |
| GRMZM2G003108_P01 | CRAL TRIO domain containing | C: membrane | |
| GRMZM2G003642_P01 | Mitochondrial pyruvate carrier 4-like | P: transport; C: mitochondria, membrane | |
| GRMZM2G003718_P01 | Alpha beta hydrolase domain-containing 17C-like | F: hydrolase activity; P: protein metabolic process; C: plastid | |
| GRMZM2G003789_P01 | IAA-amino acid hydrolase ILR1-like 9 | F: hydrolase activity; P: metabolic process; C: cytoplasm | |
| GRMZM2G003754_P01 | Uncharacterized protein LOC100192095 precursor | C: cytoplasm, membrane | |
| GRMZM2G003853_P01 | 3-beta-hydroxysteroid-Delta(8),Delta(7)-isomerase | F: catalytic activity; P: lipid metabolic process, regulation of gene expression_ epigenetic, translation; C: endoplasmic reticulum, plasma membrane | |
| GRMZM2G003937_P02 | TPA: proline-rich family |  | |
| pco141112 | Cell division control 50 | P: cellular process; C: membrane, plastid | |
| GRMZM2G004119_P01 | RING zinc finger domain superfamily | F: binding | |
| TIDP3048 | 54S ribosomal mitochondrial | F: structural molecule activity; P: biological process; C: mitochondria, ribosome | |
| pco082817 | Reticulon B1 | C: membrane, endoplasmic reticulum | |
| GRMZM2G004382_P01 | Ketol-acid, chloroplastic | F: nucleotide binding, protein binding, catalytic activity; P: biosynthetic process, cellular process; C: plastid | |
| pco148373a | Uncharacterized protein LOC103632029 | P: transport; C: nucleus, membrane | |
| MKK3 | Mitogen-activated kinase / kinase 3 | F: nucleotide binding, signal transducer activity, kinase activity; P: response to biotic stimulus, response to abiotic stimulus, response to external stimulus, cellular protein modification process, response to stress; C: cytoplasm | |
| GRMZM2G004516_P01 | VAN3-binding -like |  | |
| nac79 | NAC domain transcription factor superfamily isoform X2 | F: DNA binding; P: biosynthetic process, nucleobase-containing compound metabolic process; C: nucleus, membrane | |
| GRMZM2G004694_P01 | Polyol transporter 5-like | F: transporter activity; C: cytoplasm, plasma membrane | |
| GRMZM2G004748_P01 | ABC transporter B family member 2-like | F: nucleotide binding, transporter activity, hydrolase activity; C: membrane | |
| GRMZM2G004858_P01 | UDP-glycosyltransferase 87A1-like | F: transferase activity; P: biosynthetic process, cellular process; C: intracellular | |
| pco100001 | Vacuolar sorting-associated 2 homolog 3 | P: transport; C: endosome, membrane, vacuole | |
| Atg7 | Ubiquitin-like modifier-activating enzyme atg7 | F: nucleic acid binding, transferase activity; P: cell communication, response to extracellular stimulus, multicellular organism development, catabolic process, cellular protein modification process, cellular component organization, biosynthetic process, response to stress; C: cytosol | |
| GRMZM2G005374_P01 | VAMP At1g33475 | F: nucleotide binding, nucleic acid binding; C: membrane | |
| GRMZM2G005583_P01 | Methylesterase chloroplastic | F: hydrolase activity | |
| GRMZM2G005749_P01 | Uncharacterized protein LOC100276333 | C: mitochondria | |
| GRMZM2G005865_P01 | F-box SKIP14 | C: cytoplasmic vesicle | |
| GRMZM2G006117_P01 | Transmembrane 9 superfamily member 8 | C: cytoplasm, membrane | |
| GRMZM2G006704_P01 | Cytochrome c oxidase subunit 5C | F: catalytic activity, transporter activity; P: generation of precursor metabolites and energy, nucleobase-containing compound metabolic process; C: membrane, mitochondria | |
| GRMZM2G006948_P01 | Uncharacterized protein LOC103641749 | C: plastid | |
| GRMZM2G007120_P01 | Glutathione-specific gamma-glutamylcyclotransferase 2 | C: cytosol | |
| cl6208_1 | Hypothetical protein ZEAMMB73_086017 |  | |
| GRMZM2G007025_P01 | Domain containing expressed |  | |
| GRMZM2G007157_P01 | Lecithin-cholesterol acyltransferase-like 4 | F: transferase activity, hydrolase activity; P: catabolic process, lipid metabolic process, biosynthetic process, cellular process; C: cytosol | |
| GRMZM2G007258_P03 | Hypothetical protein isoform X1 | P: post-embryonic development, reproduction | |
| UCE | Ubiquitin-conjugating enzyme E2 7 | F: nucleotide binding, protein binding, transferase activity; P: cellular protein modification process; C: cytoplasm | |
| GRMZM2G007404_P03 | UDP-glucuronic acid decarboxylase 2-like | C: membrane | |
| GRMZM2G007481_P01 | PIN-LIKES 3 | P: transport; C: cytoplasm, membrane | |
| umc2219 | Dehydrodolichyl diphosphate synthase 6 | F: transferase activity | |
| GRMZM2G008095_P01 | transport SFT2 | P: transport; C: membrane | |
| GRMZM2G008539_P01 | UDP-glycosyltransferase 708A6-like | F: transferase activity; P: biosynthetic process, cellular process; C: membrane, intracellular | |
| GRMZM2G008607_P01 | Uncharacterized conserved expressed | C: mitochondria | |
| GRMZM2G009139_P01 | UPF0481 At3g02645 | C: membrane | |
| GRMZM2G009538_P01 | Acidic leucine-rich nuclear  phospho 32-related 1-like | | |
| GRMZM2G009591_P02 | ATP-dependent 6-phosphofructokinase,  chloroplastic | F: nucleotide binding, kinase activity; P: carbohydrate metabolic process, generation of precursor metabolites and energy, catabolic process, biosynthetic process, nucleobase-containing compound metabolic process; C: cytosol | |
| GRMZM2G009724_P01 | Speckle-type POZ |  | |
| GRMZM2G009888_P01 | Acyl-coenzyme A thioesterase 13 | C: peroxisome | |
| pco090181a | Transmembrane emp24 domain-containing p24beta3 | P: transport; C: membrane, endoplasmic reticulum, Golgi apparatus, vacuole | |
| IDP532 | Small multi-drug export | C: membrane, plastid | |
| GRMZM2G009940_P02 | Short-chain dehydrogenase TIC, chloroplastic | C: mitochondria | |
| GRMZM2G010065_P01 | RNA polymerase II subunit 5-mediating homolog | F: chromatin binding, enzyme regulator activity; P: multicellular organism development, metabolic process, reproduction, cellular process; C: nucleoplasm | |
| cl11096_1 | Uncharacterized protein LOC100272345 isoform X1 | P: metabolic process, cellular process | |
| GRMZM2G010235_P01 | Lipopolysaccharide-induced tumor necrosis factor-alpha factor homolog | P: cell death, response to stress; C: plasma membrane | |
| GRMZM2G010406_P01 | Argininosuccinate, chloroplastic | F: nucleotide binding, catalytic activity; P: catabolic process, biosynthetic process, nucleobase-containing compound metabolic process; C: cytoplasm | |
| GRMZM2G010422_P01 | Glucose-induced degradation 4 homolog | P: lipid metabolic process, biosynthetic process, cellular process | |
| pco106335 | Fumarate hydratase mitochondrial | F: catalytic activity; P: generation of precursor metabolites and energy, cellular component organization; C: cytosol, mitochondria | |
| cc4 | Cysteine ase inhibitor 12 | F: enzyme regulator activity; P: protein metabolic process, response to stress, cellular process: C: membrane, extracellular region | |
| GRMZM2G013481_P01 | Lysine ketoglutarate reductase trans-splicing related 1-like | C: membrane | |
| GRMZM2G013619_P01 | Polyadenylate-binding 2-like | F: nucleotide binding, RNA binding; C: cytoplasm | |
| GRMZM2G013625_P04 | AMSH-like ubiquitin thioesterase 2 isoform X1 | F: RNA binding | |
| GRMZM2G013639_P01 | Pre-mRNA 3 -end-processing factor FIP1 | F: binding; C: endosome, cytoplasm, membrane, Golgi apparatus | |
| GRMZM2G013704_P01 | Uncharacterized protein LOC100276462 |  | |
| pco111061 | 26S proteasome non-ATPase regulatory subunit 12 homolog A-like | P: protein metabolic process, catabolic process, cellular process; C: nucleus, cytoplasm, membrane | |
| HMGc1 | HMG1 2 | F: nucleotide binding, kinase activity; P: cellular protein modification process | |
| GRMZM2G014004_P01 | Plant UBX domain-containing 11 |  | |
| GRMZM2G014066_P01 | Fiber Fb2 |  | |
| GRMZM2G014136_P01 | Acyl-coenzyme A oxidase peroxisomal-like | F: nucleotide binding, catalytic activity; P: catabolic process, lipid metabolic process, cellular process; C: peroxisome, membrane | |
| pco096827 | TPA: homeodomain-like transcription factor superfamily | C: mitochondria | |
| GRMZM2G014454_P01 | Metal tolerance 5 | F: transporter activity; P: cellular homeostasis; C: endosome, membrane, Golgi apparatus, vacuole | |
| nac109 | NAC domain-containing 48 | F: DNA binding; P: biosynthetic process, nucleobase-containing compound metabolic process; C: nucleus | |
| GRMZM2G014672_P01 | BI1 | C: mitochondria, membrane | |
| pco063318 | Prefoldin subunit 5 | F: protein binding; P: cellular process; C: cytosol | |
| cl37957_1 | Hydrophobic LTI6B | C: cytoplasm, membrane | |
| GRMZM2G015767_P01 | NRT1 PTR FAMILY -like | F: transporter activity; C: membrane | |
| pco143033 | Cystinosin homolog isoform X1 | F: transporter activity; C: membrane, vacuole | |
| bhlh174 | Transcription factor bHLH30-like | F: DNA binding, protein binding; C: nucleus | |
| GRMZM2G016153_P01 | Uncharacterized protein LOC103635650 | C: membrane, plastid | |
| GRMZM2G016275_P01 | Nucleic acid binding | C: mitochondria | |
| alf5 | Nucleic acid binding | F: DNA binding, protein binding; P: biosynthetic process, nucleobase-containing compound metabolic process; C: nucleus, membrane | |
| GLU1 | Non-cyanogenic beta-glucosidase precursor | F: hydrolase activity; P: carbohydrate metabolic process, signal transduction, response to endogenous stimulus; C: plastid | |
| GRMZM2G016892_P01 | ER membrane complex subunit 2-A-like |  | |
| GRMZM2G011169_P01 | Embryogenesis-associated EMB8 | C: membrane | |
| GRMZM2G011513_P01 | Elicitor-responsive 3 |  | |
| GRMZM2G011559_P01 | Alpha-soluble NSF attachment | F: protein binding; P: cellular component organization, transport; C: vacuole, membrane | |
| nac44 | NAC transcription factor 29 | F: DNA binding; P: biosynthetic process, nucleobase-containing compound metabolic process; C: nucleus | |
| GRMZM2G011636_P02 | kDa vesicle transport | F: protein binding; P: cellular component organization, transport; C: cytoplasm, membrane | |
| GRMZM2G017305_P01 | Katanin p60 ATPase-containing subunit A1 | F: nucleotide binding, protein binding, hydrolase activity; P: cellular component organization; C: nucleus, cytoskeleton, cytoplasm | |
| GRMZM2G017329_P01 | Syntaxin 23 | F: protein binding; P: cellular component organization, transport; C: cytoplasm, membrane | |
| pco129008 | Uncharacterized protein LOC100276806 precursor | C: cytoplasm, membrane | |
| GRMZM2G017966_P02 | Translation factor SUI1 | F: nucleotide binding, translation factor activity, RNA binding; P: signal transduction; C: ribosome | |
| GRMZM2G018006_P01 | Aminotransferase ACS12 | F: binding, transferase activity; P: biosynthetic process, cellular process; C: membrane | |
| GRMZM2G018103_P01 | Serine incorporator | F: transporter activity; C: membrane, plastid | |
| GRMZM2G018126_P01 | Transmembrane 9 superfamily member 4 | C: cytoplasm, membrane | |
| GRMZM2G018251_P02 | WW domain-containing oxidoreductase | F: catalytic activity; P: metabolic process | |
| pco095978 | Ethylene-responsive transcription factor 1 | F: DNA binding, transcription factor activity, sequence-specific DNA binding; P: biosynthetic process, nucleobase-containing compound metabolic process; C: nucleus | |
| pza03057 | Ras-related RIC2 | F: nucleotide binding, DNA binding; P: signal transduction, transport; C: intracellular, plasma membrane | |
| GRMZM2G018775_P01 | SMG7 | P: catabolic process, cellular component organization, nucleobase-containing compound metabolic process, cell cycle, reproduction; C: nucleus, cytoplasm | |
| umc2196 | Cornichon homolog 1-like | P: transport; C: cytoplasm, membrane | |
| ereb180 | Root abundant factor | F: DNA binding, transcription factor activity, sequence-specific DNA binding; P: biosynthetic process, nucleobase-containing compound metabolic process; C: plastid, nucleus | |
| gpm518 | Aspartate-tRNA ligase, cytoplasmic-like | F: nucleic acid binding, nucleotide binding, catalytic activity; P: nucleobase-containing compound metabolic process, translation; C: cytoplasm | |
| GRMZM2G019171_P01 | F-box kelch-repeat At1g74510 |  | |
| GRMZM2G019260_P01 | Phosphomevalonate kinase | F: nucleotide binding, kinase activity; P: lipid metabolic process, biosynthetic process | |
| GRMZM2G012200_P01 | RNA-binding Raly | C: membrane, plastid | |
| GRMZM2G012269_P01 | Membrane-anchored ubiquitin-fold 3 isoform X2 | F: binding; P: cellular homeostasis, transport; C: cytoplasm, plasma membrane | |
| GRMZM2G012319_P01 | SH3 domain containing expressed |  | |
| GRMZM2G012399_P01 | Alkylated DNA repair alkB homolog 8 | F: transferase activity; P: nucleobase-containing compound metabolic process; C: mitochondria | |
| GRMZM2G013115_P02 | Peroxisome biosynthesis PAS1-like isoform X1 | F: nucleotide binding, hydrolase activity; P: cellular component organization; C: nucleus | |
| GRMZM2G019597_P01 | tRNA (guanine-N(7)-)-methyltransferase | F: RNA binding, transferase activity; P: nucleobase-containing compound metabolic process; C: nucleus, cytoplasm | |
| OBF3.1 | bZIP transcription factor | F: nucleotide binding, DNA binding, kinase activity, transcription factor activity, sequence-specific DNA binding, protein binding; P: signal transduction, response to endogenous stimulus, cellular protein modification process, biosynthetic process, nucleobase-containing compound metabolic process; C: nucleus, cytoplasm | |
| GRMZM2G015126_P01 | Methyltransferase PMT7 | F: DNA binding, protein binding, transferase activity; P: biosynthetic process, nucleobase-containing compound metabolic process; C: endosome, membrane, Golgi apparatus, nucleolus, vacuole | |
| GRMZM2G015912_P01 | EF hand family | F: binding | |
| GRMZM2G016655_P01 | ER membrane complex subunit 1 | P: cellular process, transport; C: Golgi apparatus, endoplasmic reticulum, vacuole, plasma membrane | |
| pco077834b | Phosphatase 2C 39 | F: binding, hydrolase activity; P: cellular protein modification process; C: cell | |
| GRMZM2G010836_P02 | Vesicle transport v-SNARE 13 | F: protein binding; P: cellular component organization, transport; C: cytosol, endoplasmic reticulum, Golgi apparatus, vacuole, endosome, membrane | |
| GRMZM2G017086_P03 | Ubiquitin carboxyl-terminal hydrolase 6 | F: hydrolase activity; P: catabolic process, cellular protein modification process; C: cytoplasm | |
| pco136841 | ACD11 homolog | F: lipid binding, transporter activity; C: nucleus, cytoplasm | |
| GRMZM2G012046_P01 | MACPF domain-containing At4g24290-like |  | |
| GRMZM2G012178_P01 | Armadillo repeat-containing 6 |  | |
| GRMZM2G018566_P01 | Isocitrate dehydrogenase [NAD] catalytic subunit mitochondrial | F: nucleotide binding, catalytic activity; P: generation of precursor metabolites and energy; C: mitochondria | |
| GRMZM2G018573_P01 | Autophagy-related 18b | F: lipid binding; P: biosynthetic process, cellular component organization, cellular protein modification process; C: cytosol, membrane | |
| GRMZM2G018673_P01 | Hypothetical protein ZEAMMB73_937944 |  | |
| GRMZM2G019386_P01 | Holocarboxylase synthetase | C: mitochondria | |
| GRMZM2G019468_P01 | 26S proteasome non-ATPase regulatory subunit 11 homolog | C: mitochondria, cytosol | |
| pco126705 | Prolyl endopeptidase | F: hydrolase activity; P: protein metabolic process; C: cytoplasm | |
| GRMZM2G015955_P01 | Zinc transporter 7 | F: transporter activity; C: plasma membrane | |
| GRMZM2G011068_P01 | Cereblon-like isoform X1 | F: hydrolase activity; P: protein metabolic process | |
| GRMZM2G017426_P01 | WRKY transcription factor 19 | C: cytoplasm, membrane | |
| GRMZM2G017741_P01 | O-fucosyltransferase 1 isoform X1 | F: transferase activity; P: cellular protein modification process, biosynthetic process; C: membrane | |
| GRMZM2G019991_P02 | Golgin subfamily A  member 4 | C: cytosol | |
| GRMZM2G020098_P01 | Uncharacterized protein LOC100274029 | C: membrane | |
| IDP781 | Serine carboxypeptidase-like | F: hydrolase activity; P: protein metabolic process, catabolic process, cellular process; C: membrane, cytosol, extracellular region, vacuole | |
| GRMZM2G020761_P01 | Secologanin synthase | F: binding, catalytic activity; P: metabolic process; | |
| myb81 | Transcription factor DIVARICATA | F: DNA binding; P: biosynthetic process, nucleobase-containing compound metabolic process; C: nucleus | |
| bzip13 | Basic leucine zipper 43-like | F: DNA binding, transcription factor activity, sequence-specific DNA binding; P: biosynthetic process, nucleobase-containing compound metabolic process; C: intracellular | |
| GRMZM2G020996_P01 | E3 ubiquitin- ligase rbrA | F: nucleic acid binding, protein binding, transferase activity; P: catabolic process, cellular protein modification process; C: cytoplasm | |
| GRMZM2G020775_P01 | Sucrose cleavage | C: mitochondria, membrane | |
| GRMZM2G020982_P01 | Ankyrin repeat-containing At3g12360 | P: signal transduction; C: plasma membrane, plastid | |
| GRMZM2G021129_P01 | Transmembrane emp24 domain-containing p24delta9-like | P: transport; C: mitochondria, membrane, endoplasmic reticulum | |
| GRMZM2G021035_P01 | Hypothetical protein |  | |
| GRMZM2G021517_P01 | CBL-interacting kinase 8 | F: nucleotide binding, kinase activity; P: signal transduction, multicellular organism development, cellular protein modification process; C: plasma membrane, nucleus, cytoplasm | |
| ZCN25 | Flowering Locus T-like |  | |
| GRMZM2G021781_P01 | Cytochrome b5 | F: binding; C: mitochondria, membrane, plastid | |
| GRMZM2G021877_P01 | 60S ribosomal L18A | F: structural molecule activity, hydrolase activity; P: reproduction, translation, embryo development, post-embryonic development; C: mitochondria, membrane, cytosol, ribosome | |
| GRMZM2G022052_P01 | Transcription initiation factor TFIID subunit 11 | F: protein binding, translation factor activity, RNA binding; P: cellular component organization, nucleobase-containing compound metabolic process; C: nucleoplasm, ribosome | |
| GRMZM2G022090_P01 | Cysteine sulfinate desulfinase cysteine desulfurase and related enzymes | C: plastid | |
| CNX | Calnexin homolog | F: protein binding; P: cellular process; C: membrane, endoplasmic reticulum | |
| GRMZM2G022279_P02 | GDSL esterase lipase | F: hydrolase activity; P: catabolic process, lipid metabolic process, C: cytoplasm | |
| GRMZM2G022310_P03 | Phosphatidylinositol N-acetylglucosaminyl transferase subunit A | F: transferase activity; P: lipid metabolic process, cellular protein modification process, biosynthetic process | |
| GRMZM2G022403_P02 | TPA: hypothetical protein ZEAMMB73_764372 | C: membrane, endoplasmic reticulum | |
| GRMZM2G022629_P01 | Uncharacterized LOC103640569 |  | |
| GRMZM2G022642_P04 | Amino-acid permease BAT1 homolog isoform X2 | F: transporter activity; C: plasma membrane | |
| gpm464 | Katanin p60 ATPase-containing subunit A-like 2 | F: nucleotide binding, hydrolase activity; P: cellular component organization; C: nucleus | |
| GRMZM2G022836_P01 | Gibberellin 20 oxidase 2-like | F: binding, catalytic activity; P: metabolic process | |
| GRMZM2G023003_P01 | Callose synthase 8 isoform X1 | F: transferase activity; P: carbohydrate metabolic process, biosynthetic process, cellular process; C: plasma membrane | |
| GRMZM2G023068_P03 | 39S ribosomal L41- mitochondrial-like | F: structural molecule activity; P: translation; C: ribosome, plastid, mitochondria | |
| GRMZM2G022984_P01 | Uncharacterized protein LOC100381761 | C: membrane | |
| mterf11 | mTERF family | F: DNA binding; P: biosynthetic process, nucleobase-containing compound metabolic process; C: mitochondria | |
| GRMZM2G023520_P01 | Hypothetical protein ZEAMMB73_543053 | C: cytoplasm | |
| IDP343 | Vacuolar -sorting-associated  37 homolog 1-like isoform X2 | | |
| GRMZM2G023585_P02 | Axoneme-associated  mst101(2) |  | |
| GRMZM2G023791_P01 | Hypothetical protein | C: membrane | |
| rf1-C1-g7 | Shaggy-related kinase alpha | F: nucleotide binding, kinase activity; P: cellular protein modification process | |
| cc5 | Cysteine-ase inhibitor 3 | F: enzyme regulator activity; P: protein metabolic process, cellular process | |
| GRMZM2G024571_P01 | OBERON 4-like | P: embryonic meristem initiation, embryonic pattern specification, cell fate specification | |
| GRMZM2G025074_P01 | GRIP and coiled-coil domain-containing PFC0235w | F: DNA binding, transcription factor activity, sequence-specific DNA binding; P: biosynthetic process, nucleobase-containing compound metabolic process; C: nucleus, membrane | |
| GRMZM2G025109_P01 | Uncharacterized protein LOC100382321 | C: membrane, integral component of membrane | |
| pco110951 | bZIP transcription factor 60 | F: DNA binding, transcription factor activity, sequence-specific DNA binding; P: signal transduction, biosynthetic process, nucleobase-containing compound metabolic process, response to stress; C: nucleus, membrane, endoplasmic reticulum | |
| GRMZM2G025528_P05 | Ureide permease 1-like isoform X1 | P: transport; C: cytoplasm, membrane | |
| pco063924 | Transglutaminase | F: transferase activity; C: mitochondria | |
| GRMZM2G026654_P04 | TPA: hypothetical protein ZEAMMB73_301536 | C: plasma membrane | |
| GRMZM2G027173_P01 | ELMO domain-containing 2 |  | |
| umc2386 | Uncharacterized endoplasmic reticulum membrane YGL010W | C: membrane | |
| GRMZM2G027209_P02 | General transcription factor IIH subunit 3 | F: DNA binding, hydrolase activity, translation factor activity, RNA binding, kinase activity; P: cellular protein modification process, DNA metabolic process, response to stress; C: ribosome, nucleoplasm, membrane | |
| TIDP3734 | HVA22 g | C: cytoplasm, membrane | |
| GRMZM2G027019_P01 | Vesicle-associated membrane 711 | F: protein binding; P: cellular component organization, transport; C: cytoplasm, membrane | |
| AY105910 | 26S protease regulatory subunit 6A homolog | F: nucleotide binding, protein binding, hydrolase activity; P: protein metabolic process, catabolic process, cellular component organization, biosynthetic process, nucleobase-containing compound metabolic process, response to stress; C: nucleus, cytosol | |
| GRMZM2G026447_P01 | MACPF domain-containing At4g24290-like |  | |
| GRMZM2G027462_P01 | DsRNA-binding 6 | F: RNA binding; P: cellular component organization, nucleobase-containing compound metabolic process, regulation of gene expression_epigenetic, translation; C: cytoplasm | |
| GRMZM2G028086_P01 | Calcium-dependent kinase 8-like | F: nucleotide binding, protein binding, kinase activity; P: signal transduction, response to endogenous stimulus, cellular protein modification process; C: plasma membrane, nucleus, cytoplasm | |
| GRMZM2G028346_P01 | Proteasome subunit alpha type 3 | F: hydrolase activity; P: cellular process, protein metabolic process, catabolic process; C: membrane, cytosol, extracellular region, vacuole, nucleus | |
| cl9685_1b | MACPF domain-containing At4g24290-like | C: plasma membrane | |
| cop2 | Coatomer subunit epsilon-1 | F: structural molecule activity; P: transport; C: cytoplasm, membrane, Golgi apparatus | |
| pco149758 | Beta-adaptin A | F: transporter activity; C: cytoplasm, membrane | |
| Pti1c | PTI1-like tyrosine- kinase 3 | F: nucleotide binding, kinase activity; cellular protein modification process | |
| umc2170 | Electron transporter, heat shock binding | F: binding; C: plastid | |
| GRMZM2G029583_P02 | 26S proteasome non-ATPase regulatory subunit 6 | P: protein metabolic process, catabolic process, cellular process; C: intracellular | |
| PCNA | Proliferating cell nuclear antigen | F: DNA binding, chromatin binding, enzyme regulator activity; P: response to abiotic stimulus, biosynthetic process, DNA metabolic process, response to stress; C: nucleus | |
| nac99 | NAC domain transcription factor superfamily | F: DNA binding; P: biosynthetic process, nucleobase-containing compound metabolic process; C: nucleus | |
| tif-4A3 | Eukaryotic initiation factor 4A | F: nucleotide binding, hydrolase activity, translation factor activity, RNA binding; P: cellular component organization, anatomical structure morphogenesis, nucleobase-containing compound metabolic process, cell growth; C: ribosome | |
| RPL30 | 60S ribosomal L30 | F: RNA binding, structural molecule activity; P: translation; C: cytosol, ribosome | |
| TIDP9256 | Uncharacterized protein LOC100275193 | C: membrane | |
| GRMZM2G030139_P01 | TPA: pyruvate dehydrogenase kinase family | F: nucleotide binding, receptor activity, kinase activity, signal transducer activity; C: cellular component | |
| bzip72 | Transcription factor HBP-1b(c38) isoform X1 | F: DNA binding, transcription factor activity, sequence-specific DNA binding; P: biosynthetic process, nucleobase-containing compound metabolic process; C: nucleus | |
| GRMZM2G027307_P01 | S-acyltransferase 14 | F: binding, transferase activity; P: cellular process, biosynthetic process; C: membrane | |
| GRMZM2G027741_P02 | TOM1 2 | P: transport; C: intracellular | |
| GRMZM2G028369_P01 | Chorismate mutase | F: catalytic activity; P: biosynthetic process, cellular process | |
| IDP294 | Calcium-dependent kinase 13 | F: nucleotide binding, protein binding, kinase activity; P: signal transduction, response to endogenous stimulus, cellular protein modification process; C: plasma membrane, nucleus, cytoplasm | |
| pco079257 | Unknown | P: cellular component organization | |
| cl40463_1a | Small G signaling modulator 1-like isoform X1 | F: nucleotide binding, RNA binding, protein binding, transferase activity, enzyme regulator activity; P: cellular component organization, biosynthetic process, transport; C: nucleus, cytoplasm | |
| cl12600_1 | Mediator of RNA polymerase II transcription subunit 27 isoform X1 | P: metabolic process, cellular process; C: nucleoplasm, cytoplasm | |
| GRMZM2G031461_P01 | Nudix hydrolase, chloroplastic | F: hydrolase activity; C: plastid | |
| GRMZM2G031545_P02 | Elongation factor 1-delta 1 | F: translation factor activity, RNA binding; C: ribosome | |
| GRMZM2G031572_P01 | Serine protease EDA2 | F: hydrolase activity; P: protein metabolic process; C: cytoplasm | |
| pco119358 | Calcyclin-binding -like |  | |
| GRMZM2G031824_P01 | TPA: hypothetical protein ZEAMMB73_635026 | C: mitochondria | |
| wrky59 | WRKY transcription  factor 34 | F: DNA binding, transcription factor activity, sequence-specific DNA binding; P: biosynthetic process, nucleobase-containing compound metabolic process; C: intracellular | |
| GRMZM2G032028_P01 | Uncharacterized protein LOC100191857 isoform 2 | C: membrane | |
| AY110632 | DUF538 family protein |  | |
| GRMZM2G032154_P01 | Uncharacterized protein LOC103647767 | F: nucleotide binding, kinase activity; P: nucleobase-containing compound metabolic process, reproduction; C: plastid, cytosol | |
| GRMZM2G032218_P02 | ABC transporter C family member 15 | F: nucleotide binding, transporter activity, hydrolase activity; C: membrane | |
| GRMZM2G032852_P02 | Calcium-dependent kinase isoform 11 | F: nucleotide binding, protein binding, kinase activity; P: signal transduction, response to endogenous stimulus, cellular protein modification process; C: plasma membrane, nucleus, cytoplasm | |
| GRMZM2G032955_P01 | PRA1 family F3 | C: membrane, plastid | |
| GRMZM2G033626_P01 | 26S proteasome non-ATPase regulatory subunit 14 homolog | P: generation of precursor metabolites and energy, catabolic process, post-embryonic development, cellular protein modification process, biosynthetic process, nucleobase-containing compound metabolic process, transport, response to stress, carbohydrate metabolic process, response to abiotic stimulus, lipid metabolic process, cellular component organization; C: cytosol, nucleus | |
| pco075539 | Aspartate aminotransferase | F: transferase activity, binding; P: embryo development, post-embryonic development, biosynthetic process, reproduction, cellular process; C: plastid | |
| GRMZM2G033846_P01 | Caltractin | F: binding; C: cytosol | |
| pco121228 | LOC100281547 isoform X1 | F: kinase activity; P: phosphorylation | |
| GRMZM2G033930_P01 | Glucuronokinase 1 | F: nucleotide binding, kinase activity; C: cytoplasm | |
| pco080711 | ATP-citrate synthase alpha chain 3 | F: nucleotide binding, transferase activity; P: generation of precursor metabolites and energy, lipid metabolic process; C: cytosol | |
| TIDP3321 | Transcription factor MYB1R1 | F: DNA binding; P: response to endogenous stimulus, biosynthetic process, nucleobase-containing compound metabolic process; C: plastid, C: nucleus | |
| hb126 | Homeobox-leucine zipper HOX6 | F: DNA binding, transcription factor activity, sequence-specific DNA binding; P: biosynthetic process, nucleobase-containing compound metabolic process; C: nucleus | |
| GRMZM2G034122_P01 | ER membrane complex subunit 2-A-like |  | |
| GRMZM2G034225_P03 | ABC transporter A family member 1 | C: membrane | |
| pco109481 | Uncharacterized Rho GTPase-activating At5g61530 | P: signal transduction; C: cytoplasm | |
| GRMZM2G034748_P01 | F-box LRR-repeat At3g28410 | C: mitochondria | |
| GRMZM2G034882_P01 | WPP domain-interacting 1-like | C: membrane | |
| mads53 | MADS transcription partial | F: DNA binding, protein binding; P: biosynthetic process, nucleobase-containing compound metabolic process; C: nucleus | |
| GRMZM2G035118_P01 | Pumilio homolog 5 | F: RNA binding | |
| GRMZM2G035131_P01 | Monoglyceride lipase | F: hydrolase activity; P: catabolic process, lipid metabolic process | |
| GRMZM2G035217_P01 | UPF0496 1 | C: membrane, plastid, membrane | |
| GRMZM2G035243_P01 | F-box kelch-repeat At1g74510 |  | |
| GRMZM2G035298_P02 | Peroxisomal adenine nucleotide carrier 1-like | F: structural molecule activity; P: transport, translation; C: membrane, ribosome | |
| GRMZM2G035341_P02 | RING-box 1a | F: protein binding, transferase activity; P: catabolic process, cellular protein modification process; C: nucleus | |
| GRMZM2G035421_P01 | Triacylglycerol lipase SDP1-like | F: hydrolase activity; P: catabolic process, lipid metabolic process, cellular process; C: membrane | |
| gpm882 | Signal peptidase complex catalytic subunit SEC11A-like | F: hydrolase activity; P: protein metabolic process, cellular process; C: membrane | |
| GRMZM2G035595_P01 | Hydroxyproline O-arabinosyltransferase 3 | C: cytoplasm, membrane | |
| GRMZM2G035636_P01 | Aspartic protease oryzasin-1 precursor | F: binding, hydrolase activity; P: protein metabolic process, catabolic process, lipid metabolic process; C: vacuole | |
| GRMZM2G035719_P03 | TPA: RNA recognition motif containing family | F: nucleotide binding, nucleic acid binding | |
| GRMZM2G035726_P01 | Cysteine protease 1 precursor | F: hydrolase activity; P: protein metabolic process, transport; C: membrane | |
| SnRK2.1 | Serine threonine- kinase SAPK1 | F: nucleotide binding, kinase activity; P: signal transduction, response to endogenous stimulus, cellular protein modification process; C: nucleus, cytoplasm | |
| GRMZM2G036245_P01 | TPA: hypothetical protein  ZEAMMB73_096097 | | |
| GRMZM2G036502_P01 | Nuclear RNA export  factor SDE5 | P: nucleobase-containing compound metabolic process, regulation of gene expression,_epigenetic; C: membrane | |
| GRMZM2G036765_P01 | Cell division cycle 48  homolog | F: nucleotide binding, hydrolase activity; P: cellular process | |
| GRMZM2G036940_P01 | ABC transporter G family  member 23 | F: nucleotide binding, transporter activity, hydrolase activity; C: plasma membrane | |
| pco132830 | Coatomer subunit gamma-2 | F: structural molecule activity; P: transport; C: cytoplasm, membrane, Golgi apparatus | |
| GRMZM2G035849_P02 | S-acyltransferase 16 | F: binding, transferase activity; P: biosynthetic process, cellular process; C: cytoplasm, membrane | |
| GRMZM2G037204_P01 | GTP-binding YPTM2 | F: nucleotide binding, DNA binding, hydrolase activity; P: signal transduction, biosynthetic process, nucleobase-containing compound metabolic process, transport; C: plasma membrane, cytoplasm | |
| GRMZM2G037209_P03 | Uncharacterized protein LOC100193175 | C: membrane | |
| GRMZM2G038126_P01 | 26S protease regulatory  subunit 6B homolog | F: nucleotide binding, protein binding, hydrolase activity; P: protein metabolic process, catabolic process, biosynthetic process, nucleobase-containing compound metabolic process, response to stress, cellular component organization; C: cell wall, membrane, cytosol, nucleus | |
| GRMZM2G038217_P01 | Uncharacterized protein LOC101202720 precursor | C: vacuole | |
| sar1 | GTP-binding SAR1A | F: nucleotide binding, transporter activity, hydrolase activity; C: endoplasmic reticulum, Golgi apparatus | |
| GRMZM2G038532_P01 | Uncharacterized protein LOC103637883 | F: transferase activity; P: carbohydrate metabolic process, biosynthetic process, nucleobase-containing compound metabolic process; C: membrane | |
| GRMZM2G038880_P01 | C2 and GRAM domain plant | C: membrane, integral component of membrane | |
| GRMZM2G038931_P02 | Uncharacterized protein LOC100276373 | C: membrane | |
| GRMZM2G038953_P01 | Signal recognition particle 54 kDa 2 | F: nucleotide binding, RNA binding, hydrolase activity; P: cellular component organization, transport; C: cytoplasm | |
| GRMZM2G039373_P01 | Trafficking particle complex subunit 1 | F: molecular function; P: transport; C: cytosol | |
| cl22071_1 | Phosphatidylserine  synthase 2 | F: transferase activity; P: lipid metabolic process, biosynthetic process, cell cycle; C: nucleus, membrane, endoplasmic reticulum | |
| TIDP3380 | RTE1-HOMOLOG | C: cytoplasm, membrane | |
| GRMZM2G039622_P01 | bet1 At4g14600 | C: membrane | |
| GRMZM2G039811_P01 | Transmembrane 9 superfamily member 8 | C: cytoplasm, membrane | |
| bzip6 | bZIP transcription factor superfamily | F: DNA binding, transcription factor activity, sequence-specific DNA binding; P: biosynthetic process, nucleobase-containing compound metabolic process; C: plastid, mitochondria | |
| GRMZM2G039978_P01 | CASP 5C1 | C: mitochondria, plasma membrane | |
| LIP1P | Lipoyl synthase, chloroplastic | F: transferase activity, binding; P: lipid metabolic process, cellular protein modification process, biosynthetic process; C: plastid | |
| gpm455 | Cold acclimation COR413-PM1 | C: membrane | |
| GRMZM2G040033_P01 | UDP-galactose transporter 1 | C: membrane | |
| GRMZM2G040164_P01 | Proteasome subunit beta type-4 | F: hydrolase activity; P: protein metabolic process, catabolic process, cellular process; C: cytosol, ribosome, nucleus | |
| GRMZM2G040424_P01 | Peroxisomal membrane PMP22 | C: membrane | |
| GRMZM2G040493_P01 | TPA: hypothetical protein ZEAMMB73_053573, partial | C: plasma membrane | |
| GRMZM2G040515_P02 | Pyrrolidone-carboxylate peptidase | F: hydrolase activity; P: protein metabolic process; C: cytosol | |
| GRMZM2G040548_P01 | Exocyst complex component SEC6 | P: transport, cellular process; C: cytoplasm | |
| GRMZM2G040587_P01 | Mitochondrial import  inner membrane translocase  subunit TIM14 | | |
| gpm77 | DNA-directed RNA  polymerase V subunit 7-like | F: carbohydrate binding, kinase activity, nucleotide binding, DNA binding, RNA binding, protein binding; P: catabolic process, cellular protein modification process, nucleobase-containing compound metabolic process, translation; C: mitochondria, membrane, nucleolus, nucleoplasm | |
| phm3342 | ABIL1 | C: cytoskeleton, cytoplasm | |
| hb128 | Homeobox-leucine zipper HOX6-like | F: DNA binding, transcription factor activity, sequence-specific DNA binding; P: biosynthetic process, nucleobase-containing compound metabolic process; C: nucleus | |
| GRMZM2G041463_P01 | CBL-interacting kinase 18 | F: nucleotide binding, kinase activity; P: signal transduction, cellular protein modification process; C: membrane | |
| GRMZM2G041472_P01 | WD repeat-containing 43 isoform X1 | C: cellular component | |
| GRMZM2G041699_P02 | Cytokinin-O-glucosyltransferase 2 | F: transferase activity; P: biosynthetic process, cellular process C: intracellular | |
| CBL5 | Calcineurin B 5 | F: binding | |
| GRMZM2G041770_P01 | Myosin-binding 2-like | C: membrane | |
| GRMZM2G126920_P01 | FBD-associated F-box At1g61330 isoform X1 | F: protein binding | |
| GRMZM2G328374_P01 | Splicing factor U2af large subunit B | F: oxidoreductase activity; P: oxidation-reduction process | |
| AC191070.3_FGP006 | Vesicle-associated 1-2 | C: endoplasmic reticulum membrane, integral component of membrane | |
| AC212187.4_FGP001 | RING-H2 finger ATL39-like | F: zinc ion binding, ubiquitin protein ligase activity; P: proteasome-mediated ubiquitin-dependent protein catabolic process; C: integral component of membrane | |
| GRMZM2G009232_P01 | Isoflavone 2 -hydroxylase-like | F: iron binding, heme binding; oxidoreductase activity, acting on paired donors, with incorporation or reduction of molecular oxygen, NAD(P)H as one donor, and incorporation of one atom of oxygen; P: defense response to other organism, secondary metabolite biosynthetic process, indole glucosinolate metabolic process, oxidation-reduction process; C: integral component of membrane | |
| GRMZM2G011151_P01 | Alpha-humulene synthase-like | F: metal ion binding, terpene synthase activity P: metabolic process | |
| GRMZM2G022979_P01 | DNA-directed RNA polymerase II subunit, RPB1-like | C: integral component of membrane | |
| GRMZM2G034471_P01 | Cytochrome P450 78A9-like | F: iron ion binding, heme binding, oxidoreductase activity, acting on paired donors, with incorporation or reduction of molecular oxygen, NAD(P)H as one donor, and incorporation of one atom of oxygen; P: secondary metabolite biosynthetic process, oxidation-reduction process; C: integral component of membrane | |
| GRMZM2G038301_P01 | Cytochrome C biogenesis, chloroplastic | P: carotenoid biosynthetic process, phosphatidylglycerol biosynthetic process, leaf morphogenesis, maltose metabolic process, starch biosynthetic process, positive regulation of catalytic activity, cell differentiation, thylakoid membrane organization, isopentenyl diphosphate biosynthetic process_ methylerythritol 4-phosphate pathway, cytochrome complex assembly, mRNA modification; C: chloroplast thylakoid membrane, integral component of membrane | |
| GRMZM2G044469_P01 | Indole-3-acetaldehyde oxidase-like | F: xanthine dehydrogenase activity, oxidoreductase activity, acting on CH-OH group of donors; iron ion binding, 2 iron, 2 sulfur cluster binding; oxidoreductase activity, acting on the aldehyde or oxo group of donors; flavin adenine dinucleotide binding, electron carrier activity; P: xanthine catabolic process, oxidation-reduction process; C: plastid, cytosol | |
| GRMZM2G044884_P01 | DIMBOA UDP-glucosyltransferase BX8-like | F: UDP-glucosyltransferase activity; P: flavonoid metabolic process | |
| GRMZM2G047763_P01 | DNA-directed RNA polymerase II subunit RPB1-like | C: integral component of membrane | |
| GRMZM2G047910_P01 | DIMBOA UDP-glucosyltransferase BX9-like | F: quercetin 3-O-glucosyltransferase activity, quercetin 7-O-glucosyltransferase activity; P: flavonoid glucuronidation, flavonoid biosynthetic process; C: intracellular membrane-bounded organelle | |
| GRMZM2G062613_P01 | HIPL1 | F: transporter activity, oxidoreductase activity, acting on the CH-OH group of donors, quinone or similar compound as acceptor; quinone binding; P: carbohydrate metabolic process, oxidation-reduction process, transport; C: mitochondria, integral component of membrane | |
| GRMZM2G070503_P01 | Disease resistance RPP13 1 | F: ADP binding | |
| GRMZM2G071448_P01 | Signal transducer | F: protein binding; C: nucleus | |
| GRMZM2G074754_P01 | FT-interacting 1-like | F: protein binding, calcium ion binding, calcium-dependent phospholipid binding; C: integral component of membrane, plasma membrane | |
| GRMZM2G091742_P01 | Calcium uniporter mitochondrial-like | F: uniporter activity P: transmembrane transport; C: mitochondria | |
| GRMZM2G097109_P01 | Myelin-associated oligodendrocyte basic isoform 1 | C: integral component of membrane, proteasome complex, plastid | |
| GRMZM2G099382_P01 | Tonoplast dicarboxylate transporter | F: calcium ion binding, malate transmembrane transporter activity; P: sodium ion transport, regulation of intracellular pH, malate transmembrane transport; C: vacuole, integral component of membrane | |
| GRMZM2G113415_P04 | Leukotriene A-4 hydrolase | F: adenine phosphoribosyltransferase activity; P: nucleoside metabolic process, adenine salvage; C: cytoplasm | |
| GRMZM2G122116_P01 | Pentatricopeptide repeat-containing chloroplastic | F: calmodulin binding; P: tRNA metabolic process, transcription from plastid promoter; response to stress, leaf morphogenesis, chloroplast relocation, photosystem II assembly, positive regulation of transcription, DNA-templated; cell differentiation, gamma-tubulin complex localization, thylakoid membrane organization, regulation of protein dephosphorylation; C: gamma-tubulin ring complex, plastid chromosome | |
| GRMZM2G123940_P02 | Serine carboxypeptidase-like 45 | F: transferase activity, transferring acyl groups other than amino-acyl groups, serine-type carboxypeptidase activity; P: secondary metabolic process, proteolysis involved in cellular protein catabolic process | |
| GRMZM2G131525_P01 | Syntaxin-related KNOLLE | F: SNAP receptor activity, SNARE binding, heme binding, proton-transporting ATP synthase activity_ rotational mechanism, peroxidase activity, intracellular protein transport, exocytosis, response to oxidative stress, vesicle fusion, vesicle docking, cellular oxidant detoxification, oxidation-reduction process, ATP synthesis coupled proton transport C: integral component of membrane, endomembrane system, SNARE complex, plasma membrane, phragmoplast, cell plate, plasmodesma | |
| GRMZM2G132238_P02 | Metacaspase family | F: peptidase activity; P: proteolysis | |
| GRMZM2G132682_P01 | BAG family molecular chaperone regulator 1-like | F: protein binding; C: mitochondria | |
| GRMZM2G133966_P01 | Trehalose-6-phosphate synthase12 | F: alpha, alpha-trehalose-phosphate synthase (UDP-forming) activity; P: trehalose biosynthetic process | |
| GRMZM2G135877_P01 | DETOXIFICATION 48-like | F: antiporter activity, drug transmembrane transporter activity; P: drug transmembrane transport; C: integral component of membrane, Golgi transport complex | |
| GRMZM2G138585_P01 | Membrane lipo | C: membrane | |
| GRMZM2G140078_P01 | Trehalose-phosphate phosphatase 7 | F: trehalose-phosphatase activity; P: trehalose biosynthetic process, dephosphorylation | |
| GRMZM2G151700_P02 | Actin associated | C: plastid | |
| GRMZM2G153575_P01 | Trihelix transcription factor ASIL1-like | F: metal ion binding, GTPase activity | |
| GRMZM2G154056_P01 | Unknown | C: integral component of membrane | |
| GRMZM2G156904_P01 | S-adenosyl-L-methionine-dependent methyltransferase superfamily | F: S-adenosylmethionine-dependent methyltransferase activity; P: methylation; C: trans-Golgi network, endosome, integral component of membrane, vacuolar membrane | |
| GRMZM2G173710_P02 | Histidine-containing phosphotransfer 4 | F: histidine phosphotransfer kinase activity, protein histidine kinase binding; P: phosphorelay signal transduction system, phosphorylation, cytokinin-activated signaling pathway; C: nucleus, cytoplasm | |
| GRMZM2G176340_P01 | Tubby-like F-box 5 | F: protein binding, phosphatidylinositol binding; P: protein localization to cilium; C: cilium | |
| GRMZM2G179490_P01 | Tubulin alpha chain-like | F: GTP binding, GTPase activity, transferase activity; P: microtubule-based process; C: microtubule | |
| GRMZM2G180054_P01 | Calcium-binding CML12 | F: calcium ion binding; C: mitochondria | |
| GRMZM2G316904_P01 | LRR receptor-like serine threonine- kinase At3g47570 | F: ATP binding, protein serine/threonine kinase activity; P: protein phosphorylation; C: integral component of membrane | |
| GRMZM2G319062_P01 | Polyphenol oxidase, chloroplastic-like | F: protein binding, metal ion binding, catechol oxidase activity; P: oxidation-reduction process | |
| GRMZM2G325247_P01 | Armadillo repeat-containing kinesin 3 | F: ATP binding, microtubule binding, ATP-dependent microtubule motor activity, plus-end-directed; P: root development, cytoskeleton-dependent intracellular transport, protein localization, microtubule-based movement, regulation of microtubule-based process; C: cytoplasm, kinesin complex, microtubule | |
| GRMZM2G337532_P01 | LRR receptor-like serine threonine- kinase At1g56130 | F: ATP binding, protein binding, protein serine/threonine kinase activity; P: protein phosphorylation; C: mitochondria, integral component of membrane, plasma membrane | |
| GRMZM2G344416_P01 | N-terminal domain containing isoform X1 | P: chiasma assembly, double-strand break repair via homologous recombination; C: nucleus | |
| GRMZM2G350023_P01 | Auxin-responsive SAUR71 | F: calmodulin binding; P: auxin-activated signaling pathway; C: mitochondria, plastid | |
| GRMZM2G377686_P01 | Hypothetical protein ZEAMMB73_Zm00001d050529 | C: SAGA-type complex | |
| GRMZM2G412440_P01 | Hypothetical protein | C: integral component of membrane | |
| GRMZM2G439596_P01 | Plastid division PDV1 | C: integral component of membrane | |
| GRMZM2G472821_P01 | Eukaryotic peptide chain release factor subunit 1-3 | F: translation release factor activity- codon specific; P: translational termination; C: cytoplasm | |
| GRMZM2G527017_P01 | Leucine-rich repeat extensin 5 | C: integral component of membrane | |
| GRMZM2G700046_P01 | Unknown | C: integral component of membrane | |
| GRMZM2G700188_P05 | Cinnamyl alcohol dehydrogenase 8C | F: zinc ion binding, cinnamyl-alcohol dehydrogenase activity, sinapyl alcohol dehydrogenase activity; P: lignin biosynthetic process, oxidation-reduction process; C: plastid | |
| GRMZM2G702522_P01 | DNA-directed RNA polymerase II subunit RPB1-like | C: integral component of membrane | |
| GRMZM5G834532_P01 | Uncharacterized protein LOC100502240 | C: integral component of membrane | |
| GRMZM5G868959_P01 | Unknown | C: integral component of membrane | |
| MAX1b | Cytochrome P450 711A1 | F: monooxygenase activity, heme binding, iron ion binding, oxidoreductase activity- acting on paired donors with incorporation or reduction of molecular oxygen; P: oxidation-reduction process; C: mitochondria, integral component of membrane | |
| ZIP4 | Zinc transporter ZIP1 | F: zinc ion binding, zinc ion transmembrane transporter activity; P: response to zinc ion, zinc II ion transmembrane transport; C: plasma membrane, integral component of membrane | |
| bhlh96 | Transcription factor ICE1 | F: protein dimerization activity; C: nucleus | |
| jmj11 | Lysine-specific demethylase JMJ705-like | F: nucleic acid binding, metal ion binding, histone demethylase activity (H3-K27 specific); positive regulation of gene expression- epigenetic; P: positive regulation of growth rate; C: nucleus | |
| ofp38 | Transcription repressor OFP13 | F: nucleic acid binding, protein dimerization activity | |
| pco078735 | TPR-containing kinase | F: ATP binding, protein binding, protein kinase activity; P: protein phosphorylation | |
| pco137413a | Choline transporter 2 | C: integral component of membrane | |
| phyB2 | Phytochrome B | F: phosphorelay sensor kinase activity, protein homodimerization activity, photoreceptor activity; P: red, far-red light phototransduction, detection of visible light, phosphorelay signal transduction system, regulation of transcription- DNA-templated, protein-chromophore linkage, signal transduction by protein phosphorylation, protein-tetrapyrrole linkage; C: nucleus | |
| umc1236 | Bidirectional sugar transporter SWEET17-like | F: sugar transmembrane transporter activity; P: carbohydrate transmembrane transport; C: integral component of membrane; | |
| umc1283 | Ran BP2/NZF zinc finger-  Like superfamily protein | F: zinc ion binding | |
| AC217841.3_FGP001 | Hypothetical protein ZEAMMB73_Zm00001d040906 | C: membrane, integral component of membrane | |
| GRMZM2G124290_P01 | Flocculation FLO11-like | C: plastid | |
| GRMZM2G132966_P01 | Hypothetical protein | C: membrane, integral component of membrane | |
| GRMZM2G533031_P01 | NAD(P)-linked oxidoreductase superfamily | F: oxidoreductase activity; P: oxidation-reduction process; C: membrane, integral component of membrane | |
| AC187065.3_FGP003 | Hypothetical protein ZEAM  MB73_Zm00001d009051 | | |
| AC190763.3_FGP002 | Hypothetical protein ZEAM  MB73_Zm00001d030602 | | |
| AC195914.2_FGP003 | Uncharacterized protein LOC100280277 | |  |
| AC210035.3_FGP002 | Uncharacterized protein LOC100275833 | |  |
| AC210158.3_FGP004 | Hypothetical protein ZEAM  MB73_Zm00001d045016 | | |
| GRMZM2G000448_P01 | Uncharacterized protein  LOC100384438 precursor | | |
| GRMZM2G001973_P01 | Remorin family | |  |
| GRMZM2G004054_P01 | Uncharacterized protein LOC100383353 | |  |
| GRMZM2G008474_P01 | Hypothetical protein ZEAM  MB73_Zm00001d009558 | | |
| GRMZM2G009065_P01 | Uncharacterized protein LOC100272308 | |  |
| GRMZM2G020411_P01 | Uncharacterized protein LOC100216994 | |  |
| GRMZM2G025159_P01 | Unknown | |  |
| GRMZM2G025477_P01 | Uncharacterized protein LOC100273599 | |  |
| GRMZM2G027490_P01 | Uncharacterized protein  LOC103638202 | | |
| GRMZM2G032193_P01 | Uncharacterized protein LOC100381790 | |  |
| GRMZM2G037751_P01 | Uncharacterized protein LOC100275412 | |  |
| GRMZM2G038116_P01 | Uncharacterized protein LOC100217121 | |  |
| GRMZM2G041442_P01 | Uncharacterized protein LOC100383786 | |  |
| GRMZM2G046430_P02 | Uncharacterized protein LOC100273943 | |  |
| GRMZM2G051192_P01 | Uncharacterized protein LOC100193931 | |  |
| GRMZM2G056022_P01 | Hypothetical protein ZEAM  MB73_Zm00001d009558 | | |
| GRMZM2G064008_P01 | Unknown | |  |
| GRMZM2G064250_P01 | Uncharacterized protein  LOC103650387 | | |
| GRMZM2G071664_P01 | Hypothetical protein ZEAM  MB73_Zm00001d042789 | | |
| GRMZM2G071666_P01 | Hypothetical protein ZEAM  MB73_Zm00001d042789 | | |
| GRMZM2G072133_P01 | Uncharacterized protein LOC100217029 | |  |
| GRMZM2G072417_P01 | Uncharacterized protein LOC100303911 | |  |
| GRMZM2G075018_P01 | Uncharacterized protein LOC100383107 | |  |
| GRMZM2G077162_P01 | Uncharacterized protein LOC100274876 | |  |
| GRMZM2G078407_P01 | Uncharacterized protein LOC100384502 | |  |
| GRMZM2G079790_P01 | Uncharacterized protein LOC100384184 | |  |
| GRMZM2G081377_P01 | Uncharacterized protein  LOC100275833 isoform X1 | | |
| GRMZM2G082608_P01 | Uncharacterized protein  LOC100275900 | | |
| GRMZM2G082714_P01 | Uncharacterized protein LOC100273883 | |  |
| GRMZM2G087575_P01 | Uncharacterized protein LOC100274070 | |  |
| GRMZM2G087585_P01 | Uncharacterized protein LOC100274070 | |  |
| GRMZM2G089750_P01 | Uncharacterized protein LOC100193060 | |  |
| GRMZM2G090566_P01 | Uncharacterized protein LOC100192803 | |  |
| GRMZM2G093320_P01 | Uncharacterized protein  LOC103639283 | | |
| GRMZM2G102860_P01 | Uncharacterized protein  LOC103632424 | | |
| GRMZM2G103595_P01 | ROTUNDIFOLIA like 8 | |  |
| GRMZM2G104581_P01 | Unknown | |  |
| GRMZM2G108663_P01 | Uncharacterized protein LOC100279648 | |  |
| GRMZM2G109229_P01 | Uncharacterized protein LOC100383116 | |  |
| GRMZM2G111189_P01 | Uncharacterized protein LOC100383286 | |  |
| GRMZM2G113883_P01 | Uncharacterized protein  LOC103638507 | | |
| GRMZM2G117436_P01 | Hypothetical protein | |  |
| GRMZM2G123842_P01 | Uncharacterized protein LOC100383643 | |  |
| GRMZM2G130640_P01 | Uncharacterized protein  LOC103632424 | | |
| GRMZM2G131319_P01 | Uncharacterized protein LOC100383731 | |  |
| GRMZM2G133862_P01 | Uncharacterized protein  LOC103626974 | | |
| GRMZM2G133870_P01 | Uncharacterized protein  LOC103626974 | | |
| GRMZM2G135228_P01 | Uncharacterized protein LOC100192582 | |  |
| GRMZM2G137215_P01 | Uncharacterized protein  LOC103638507 | | |
| GRMZM2G147301_P01 | Uncharacterized protein LOC100384367 | |  |
| GRMZM2G148675_P01 | Uncharacterized protein  LOC103626846 | | |
| GRMZM2G151365_P01 | Uncharacterized protein LOC100384324 | |  |
| GRMZM2G162336_P01 | Hypothetical protein | |  |
| GRMZM2G163542_P01 | Hypothetical protein ZEAM  MB73_Zm00001d010845 | | |
| GRMZM2G164704_P01 | Uncharacterized protein LOC100277995 | |  |
| GRMZM2G165308_P01 | Uncharacterized protein LOC100272626 | |  |
| GRMZM2G165867_P01 | Uncharacterized protein LOC100273776 | |  |
| GRMZM2G166804_P01 | Serine arginine repetitive  matrix 3-like | |  |
| GRMZM2G177863_P01 | NAD(P)-binding Rossmann  -fold superfamily | | |
| GRMZM2G300916_P01 | Uncharacterized protein LOC100383112 | |  |
| GRMZM2G307553_P01 | Uncharacterized protein LOC100384753 | |  |
| GRMZM2G323413_P01 | Hypothetical protein ZEAM  MB73_Zm00001d010005 | | |
| GRMZM2G328005_P01 | Hypothetical protein ZEAM  MB73_Zm00001d040910 | | |
| GRMZM2G333013_P01 | Uncharacterized protein  LOC100275412 | |  |
| GRMZM2G356256_P01 | Uncharacterized protein  LOC100194353 precursor | | |
| GRMZM2G356338_P01 | Unknown | |  |
| GRMZM2G361402_P01 | Spidroin-1-like | |  |
| GRMZM2G370924_P01 | Uncharacterized protein LOC100192508 | |  |
| GRMZM2G380112_P01 | Uncharacterized protein LOC100194171 | |  |
| GRMZM2G386294_P01 | Uncharacterized protein LOC100192962 | |  |
| GRMZM2G393180_P01 | Uncharacterized protein  LOC103639283 | | |
| GRMZM2G413422_P01 | Uncharacterized protein LOC100280327 | |  |
| GRMZM2G414563_P01 | Uncharacterized protein LOC100501198 | |  |
| GRMZM2G444743_P01 | Uncharacterized protein LOC100383489 | |  |
| GRMZM2G461566_P01 | Uncharacterized protein  LOC103650387 | | |
| GRMZM2G464676_P01 | Uncharacterized protein LOC100279989 | |  |
| GRMZM2G471362_P01 | Hypothetical protein ZEAM  MB73_Zm00001d024151 | | |
| GRMZM2G471525_P01 | Octapeptide-repeat T2-like | |  |
| GRMZM2G474019_P01 | Uncharacterized protein LOC100193017 | |  |
| GRMZM2G524711_P02 | Inter-alpha-trypsin inhibitor,  heavy chain-related | | |
| GRMZM2G557357_P01 | Uncharacterized protein LOC100384148 | |  |
| GRMZM2G701047_P01 | Uncharacterized protein  LOC103629834 | | |
| GRMZM2G702129_P01 | Uncharacterized protein  LOC103646825 | | |
| GRMZM5G804251_P01 | Uncharacterized protein  LOC100384438 precursor | | |
| GRMZM5G811373_P02 | MOB kinase activator-like 1 | |  |
| GRMZM5G824236_P01 | Hypothetical protein | |  |
| GRMZM5G828284_P01 | Uncharacterized protein  LOC103634113 | | |
| GRMZM5G828422_P01 | Tubby-like F-box partial | |  |
| GRMZM5G833275_P01 | Uncharacterized protein LOC100384167 | |  |
| GRMZM5G835463_P01 | Uncharacterized protein  LOC103652158 | | |
| GRMZM5G836332_P01 | Uncharacterized protein LOC100192962 | |  |
| GRMZM5G841920_P01 | 40S ribosomal S4-3 | |  |
| GRMZM5G850924_P01 | Unknown | |  |
| GRMZM5G853974_P01 | Hypothetical protein ZEAM  MB73_Zm00001d027858 | | |
| GRMZM5G866989_P01 | Uncharacterized protein LOC100384367 | |  |
| GRMZM5G872150_P01 | Uncharacterized protein LOC100381728 | |  |
| GRMZM5G873431_P01 | Uncharacterized protein LOC100383699 | |  |
| GRMZM5G874178_P01 | Octapeptide-repeat T2-like | |  |
| GRMZM5G875135_P01 | Hypothetical protein | |  |
| GRMZM5G882080_P01 | Uncharacterized protein LOC100384561 | |  |
| GRMZM5G899476_P01 | Hypothetical protein ZEAM  MB73_Zm00001d021273 | | |

^a^ Top hit from NCBI Blast search

^b^ GO terms are given for Molecular Function (F), Biological Process (P) and Cellular Component (C) categories
